# Antiproliferative Activity of Mycalin A and Its Analogues on Human Skin Melanoma and Human Cervical Cancer Cells

**DOI:** 10.3390/md18080402

**Published:** 2020-07-29

**Authors:** Domenica Capasso, Nicola Borbone, Monica Terracciano, Sonia Di Gaetano, Vincenzo Piccialli

**Affiliations:** 1 CESTEV, University of Naples Federico II, 80145 Naples, Italy; domenica.capasso@unina.it; 2 Department of Pharmacy, University of Naples Federico II, 80131 Naples, Italy; nicola.borbone@unina.it (N.B.); monica.terracciano@unina.it (M.T.); 3 Institute of Biostructures and Bioimaging, CNR, 80134 Naples, Italy; 4 Department of Chemical Sciences, University of Naples Federico II, 80126 Naples, Italy

**Keywords:** Mycalin A, C_15_ acetogenins, synthetic analogues, antiproliferative activity, A375 and HeLa cell lines

## Abstract

Mycalin A, a polybrominated C_15_ acetogenin isolated from the encrusting sponge *Mycale rotalis*, displays an antiproliferative activity on human melanoma (A375) and cervical adenocarcinoma (HeLa) cells and induces cell death by an apoptotic mechanism. Various analogues and degraded derivatives of the natural substance have been prepared. A modification of the left-hand part of the molecule generates the most active substances. A structurally simplified lactone derivative of mycalin A, lacking the C1–C3 side chain, is the most active among the synthesized compounds exhibiting a strong cytotoxicity on both A375 and HeLa cells but not but not on human dermal fibroblast (HDF) used as healthy cells. Further evidence on a recently discovered chlorochromateperiodate-catalyzed process, used to oxidise mycalin A, have been collected.

## 1. Introduction

C_15_ acetogenins are typical metabolites from red algae belonging to the genus *Laurencia*. They are usually halogenated substances including one or more cyclic ethers with different ring sizes and an enyne or a bromoallene terminal unit [1]. Linear C_15_ acetogenins are less common [1,2], and some of them, such as laurencenyne and *trans*-laurencenyne, have been hypothesized to be precursors of their cyclic congeners [3]. The investigation of specimens of algae *Laurencia* genus collected in different geographical areas continues to provide new C_15_ acetogenins displaying a large variety of substitution patterns and structural complexity, and even rearranged skeletons [4].

**Figure 1:**
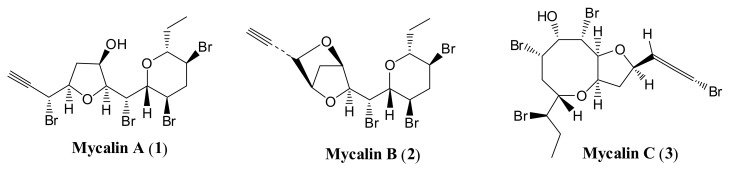
C_15_ acetogenins isolated from the sponge *Mycale rotalis*.

In 1990 three new members of this class of substance (mycalin A–C, **1**–**3**, Figure 1) (†) were isolated by our group from the encrusting sponge *Mycale rotalis* [5,6] collected in Italy along the Sicily coast. This finding was rather unusual and was explained by hypothesizing that the sponge could preferentially grow on an alga of the *Laurencia* species engulfing it entirely. This agrees well with the findings by Rinehart et al. [7] who had previously shown that metabolites peculiar to *Laurencia* species could be detected from other marine organisms.

Mycalin A (**1**) is the most abundant metabolite isolated from the natural source. It possesses an unusual terminal bromopropargylic side chain, *cis*-THF (tetrahydrofuran) and *cis*-THP (tetrahydropyran) rings and four bromine atoms, a unique structural feature among this kind of metabolite [1]. Mycalin A has subsequently been isolated from two algae of *Laurencia* genus namely *Laurencia paniculata* [8] (‡), collected at Çeşmealt near Izmir (Turkey) and *Laurencia obtusa* [9] (§), collected in the Greek Ionic sea. The former organism also contained mycalin B [8] (‡) (**2**, Figure 1), a substance closely related to mycalin A. These findings support the hypothesis about the algal origin of the mycalins. The structural determination of compounds **1–3** was accomplished through spectral evidence and chemical correlation and derivatization work, and their relative and absolute stereochemistry was firmly established by X-ray analyses.

C_15_ acetogenins have exhibited a broad range of biological properties including anti-inflammatory [10] and anti-tumor activities, among others [1]. In this paper we report that mycalin A (**1**) and some of its synthetic analogues (**4**–**11**) possess a strong antiproliferative activity on human melanoma (A375) and human cervical adenocarcinoma (HeLa) cells. A degraded C4 lactone derivative of mycalin A (**11**) has displayed a remarkable selective cytotoxicity towards tumor cells in comparison with healthy control cells.

## 2. Results and Discussion

### 2.1. Biological Assays on Mycalin A

**Figure 2:**
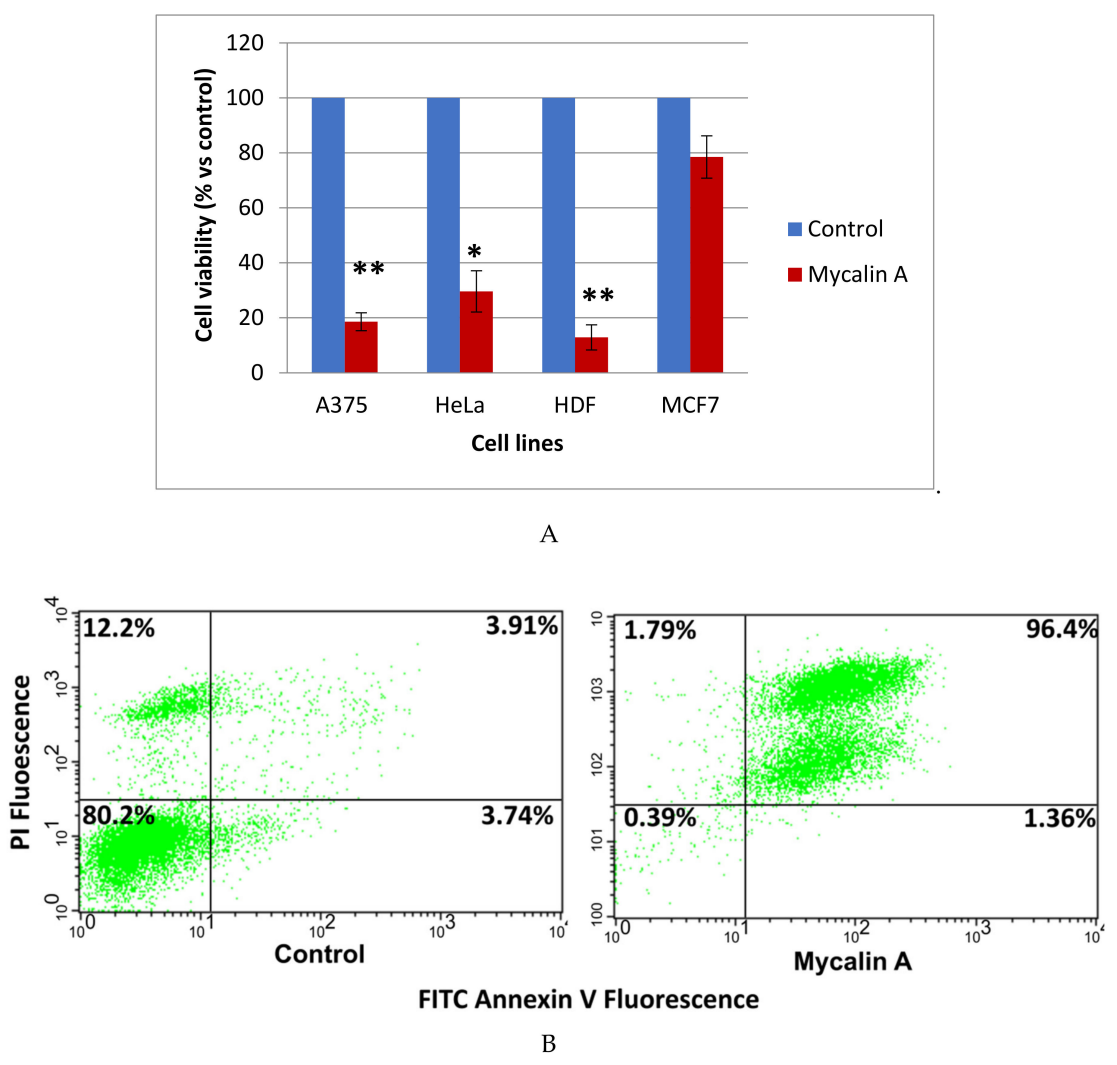
(**A**) Effect of mycalin A on tumor and healthy cell proliferation. The cells were incubated in the presence of 10 µM mycalin A, at 37 °C. Blue bars: control (vehicle treated cells); red bars: treated cells. Error bars represent ± SE (standard error), ** *p* < 0.01, * *p* < 0.05. (**B**) Apoptosis analysis with the annexin V-FITC (V-fluorescein isothiocyanate)/PI (propidium iodine) double staining method on A375 cells. The cells were treated with mycalin A (**1**), at 37 °C, at 10 µM concentration, for 48 h. The control is the vehicle treated sample. Lower left quadrant: viable cells; upper left: necrotic cells; upper right: advanced apoptotic cells; lower right: early apoptotic cells. This picture is representative of two independent experiments.

The search for new biologically active compounds is an important objective in medicinal chemistry and historically the marine environment has furnished a great number of natural products some of which have served as lead compounds for drug development [11]. As a part of our ongoing interest on the search for new anti-tumor substances, either of synthetic or natural origin [12,13,14], the effect of mycalin A (**1**) has been evaluated on the proliferation of human tumor cell lines of different histological origin. In particular, human malignant melanoma (A375), human cervical adenocarcinoma (HeLa) and human breast cancer (MCF-7) cells have been investigated. Human dermal fibroblasts (HDF) were used as healthy cells to evaluate the selectivity of action of the examined compound. All the cells were incubated with mycalin A at a 10 µM concentration for 48 h. As shown in Figure 2A, mycalin A (**1**) induced a strong inhibition on the proliferation of A375 and HeLa cells of about 80% and 70%, respectively. Interestingly, a low inhibition was observed on MCF-7 cells, which indicates the selectivity of **1** towards specific tumor cell lines, an attractive characteristic in terms of cancer therapy. Nonetheless, a very strong toxicity was observed against the healthy HDF cells.

As a next step, the ability of mycalin A to induce cell death through an apoptotic mechanism was tested on the A375 cell line that had shown the best response in terms of cytotoxicity among all the studied cells. The cell sample was treated with compound **1** at a 10 µM concentration for 48 h and then the apoptosis analysis was performed with the annexin V-FITC (V-fluorescein isothiocyanate)/propidium iodine (PI) double staining method using flow cytometry analysis. In this experiment, FITC-labelled annexin V, that specifically identifies apoptotic cells, was used in combination with PI, that is able to stain positively late stage apoptotic and necrotic cells. Therefore, the necrotic cells stain with only PI, advanced apoptotic cells with PI and FITC, and early apoptotic cells with only FITC. The results indicated that the entire population of the cells treated with mycalin A was in the late apoptotic stage (Figure 2B). This result is in agreement with the high anti-proliferative activity shown by **1** on the A375 cells under the same conditions (Figure 2A).

**Figure 3:**
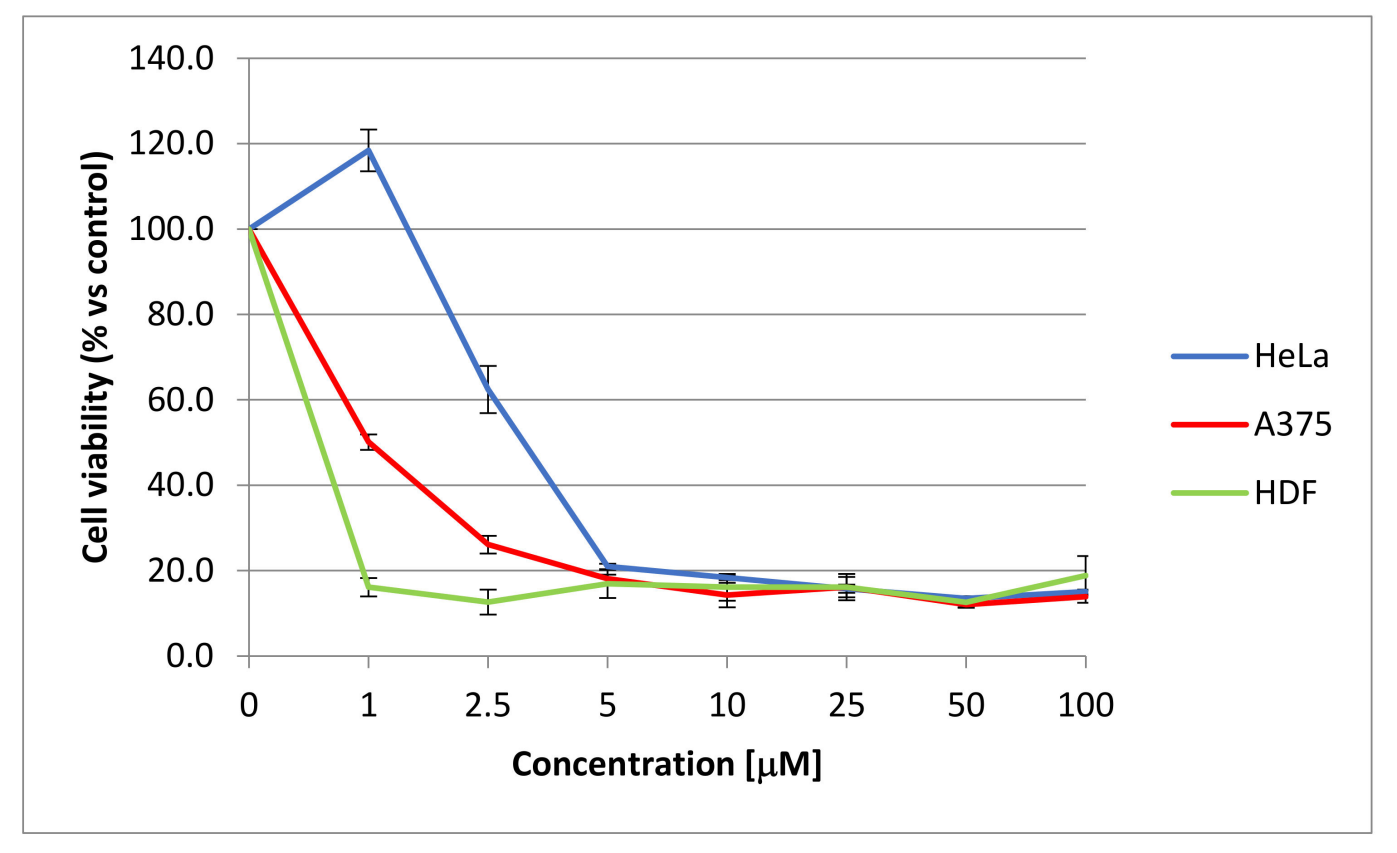
Dose-response curves obtained using the indicated concentrations of mycalin A (**1**) on responsive cells. The proliferation was determined by MTT assays. The results are presented as the percentage of proliferating cells compared to the control and are expressed as means ± SE of at least three independent experiments performed in triplicate. Statistical analyses: on HeLa *p* < 0.05 at 2.5 µM and *p* < 0.01 from 5 to 100 µM, on A375 *p* < 0.01 from 1 to 100 µM and on HDF *p* < 0.01 from 1 to 100 µM.

Furthermore, the dose–response curves (Figure 3) and the corresponding IC_50_ values, calculated on responsive cells (A375, HeLa and HDF, Table 1), confirm the high activity of mycalin A and its lack of selectivity towards tumor cells.

### 2.2. Synthesis of Analogues of Mycalin A

**Figure 4:**
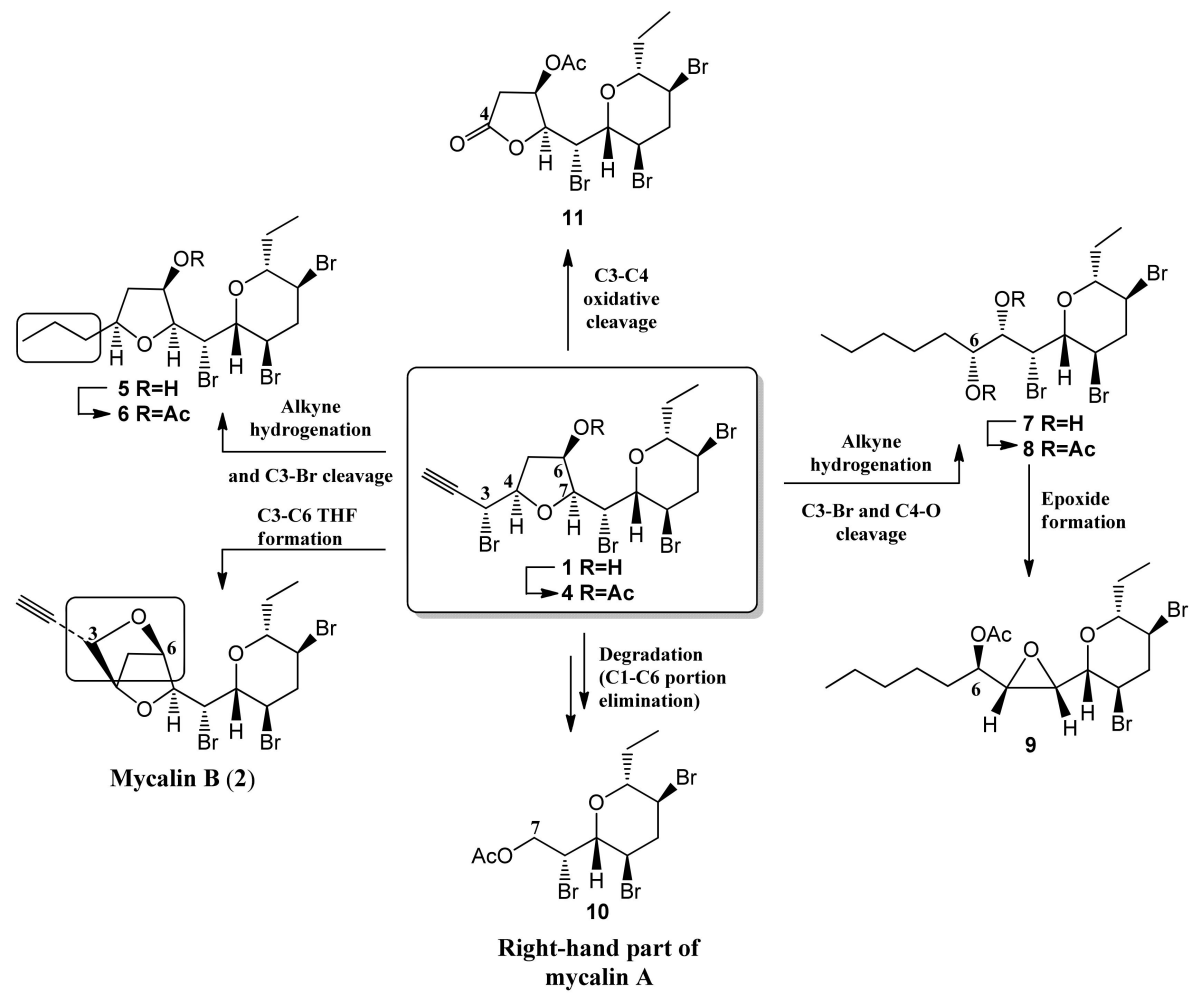
Overview of mycalin A derivatives.

The above interesting results on the activity of mycalin A prompted us to design simplified analogues of mycalin A which could retain the antitumor activity of the parent compound while exhibiting a limited toxicity towards healthy cells. To achieve this objective, mycalin A was subjected to a modification of its functional groups and a structural simplification, including degradation. An overview of the prepared derivatives **2** and **4**–**11** is shown in Figure 4. A detailed explanation of the chemistry follows.

**Scheme 1:**
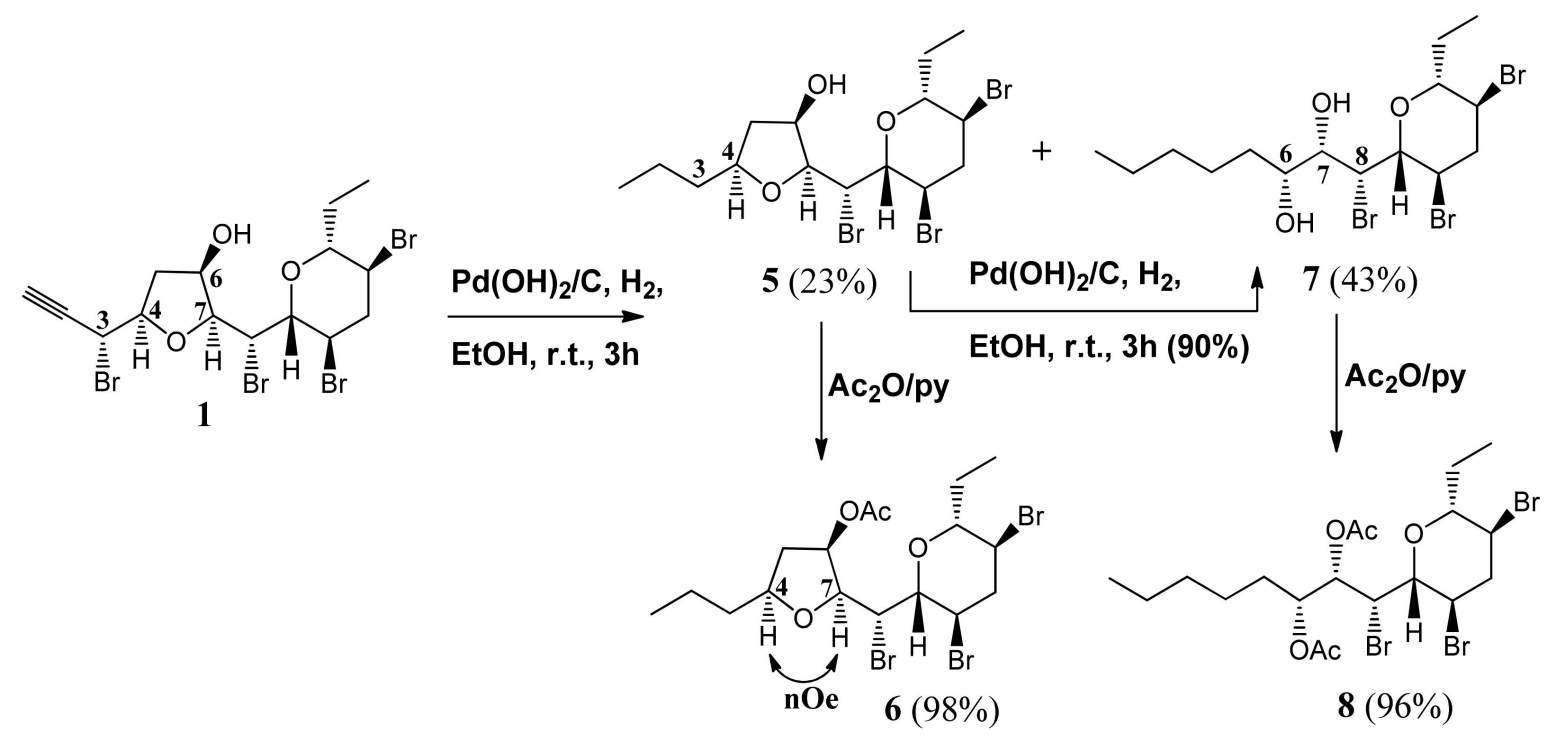
Hydrogenation of mycalin A (**1**). The H-4/H-7 nOe (nuclear Overhauser effect) correlation in **6** is highlighted.

**Scheme 2:**
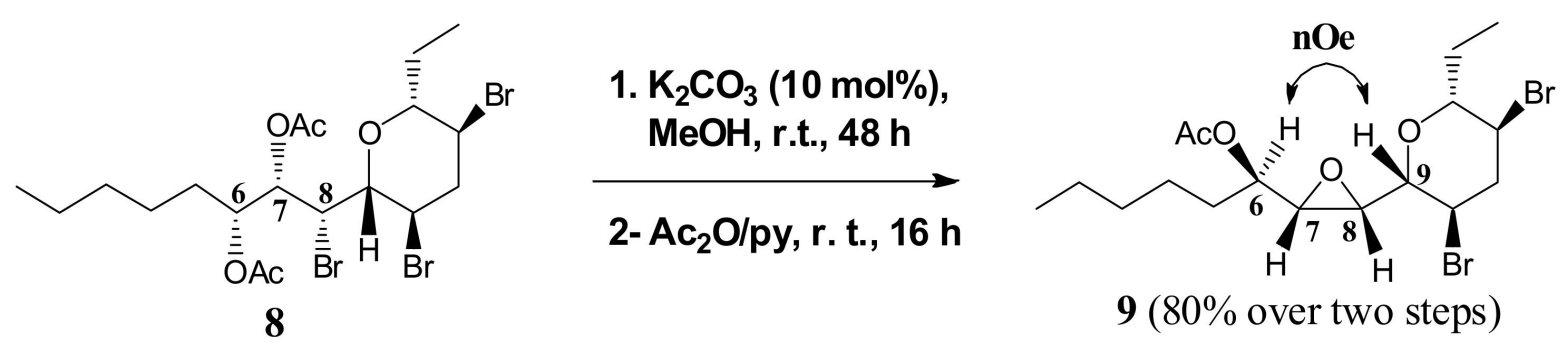
Preparation of epoxide **9**. The H-6/H-9 nOe correlation is highlighted.

**Figure 5:**
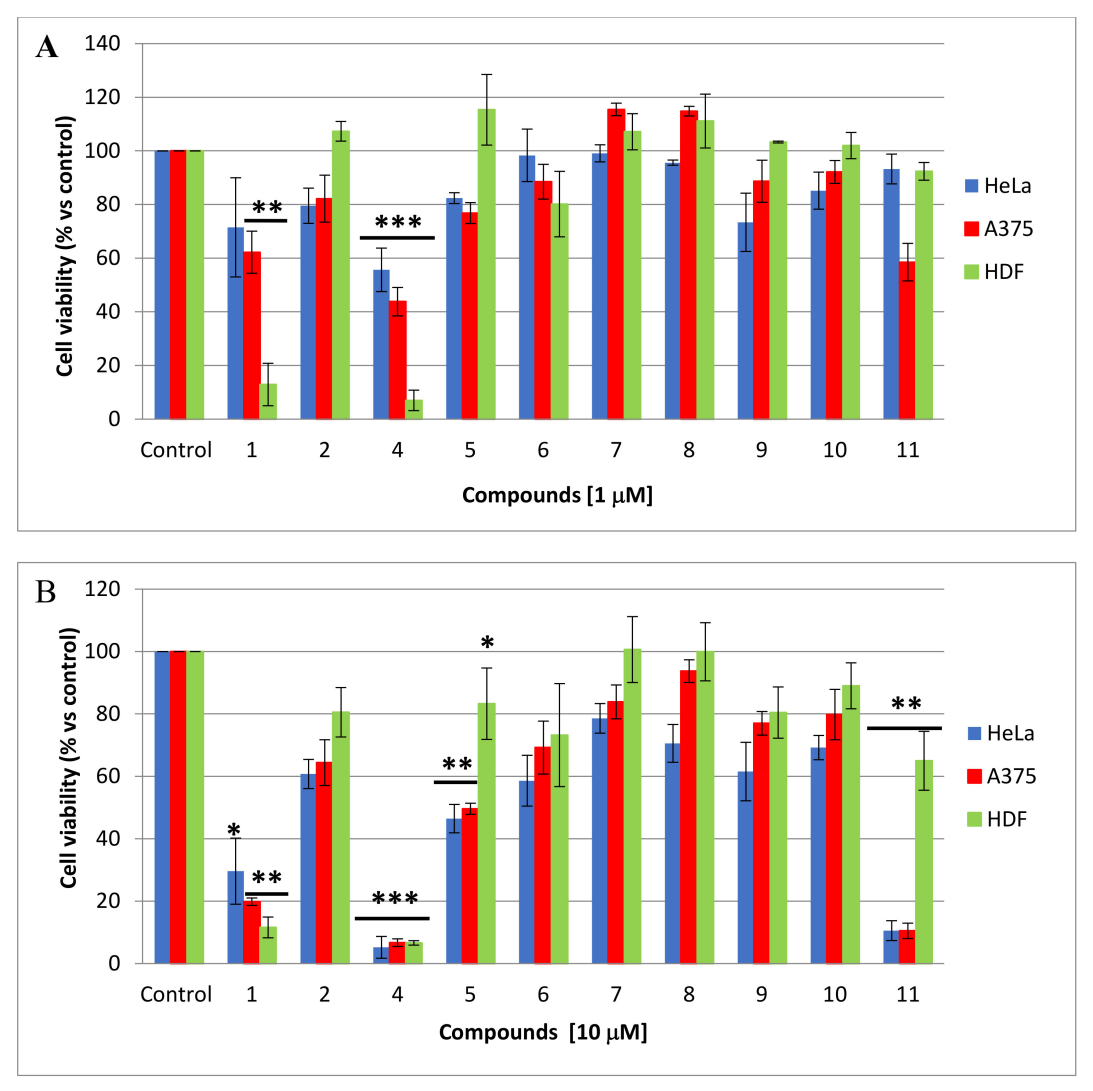

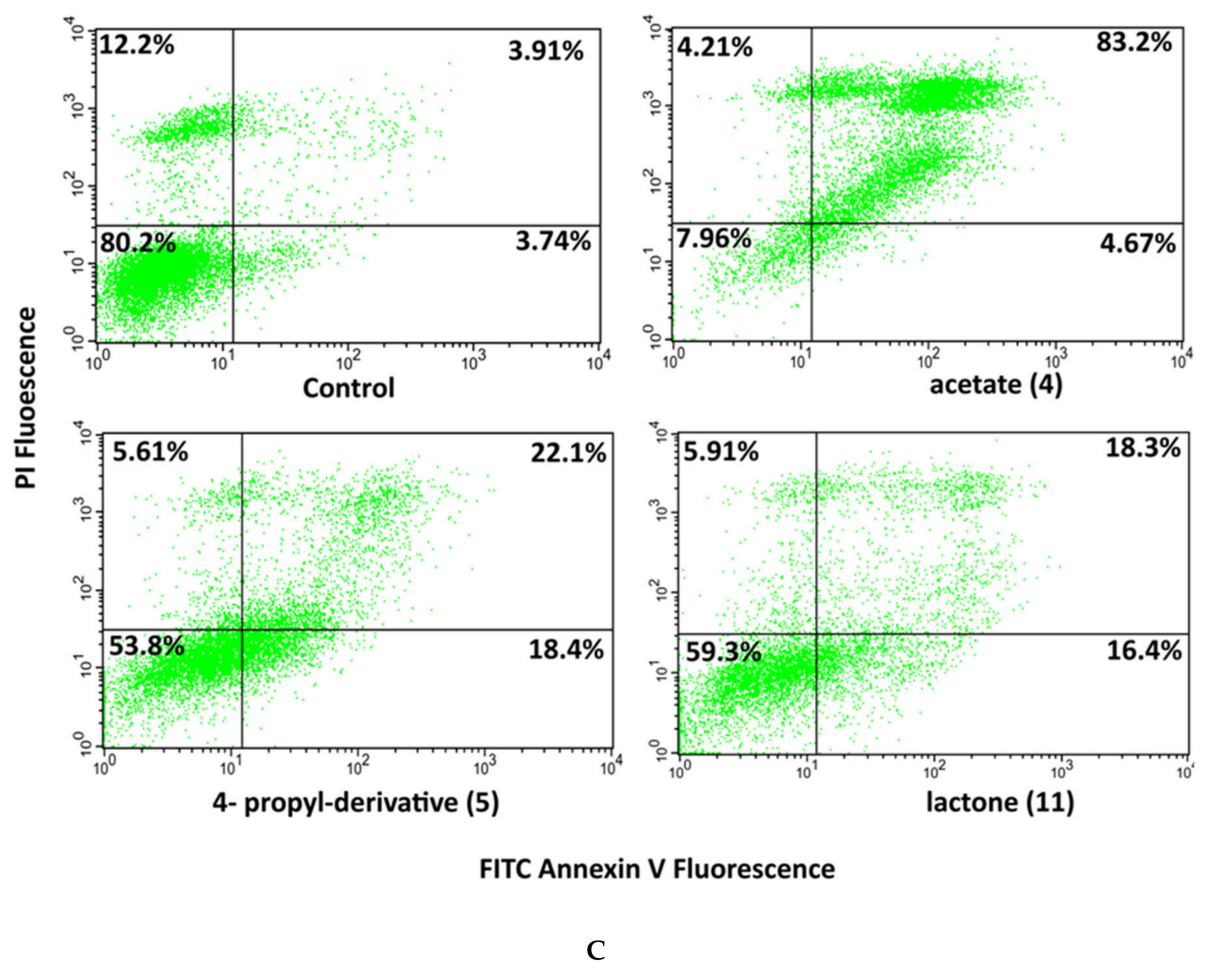
Effect of mycalin A and its derivatives on tumor and normal cell proliferation. The A375, HeLa and HDF cells were incubated in the presence of compounds **1**, **2** and **4**–**11**, solubilized in dimethyl sulfoxide DMSO (dimethyl sulfoxide), at 1 µM (**A**) or 10 µM (**B**) concentrations, at 37 °C for 48 h. Error bars represent ± SE; *** *p* < 0.001 ** *p* < 0.01 * *p* < 0.05 (**C**) Apoptosis analysis with the annexin V-FITC/PI double staining method on A375 cells. The cells were treated with the indicated compounds, at 37 °C, at 10 µM concentration, for 48 h. The control is the vehicle treated sample. Lower left quadrant: viable cells; upper left: necrotic cells; upper right: advanced apoptotic cells; lower right: early apoptotic cells. This picture is representative of two independent experiments.

Acetylation is a simple way to decrease the polarity of a substance and, potentially, to facilitate its penetration into the cell membrane. Thus, mycalin A was acetylated under standard conditions with Ac_2_O/pyridine at 50 °C for 3 h, to give acetylated mycalin A **4** (Figure 4). Indeed, this simple structural modification further increased the antitumor potency of this substance (see later Figure 5A,B). Other potential sites of reactivity were then taken into consideration, such as the triple bond as well as the C-Br and ethereal C-O bonds. In this respect, the left-hand moiety of mycalin A proved to be the most easily modifiable. It is well known that ethereal C-O and C-Br bonds can be reductively cleaved by catalytic hydrogenation under standard conditions. Based on this consideration and literature precedents [15], we were confident that compound **1** could be structurally simplified when subjected to hydrogenation, possibly even giving open chain mycalin A analogues. Indeed, when **1** was subjected to catalytic hydrogenation with Pd(OH)_2_/C (10%) in EtOH, a mixture of compounds **5** and **7** was obtained in the approximate ratio of 1:2, in favor of **7** (Scheme 1). Prolonged reaction times only resulted in higher amounts of **7**, suggesting that **5** at least partly converts into **7**. Accordingly, compound **5** could be transformed into **7** in high yields under the same reaction conditions (Scheme 1). The 4-propyl analogue **5** derived from the alkyne saturation and the C3-Br reductive cleavage. Further cleavage of the C4-O bond of the THF ring of **1** gave the open-chain diol **7**. This favorable selectivity concerning the C-Br and ethereal C-O bond cleavage allowed us to obtain compound **7** with the intact THP ring and a functionalization that paved the way for the preparation of other THP derivatives, thus permitting us to access mycalin A right-hand model compounds. In particular, the presence of a 7,8-halohydrin subunit in **7** allowed us the preparation of the corresponding 7,8-epoxide analogue (Scheme 2), thus increasing the functional group diversity and, taking into account the biological activity exhibited by a variety of natural epoxides, the probability of accessing a cytotoxic substance.

The structures of **5** and **7** were confirmed by extensive 2D NMR (two-dimensional nuclear magnetic resonance) experiments on the corresponding acetate derivatives **6** and **8**, respectively, which were obtained under usual acetylation conditions (Scheme 1). The complete proton and carbon assignments of **6** and **8** are reported in Table S1 and S2, respectively. In particular, the presence of a strong nOe correlation between the H-4 and H-7 protons in **6** (Scheme 1 and Figure S12) confirmed that the *cis* configuration of the THF ring had been preserved under the hydrogenation conditions. On the other hand, the replacement of the methine proton signal resonating at δ 3.96 (H-4) in **6** (Scheme 1) with the methylene signal resonating at δ 1.26 (H_2_-4) in **8** (Scheme 1), confirmed the cleavage of the THF ring at the C4-O bond in **1**. Based on the retention of the *S* configuration at C7 on reductive opening of the THF ring, the 7*S*,8*R* configuration could be formulated for the 7,8-halohydrin system in **7**.

Epoxide **9** was obtained on treatment of diacetate **8** with catalytic amounts of K_2_CO_3_ in MeOH, followed by acetylation (Scheme 2). The presence of an nOe correlation between H-6 and H-9 protons in **9** (Scheme 2; see Table S3 for the complete NMR assignment), pointed to the *cis* configuration of the epoxide ring and, consequently, to the all *syn* arrangement of the C6–C8 segment in **8**. Therefore, we could assign the 6*R*,7*S*,8*S* configuration at the C6–C8 stereocentres in **9**.

**Scheme 3:**
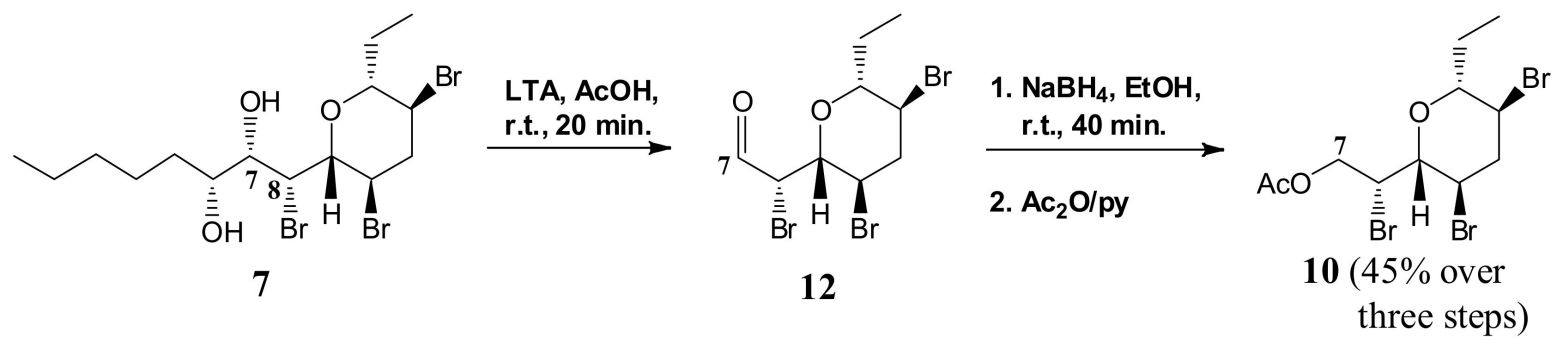
Synthesis of the degraded derivative **10**.

The presence of the C6-C7 diol system in **7** allowed us to achieve the synthesis of the structurally simplified THP derivative **10** (Scheme 3), lacking the THF-containing left-hand (C1–C6) portion of **1**. To this end, first the oxidative cleavage of **7** was attempted by treatment with NaIO_4_ in CH_2_Cl_2_/H_2_O (1:1). However, a slow process and low yields of the expected aldehyde **12** (Scheme 3) resulted. Similar results were obtained by using acetone/water as solvent. Likewise, silica supported NaIO_4_ also gave unsatisfactory results. Eventually, the treatment of **7** with lead tetraacetate (LTA) in acetic acid quickly gave aldehyde **12** as the sole product whose immediate reduction with NaBH_4_ in EtOH, followed by acetylation, afforded the degraded product **10** in 45% yield, over three steps (Scheme 3; Table S4).

**Scheme 4:**
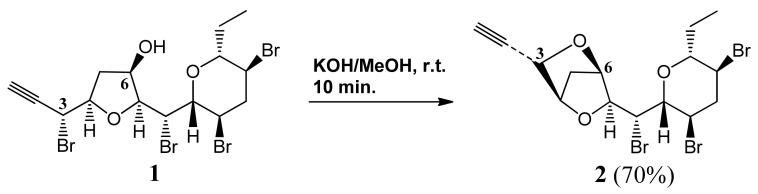
Synthesis of mycalin B **2**.

Mycalin B was then prepared (Scheme 4). This substance, possessing a further THF ring including part of the C4 side chain and the C6 OH group, introduces a structural perturbation in the left-hand moiety of the mycalin A. Therefore, it was seen as a compound able to provide information on the possible involvement of the left-hand part of mycalin A functional groups in its pharmacophoric portion. Thus, compound **1** was treated with KOH in MeOH at r.t. The internal nucleophilic substitution was very fast, and compound **2** was obtained in a 70% yield. This substance showed spectroscopic (Figures S3 and S4) and chromatographic properties identical to those exhibited by an authentic sample of mycalin B [5].

**Scheme 5:**
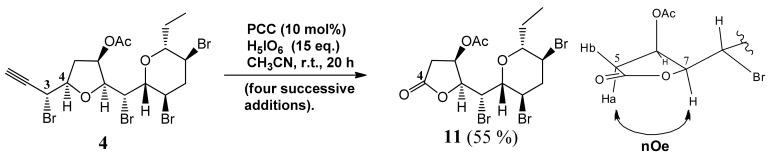
Synthesis of lactone **11**. The 3D lactone ring part structure and the Ha-5/H-7 nOe correlation are highlighted on the right.

Lastly, lactone **11** was prepared by using a PCC (pyridinium chlorochromate)-catalyzed process (PCC/H_5_IO_6_ system) recently discovered in our laboratory [16], starting from mycalin A acetate (Scheme 5). Okamura et al. [17] have postulated that the actual oxidizing agent, under the above conditions, is chlorochromateperiodate (CCP), an oxidizing species more powerful than PCC, derived from the condensation of PCC and H_5_IO_6_ with the elimination of water. As we observed for the preparation of the corresponding benzoate [12], this process failed to go to completion under previously used conditions. However, in this instance we were able to force the process up to the complete consumption of the starting material by means of three further additions of freshly prepared CCP. In this way, lactone **11** was obtained in a 55% isolated yield. The loss of the C1-C3 carbon chain and the formation of the lactone ring was confirmed by ^13^C-NMR (Figure S34) and HMBC (Figure S38) spectra. An nOe correlation between Ha-5 and H-7 protons in **11** further confirmed the configuration at C-7 (Scheme 5 right and Figure S36). Due to the limited amount of mycalin A, no further optimization of the process could be carried out at this stage. However, the observed behavior suggests a quick destruction of the active species and this process is certainly worth further investigation. Considering the synthetic importance of γ-lactones and the biological activity displayed by a variety of natural substances belonging to this class of compound, further studies are currently in progress in our laboratories.

A summary of the synthesized mycalin A derivatives is shown in Table 2.

### 2.3. Biological Assays

To evaluate the cytotoxic activity of compounds **2** and **4**–**11** on the A375, HeLa and HDF cells, an initial screening was carried out at 1 µM and 10 µM concentrations for 48 h (Figure 5A,B). The cell viability was measured by means of the MTT assay. Among all the tested compounds, a remarkable cytotoxic activity was exhibited by acetate **4** and lactone **11** on the A375 and HeLa cells. Therefore, the acetylation of **4** increased the cytotoxicity of mycalin A towards the A375 and HeLa cells even at 1 µM, reaching a prominent effect at 10 µM. It is to be said that compound **4** also showed a toxic effect towards healthy cells at both the used concentrations. On the other hand, lactone **11** caused a drastic decrease in the cell viability up to 10%, compared to the control, at a dose of 10 µM on both cell lines. In addition, differently from acetate **4**, lactone **11** showed a considerable selectivity towards tumor cells, with a cytotoxicity of only about 35% on the HDF cells. Among the remaining compounds, 4-propyl-derivative **5** showed a good cytotoxicity on both the tumor cells and only a slight toxicity on the healthy cells, at a 10 µM concentration.

Then, FITC-AnnexinV assays were performed on the A375 cell line to evaluate the ability of **4**, **5**, and **11** to induce cell death through apoptosis. The cell sample was treated with such compounds at a 10 µM concentration for 48 h. The results indicated that all the tested compounds induced apoptotic effects (Figure 5C). In particular, **4** showed the entire population in late apoptosis, as observed for mycalin A. Differently, **5** and **11** displayed a lower level of apoptosis, at the same concentration.

These data indicate that the THF-containing substances are the most active and that the absence of the THF portion strongly reduces the activity, as shown by the THP derivatives **7**–**10** which display the lowest cytotoxic activity. This suggests that the THF-containing portion of mycalin A is essential for the activity. However, this does not mean that it alone is sufficient for the activity. The synthesis of further simplified analogues, only containing the left-hand part of the molecule, may help to clarify this point. The fact that mycalin B (**2**), where the C4 side-chain and the C6-OH group are engaged in a further THF ring, exhibits a reduced cytotoxicity compared with mycalin A, further supports the importance of the THF-containing, or THF-mimicking, portion of the substance and also suggests that the side-chain and/or the hydroxyl group could play a role in the cytotoxic effect of mycalin A.

**Figure 6:**
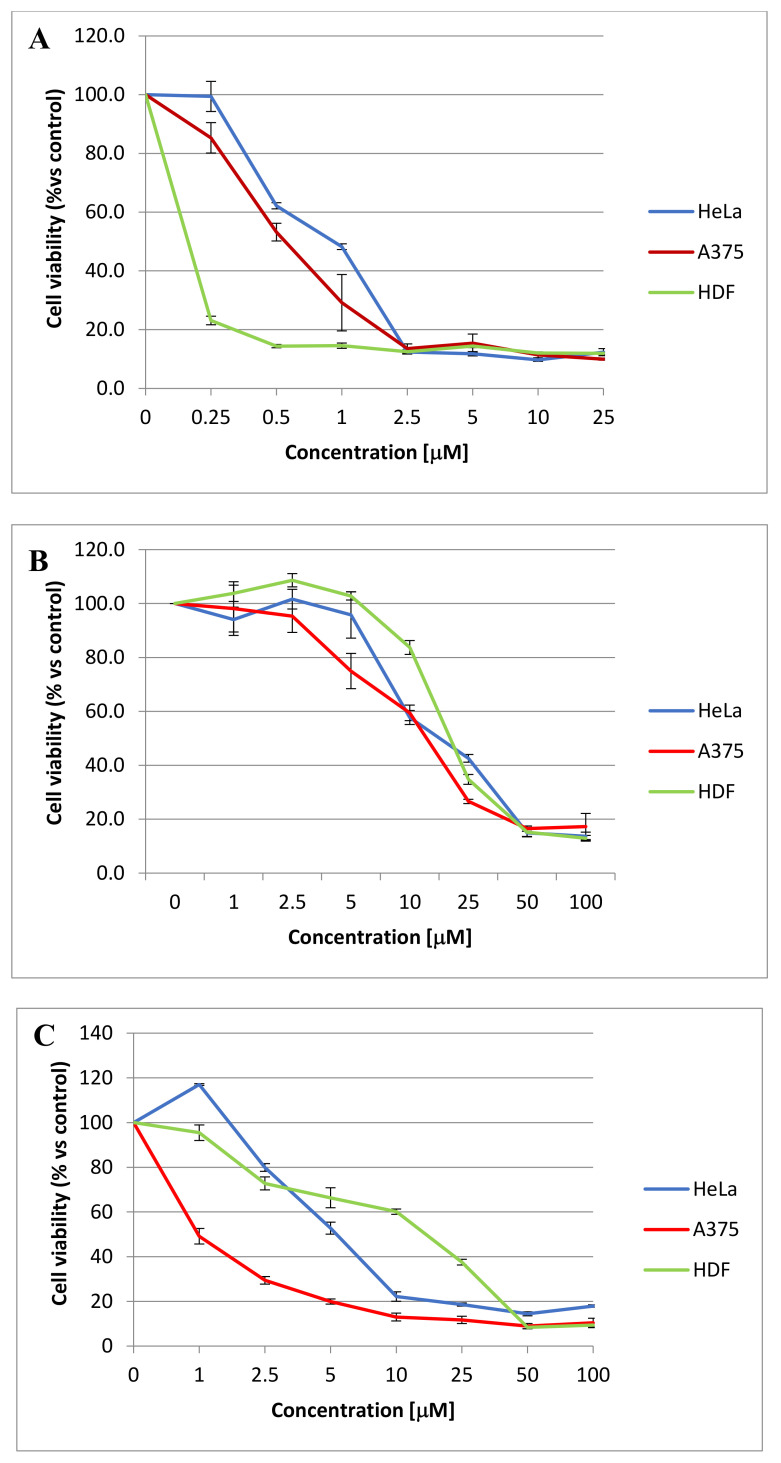
Dose-response curves of **4** (**A**), **5** (**B**), and **11** (**C**). The compounds were tested at different concentrations on the indicated cell lines. The proliferation was evaluated using the MTT assay. The results are presented as the percentage of proliferating cells with respect to the control (vehicle treated cells) and are expressed as means ± SE of at least three independent experiments performed in triplicate. Statistical analysis **4**: on HeLa *p* < 0.001 from 0.5 to 25 µM, on A375 *p* < 0.01 from 0.5 to 25 µM, on HDF *p* < 0.001 from 0.25 to 25 µM. **5**: on HeLa *p* < 0.01 from 10 to 100 µM, on A375 *p* < 0.01 at 10 and 25 µM, *p* < 0.05 at 50 and 100 µM, on HDF *p* < 0.05 at 10 µM and *p* < 0.001 from 25 to 100 µM. **11**: on HeLa *p* < 0.01 from 2.5 to 100 µM, on A375 *p* < 0.05 at 2.5 µM, *p* < 0.01 from 5 to 100 µM, on HDF *p* < 0.01 at 10 µM and *p* < 0.05 from 25 to 100 µM.

Prompted by the results obtained by the anti-proliferative assays, the bioactivity profile of the most interesting compounds **4**, **5**, and **11** was evaluated using a concentration ranging from 0.25 μM to 25 μM for **4** and from 1 μM to 100 μM for **5** and **11**. The results are depicted in Figure 6A–C. From these dose–response analyses, the IC_50_ for each substance could be evaluated (Table 3) and some observations could be made. Acetate **4** showed a high cytotoxicity upon all the tested cells, including the healthy cells and its IC_50_ values confirm the increased activity compared to mycalin A. Compound **5** showed comparable IC_50_ values on the tumor cells as well as on the healthy cells and consequently lacking selectivity. Finally, compound **11** proves to be a very interesting substance, showing an IC_50_ of about 1 µM towards the melanoma (A375) cell line and an IC_50_ on HDF cells that is an order of magnitude higher. Remarkably, **11** retained the same activity of **1** on tumor cells, further increasing its selectivity.

## 3. Materials and Methods

### 3.1. General Experimental Procedures

All reagents were purchased (Aldrich and Fluka) at the highest commercial quality and used without further purification. The reactions were monitored using thin layer chromatography (TLC) carried out on precoated silica gel plates (Merck 60, F254, 0.25 mm thick). Merck silica gel (Kieselgel 40, particle size 0.063–0.200 mm) was used for the column chromatography. All the HPLC (high performance liquid chromatography) separations and purifications were performed on a Phenomenex Luna column (25 cm × 4.6 mm, 5 µm) using *n*-hexane/EtOAc mixtures as eluent, at a flow rate of 1 mL/min. Na_2_SO_4_ was used as a drying agent for aqueous work-up. Nuclear magnetic resonance (NMR) experiments were performed using either a Bruker 700 MHz AvanceNeo spectrometer (Billerica, MA, US) equipped with a triple resonance cryoprobe or a Bruker 400 MHz Avance spectrometer (Billerica, MA, US) in CDCl_3_. Proton chemical shifts were referenced to the residual CHCl_3_ signal (7.26 ppm). ^13^C-NMR chemical shifts were referenced to the solvent (77.0 ppm). Coupling constants (J) are given in Hertz. Abbreviations for signal coupling are as follows: s = singlet, d = doublet, t = triplet, q = quartet, m = multiplet, b = broad. The optical rotations were measured using a JASCO P-2000 polarimeter (JASCO, Oklahoma City, OK, US) at the sodium D line. The high-resolution mass spectra were recorded by infusion on a Thermo Linear Trap Quadrupole (LTQ) Orbitrap XL mass spectrometer (Thermo Fisher Scientific, Waltham, MA, US) equipped with an electrospray source in the positive mode using MeOH as the solvent.

### 3.2. Mycalin A 1

Mycalin A (**1**) was isolated from the sponge *Mycale rotalis* collected in the Stagnone di Marsala lagoon (Sicily) in the spring of 1989 [5,6]. For details of the isolation of mycalin A see ref. 6. A pure sample of mycalin A for the biological assays was obtained by filtration on a silica gel pad (eluent *n*-hexane-EtOAc, 4:6) followed by HPLC purification using *n*-hexane-EtOAc (75:25) as the eluent. Copy of the NMR spectra are reported in the Supplementary Materials.

### 3.3. Mycalin A Acetate 4

Excess acetic anhydride (0.5 mL) was added to a solution of alcohol **1** (18.2 mg, 0.032 mmol) in pyridine (0.5 mL) and the mixture stirred at 50 °C. After 3 h the reaction mixture was evaporated under reduced pressure to give acetyl derivative **4** (19.5 mg) as a single spot by TLC (*n*-hexane-EtOAc, 7:3, Rf = 0.56) The crude was subjected to HPLC purification using *n*-hexane-EtOAc, 8:2 as the eluent to give **4** (18.0 mg, 93%) as a colourless oil. **4**: ${\left[\alpha \right]}_{D}^{20}$ = + 41.1 (c = 0.20, CHCl_3_). IR (infrared) (neat) λ_max_ 2121, 1737, 1374, 1241, cm^−1^. ^1^H-NMR (400 MHz, CDCl_3_): δ 5.27 (1H, bdd, *J* = 5.4, 3.1), 4.65 (1H, dd, *J* = 9.5, 1.5), 4.59 (1H, dd, *J* = 6.7, 2.3), 4.34 (1H, ddd, 8.8, 6.6, 4.8), 4.24 (1H, dd, *J* = 9.6, 3.2), 4.17 (1H, ddd, *J* = 12.3, 9.5, 4.5), 3.72 (1H, ddd, *J* = 11.9, 10.2, 4.4), 3.39 (1H, dt, *J* = 9.5, 2.3), 3.21 (1H, dd, *J* = 9.6, 1.5), 2.98 (1H, ddd, *J* = 12.9, 4.4, 4.4), 2.68–2.59 (2H, overlapped m’s), 2.39 (1H, ddd, *J* = 12.6, 12.6, 12.6), 2.29 (1H, dd, *J* = 15.6, 5.0), 2.12 (3H, s, acetate), 2.02 (1H, m), 1.54 (1H, m), 0.93 (3H, t, *J* = 7.3). ^13^C-NMR: (100 MHz, CDCl_3_): δ 170.1, 83.7, 83.6, 80.2, 79.7, 79.3, 76.5, 72.1, 51.5, 47.1, 46.6, 45.1, 37.4, 37.0, 25.7, 21.2, 9.5. HRESIMS (high resolution electrospray ionization mass spectrometry) *m*/*z*: calcd for C_17_H_22_^79^Br_4_NaO_4_ 628.8149 [M + Na]^+^, found: 628.8140.

### 3.4. Propyl Derivative 5

Pd(OH)_2_/C (20% *w/w*, 8.5 mg) was added to mycalin A (**1**, 42.3 mg, 0.074 mmol) in EtOH (2 mL) and the mixture stirred under hydrogen atmosphere. A vacuum-fill technique was used using a hydrogen balloon and a three-way vacuum adapter. After 2 h the reaction mixture was filtered over celite, and the filtrate was dried under reduced pressure to give a mixture of the propyl-derivative **5** and the diol **7** (40.5 mg) as a colorless oil. Further filtration of the crude on a silica gel pad, eluting with *n*-hexane-EtOAc (7:3), afforded an oily material (29.8 mg), essentially constituted by a mixture of **5** and **7** (TLC: *n*-hexane-EtOAc, 7:3. **5**: Rf = 0.60; **7**: Rf = 0.67). HPLC separation with *n*-hexane-EtOAc (7:3) as the eluent gave pure compounds **5** (8.2 mg, 23%) and **7** (15.9 mg, 43%) as colourless oils. **5**: ${\left[\alpha \right]}_{D}^{20}$ = +4.2 (c = 0.17, CHCl_3_). IR (neat) λ_max_ 3446 (broad) cm^−1^. ^1^H-NMR: (400 MHz, CDCl_3_): δ 4.64 (1H, dd, *J* = 9.2, 1.8), 4.24–4.14 (2H, overlapped m’s), 3.96–3.87 (2H, overlapped m’s), 3.81 (1H, dd, *J* = 9.8, 1.9), 3.76 (1H, ddd, *J* = 12.1, 10.0, 4.3), 3.44 (1H, dt, *J* = 9.1, 2.3), 3.00 (1H, ddd, *J* = 12.9, 4.3, 4.3), 2.48 (2H, overlapped m’s), 2.04 (1H, m), 1.77 (1H, m), 1.68–1.33 (5H, overlapped m’s), 0.96 (3H, t, *J* = 7.2), 0.94 (3H, t, *J* = 7.2). ^13^C-NMR: (100 MHz, CDCl_3_): δ 84.8, 83.8, 80.0, 71.9, 53.3, 47.7, 46.8, 45.3, 42.2, 38.9, 25.8, 19.2, 14.0, 9.7. HRESIMS *m*/*z*: calcd for C_15_H_25_^79^Br_3_NaO_3_ 512.9252 [M + Na]^+^, found: 512.9240.

### 3.5. Propyl Acetate 6

Acetic anhydride (0.4 mL) was added to a stirred solution of **5** (8.2 mg, 0.017 mmol) in pyridine (0.4 mL), at room temperature. After 16 h the reaction mixture was evaporated under reduced pressure to give acetyl derivative **6** (9.0 mg) as a colourless oil. HPLC purification (eluent: *n*-hexane-EtOAc, 7:3) gave the pure compound **6** (8.8 mg, 98%) suitable for the biological assays. **6**: ${\left[\alpha \right]}_{D}^{20}$ = +15.3 (c = 0.71, CHCl_3_). IR (neat) λ_max_ 1737, 1375, 1239 cm^−1^. ^1^H-NMR: (700 MHz, CDCl_3_): δ 5.19 (ddd, *J* = 6.3, 3.1, 1.1, 1H), 4.69 (dd, *J* = 9.7, 1.6, 1H), 4.17 (ddd, *J* = 12.1, 9.6, 4.6, 1H), 4.06 (dd, *J* = 9.7, 3.1, 1H), 3.96 (dq, *J* = 8.4, 6.4, 1H), 3.73 (ddd, *J* = 12.0, 10.0, 4.3, 1H), 3.38 (ddd, *J* = 10.0, 8.8, 2.4, 1H), 3.22 (dd, *J* = 9.6, 1.6, 1H), 2.98 (ddd, *J* = 13.0, 4.3, 4.3, 1H), 2.55 (ddd, *J* = 14.6, 8.4, 6.3, 1H), 2.40 (1H, ddd, *J* = 13.0, 12.1, 12.0), 2.12 (s, 3H, acetate), 2.06–1.98 (m, 1H), 1.79–1.71 (m, 1H), 1.66 (ddd, *J* = 14.5, 6.4, 1.1, 1H), 1.56–1.46 (m, 2H), 1.41 (dddd, *J* = 12.9, 10.3, 7.4, 5.5, 1H), 1.35 (dddd, *J* = 13.0, 10.6, 7.4, 5.4, 1H), 0.93 (t, *J* = 7.4, 6H, 2×Me). ^13^C-NMR: (176 MHz, CDCl_3_): δ 170.6, 83.6, 82.3, 80.2, 77.5, 73.2, 52.1, 47.4, 46.7, 45.0, 39.7, 38.2, 25.7, 21.4, 19.2, 14.1, 9.7. HRESIMS *m*/*z*: calcd for C_17_H_27_^79^Br_3_NaO_4_ 554.9357 [M + Na]^+^, found: 554.9368.

### 3.6. THP Diol 7

Compound **7** was synthesized as described above (see propyl derivative **5**). **7**: Amorphous solid. ${\left[\alpha \right]}_{D}^{20}$ = +9.9 (c = 0.57, CHCl_3_). IR (neat) λ_max_ 3414 (broad) cm^−1^. ^1^H-NMR: (400 MHz, CDCl_3_): δ 4.67 (1H, dd, *J* = 6.3, 1.7), 4.14 (1H, ddd, *J* = 12.2, 12.2, 4.5), 3.81–3.67 (4H, overlapped m’s), 3.48 (1H, ddd, *J* = 8.6, 8.6, 2.3), 3.01 (1H, ddd, *J* = 12.9, 4.5, 4.5), 2.81 (2H, bs, 2xOH), 2.47 (1H, ddd, *J* = 12.3, 12.3, 12.3), 2.04 (1H, m), 1.66–1.26 (9H, overlapped m’s), 0.95 (3H, t, *J* = 7.3), 0.90 (3H, t, *J* = 6.9). ^13^C-NMR: (100 MHz, CDCl_3_): δ 83.8, 81.4, 75.7, 71.3, 59.2, 47.1, 46.9, 45.1, 33.8, 31.7, 25.8, 25.3, 14.0, 9.5. HRESIMS *m*/*z*: calcd for C_15_H_27_^79^Br_3_NaO_3_ 514.9408 [M + Na]^+^, found: 514.9426.

### 3.7. THP Diol Acetate 8

Acetic anhydride (0.3 mL) was added to a stirred solution of diol **7** (7.0 mg, 0.014 mmol) in pyridine (0.3 mL), and the mixture stirred at 50 °C. After 3 h the reaction mixture was evaporated under reduced pressure to give diacetate **8** (8.3 mg) as a colorless oil (TLC: *n*-hexane-EtOAc, 85:15, Rf = 0.54). Pure **8** (7.9 mg, 96%), suitable for the biological assays, was obtained by HPLC purification (eluent: *n*-hexane-EtOAc, 85:15). **8**: ${\left[\alpha \right]}_{D}^{20}$ = +35.8 (c = 0.12, CHCl_3_). IR (neat) λ_max_ 1742, 1371, 1217, cm^−1^. ^1^H-NMR (700 MHz, CDCl_3_) δ 5.49 (dd, *J* = 9.5, 2.0, 1H), 5.09 (ddd, *J* = 7.7, 6.0, 2.0, 1H), 4.55 (dd, *J* = 9.4, 1.9, 1H), 4.13 (ddd, *J* = 12.1, 9.5, 4.5, 1H), 3.72 (ddd, *J* = 12.1, 10.0, 4.3, 1H), 3.47 (ddd, *J* = 10.0, 8.6, 2.4, 1H), 3.43 (dd, *J* = 9.5, 1.9, 1H), 2.96 (ddd, *J* = 12.9, 4.5, 4.3, 1H), 2.44 (ddd, *J* = 12.9, 12.1, 12.0, 1H), 2.17 (s, 3H, acetate), 2.13 (s, 3H, acetate), 2.04 (m, 1H), 1.55 (m, 1H), 1.50 (m, 1H), 1.34–1.21 (m, 8H), 0.99 (t, *J* = 7.4, 3H), 0.86 (t, *J* = 7.0, 3H). ^13^C-NMR: (176 MHz, CDCl_3_): δ 170.7, 170.0, 83.7, 79.4, 74.3, 71.6, 54.8, 47.5, 47.3, 45.0, 31.5 (2C), 25.8, 24.7, 22.4, 21.1, 20.8, 13.9, 9.7. HRESIMS *m*/*z*: calcd for C_19_H_31_^79^Br_3_NaO_5_ 598.9619 [M + Na]^+^, found: 598.9627.

### 3.8. THP Epoxyde 9

K_2_CO_3_ (3 eq, 2.2 mg, 0.016 mmol), was added to a stirred solution of diacetate **8** (3.0 mg, 0.005 mmol) in MeOH (1 mL), at room temperature. After 48 h AcOH was added up to pH = 6 and the reaction mixture was taken to dryness. The solid residue was partitioned between water and EtOAc and the organic phase was dried and evaporated to give an oil (2.9 mg). TLC analysis revealed still the presence of a trace amount of the starting diol. HPLC separation (eluent: *n*-hexane-EtOAc, 85:15) gave the pure 7-hydroxy epoxide (1.7 mg, 80%). Acetylation under usual conditions afforded acetate **9** (1.9 mg, 100%) as a colourless oil. **9**: ${\left[\alpha \right]}_{D}^{20}$ = −7.7 (c = 0.21, CHCl_3_). IR (neat) λ_max_ 1742, 1463, 1371, 1239 cm^−1^. ^1^H-NMR (700 MHz, CDCl_3_) δ 4.89 (ddd, *J* = 8.7, 8.7, 3.9, 1H), 3.84 (ddd, *J* = 12.4, 10.0, 4.4, 1H), 3.73 (ddd, *J* = 12.4, 10.1, 4.4, 1H), 3.51 (dd, J = 10.0, 7.1, 1H), 3.45 (ddd, J = 10.1, 7.3, 2.7, 1H), 3.05 (dd, *J* = 8.7, 4.1, 1H), 3.02 (dd, *J* = 7.1, 4.1, 1H), 2.98 (dt, *J* = 12.4, 4.4, 1H), 2.43 (ddd, *J* = 12.2, 12.2, 12.0, 1H), 2.11 (s, 3H, acetate), 1.98 (ddq, *J* = 14.9, 7.4, 2.7, 1H), 1.75–1.52 (m, 3H overlapped), 1.36 (m, 1H), 1.34–1.20 (m, 5H overlapped), 0.95 (t, *J* = 7.4, 3H), 0.88 (t, *J* = 7.0, 3H). ^13^C-NMR: (176 MHz, CDCl_3_): δ 170.4, 83.0, 80.5, 71.9, 57.7, 56.6, 47.1, 46.1, 45.6, 32.1, 31.8, 25.8, 24.6, 22.5, 21.1, 14.0, 9.0. HRESIMS *m*/*z*: calcd for C_17_H_28_^79^Br_2_NaO_4_ 477.0252 [M + Na]^+^, found: 477.0238.

### 3.9. Degraded THP Derivative 10

Crystalline lead tetraacetate (2 eq., 12.5 mg, 0.028 mmol) was added to a solution of diol **7** (7.0 mg, 0.014 mmol) in AcOH (1.0 mL). After 20 min., TLC analysis (*n*-hexane-EtOAc, 75:25) revealed the disappearance of the starting material and the formation of a slightly less polar substance. After a further 10 min., two drops of ethylene glycol were added and the mixture stirred for 10 min., diluted with water and extracted with CHCl_3_. The organic layer was washed with a sat. aqueous NaHCO_3_ solution, dried and evaporated to give 5.3 mg of aldehyde **12** as a smelling oil. NaBH_4_ (a tip of spatula, excess) was added to a solution of the crude aldehyde **12** (4.0 mg, 0.010 mmol) in EtOH (1 mL), and the suspension stirred at room temperature for 40 min. Then, the excess reagent was destroyed by the addition of a few drops of AcOH, water was added, and the mixture extracted with CHCl_3_. The organic phase was washed with a sat. aqueous NaHCO_3_ solution, dried and evaporated to give 4.8 mg of the crude alcohol. HPLC purification of the latter gave 3.8 mg of the pure C7 alcohol.

Acetylation of the above alcohol with Ac_2_O/pyridine (0.5 mL/0.5 mL) overnight afforded 4.0 mg of the acetyl derivative **10**. Filtration of this material on a short pad of silica gel (eluent: *n*-hexane-EtOAc, 9:1), gave pure acetate **10** (2.8 mg, 45% over three steps) as a colorless oil. **10**: ${\left[\alpha \right]}_{D}^{20}$ = +18.2 (c = 0.19, CHCl_3_). IR (neat) λ_max_ 1746, 1227 cm^−1^. ^1^H-NMR: (700 MHz, CDCl_3_): δ 4.60 (ddd, *J* = 8.3, 6.7, 1.8, 1H), 4.43 (dd, *J* = 11.3, 6.7, 1H), 4.40 (dd, *J* = 11.3, 8.3, 1H), 4.10 (ddd, *J* = 12.1, 9.7, 4.5, 1H), 3.75 (ddd, *J* = 12.0, 10.0, 4.3, 1H), 3.51 (dd, *J* = 9.7, 1.8, 1H), 3.43 (ddd, *J* = 10.0, 8.5, 2.5, 1H), 3.01 (ddd, *J* = 12.1, 4.5, 4.3, 1H), 2.46 (ddd, *J* = 12.9, 12.1, 12.0, 1H), 2.11 (s, 3H, acetate), 2.03 (ddq, 14.5, 7.4, 2.5 1H), 1.55 (ddq, *J* = 14.5, 8.5, 7.4, 1H), 0.96 (t, *J* = 7.4, 3H). ^13^C-NMR: (176 MHz, CDCl_3_): δ 170.2, 83.7, 79.3, 64.7, 49.9, 47.5, 46.6, 45.3, 25.8, 20.7, 9.4. HRESIMS *m*/*z*: calcd for C_11_H_17_^79^Br_3_NaO_3_ 456.8626 [M + Na]^+^, found: 456.8629.

### 3.10. Lactone 11

PCC (10 mol%, 150 μL of a 0.01 M stock solution in acetonitrile) was added to a vigorously stirred suspension of H_5_IO_6_ (15 eq., 0.022 mmol, 49.8 mg) in acetonitrile (100 μL) at room temperature. After 5 min., compound **4** (9.0 mg, 0.015 mmol) dissolved in acetonitrile (100 μL + 2 × 100 μL rinse) was added to give a yellow cloudy mixture. After 16 h TLC analysis (*n*-hexane-EtOAc, 8:2) revealed the presence of a product at Rf = 0.3. No further progress of the process was observed after 2 h. Therefore, freshly prepared CCP (12 mol%, 180 μL of a 0.01 M stock solution in acetonitrile) was added to the reaction mixture. After 15 min., TLC analysis revealed the further progress of the process, but successive TLC analyses indicated no further consumption of the starting material. Two successive additions of the same amounts of CCP were required to drive the process to completion (overall further 2 h). Then, EtOH (1 mL) was added and the mixture was taken to dryness. Filtration of this material on a short pad of silica gel (eluent: CHCl_3_-MeOH, 95:5) followed by HPLC purification (*n*-hexane-EtOAc, 7:3) gave pure acetate **11** (4.1 mg, 55%) as a colourless oil. ${\left[\alpha \right]}_{D}^{20}$ = +27.7 (c = 0.13, CHCl_3_). IR (neat) λ_max_ 1794, 1745, 1225 cm^−1^. ^1^H-NMR: (700 MHz, CDCl_3_): δ 5.46 (1H, dd, *J* = 5.1, 3.3), 4.85 (1H, dd, *J* = 9.8, 3.3,), 4.75 (1H, dd, *J* = 9.8, 1.7), 4.19 (1H, ddd, *J* = 12.1, 9.5, 4.6), 3.75 (1H, ddd, *J* = 12.0, 10.5, 4.3), 3.42 (1H, ddd, *J* = 10.5, 8.4, 2.5), 3.26 (1H, dd, *J* = 9.5, 1.7), 3.01 (1H, ddd, *J* = 12.9, 4.6, 4.3), 2.95 (1H, dd, *J* = 18.1, 5.1), 2.69 (1H, d, *J* = 18.1), 2.41 (1H, ddd, *J* = 12.9, 12.1, 12.0), 2.17 (3H, s, acetate), 2.04 (1H, m), 1.59 (1H, m), 0.96 (3H, t, *J* = 7.4). ^13^C-NMR: (176 MHz, CDCl_3_): δ 171.9, 169.8, 83.7, 83.0, 79.3, 69.5, 49.8, 46.7, 46.1, 44.9, 37.9, 25.7, 21.0, 9.5. HRESIMS *m*/*z*: calcd for C_14_H_19_^79^Br_3_NaO_5_ 526.8680 [M + Na]^+^, found: 525.8671.

### 3.11. Mycalin B 2

KOH (excess) was added to a stirred solution of **1** (2.5 mg, 0.044 mmol) in MeOH (0.5 mL). After 10 min AcOH was added up to neutrality. The mixture was taken to dryness, the residue was partitioned between water and EtOAc and the organic phase was dried and evaporated. HPLC separation of the crude (eluent: *n*-hexane-EtOAc, 8:2) gave pure **2** (1.5 mg, 70%) as a colourless oil. **2**: For the ${\left[\alpha \right]}_{D}^{20}$, IR and MS (mass spectrometry) data see refs. 5 and 8. ^1^H-NMR: (400 MHz, CDCl_3_): δ 4.60 (1H, bd, *J* = 2.4), 4.55 (1H, bd, *J* = 2.1), 4.51 (1H, bd, *J* = 2.2), 4.39 (1H, dd, *J* = 8.9, 2.0), 4.24 (1H, bd, *J* = 8.9), 4.13 (1H, ddd, *J* = 12.7, 9.6, 4.4), 3.73 (1H, ddd, *J* = 12.2, 9.9, 4.5), 3.58 (1H, dd, *J* = 9.5, 2.0), 3.38 (1H, ddd, *J* = 9.5, 9.5, 2.3), 3.00 (1H, ddd, *J* = 12.8, 4.3, 4.3), 2.59 (1H, d, *J* = 2.1), 2.44 (1H, ddd, *J* = 12.3, 12.3, 12.3), 2.38 (1H, dd, *J* = 10.6, 2.9), 2.10 (1H, dd, *J* = 10.6, 2.3), 2.04 (1H, m), 1.52 (1H, m), 0.92 (3H, t, *J* = 7.3). ^13^C-NMR: (100 MHz, CDCl_3_): δ 86.0, 83.9, 81.0, 80.7, 79.5, 76.5, 76.1, 73.6, 56.1, 47.5, 46.5, 45.1, 36.6, 25.8, 9.8.

### 3.12. Cell Lines and Culture Conditions

The human cervical adenocarcinoma (HeLa), human breast adenocarcinoma (MCF-7) and human malignant melanoma (A375) cell lines were from ATCC (U.S.). The normal human dermal fibroblasts (HDF) were kindly provided by Dr. Annalisa Tito (Arterra, Biosciences). The cells were grown in DMEM (Dulbecco’s Modified Eagle Medium) supplemented with 10% fetal bovine serum (FBS), 1% glutamine, 100 U/mL penicillin and 100 µg/mL streptomycin (Euroclone, Milan, Italy) and maintained in humidified air containing 5% CO_2_, at 37 °C.

### 3.13. Cell Proliferation Assay

The cells were plated at a density of 1200 cells/well for A375, 1000 cells/well for HeLa and 2000 cells/well for HDF and MCF-7, in 96-well microplates (Corning). After 24 h incubation, the cells were treated with different concentrations of each of the compounds **1**, **2** and **4–11**, previously solubilized in DMSO (vehicle). The cell proliferation was determined after 48 h of treatment by using MTT (Sigma Aldrich, Milan, Italy) as previously reported. The plates were then analyzed by using a microplate reader (Enspire, Perkin Elmer, Cambridge, MA, USA) at 570 nm. The mean value ± SE of the adherent cells for each treatment was expressed as the relative percentage of the cell number with respect to the cells treated with the vehicle (control). Statistical differences were determined by the Student’s test, paired, two-sided. All the experiments were performed at least in triplicate and repeated at least 3 times; a *p* value less than 0.05 was considered to be significative. The IC_50_ values were obtained by the Prism 6.01 software (GraphPad San Diego, CA, U.S.) by extrapolating them from the dose–response curves data [18].

### 3.14. Apoptosis Experiments

The apoptosis assays were performed on the A375 cells seeded at a density of 1x10^5^ cells/well in 6 well plates and incubated with compounds **1**, **4**, **5**, **and 11** at 37 °C and at 10 µM concentration for 48 h. The apoptosis was then analysed by staining with annexin V/FITC and propidium iodine (PI) (eBioscience). Briefly, after incubation, the cells were detached with accutase solution (eBioscience), harvested and washed with cold PBS [19]. Subsequently, the cells were treated following the manufacturer’s instructions. The percentage of cell undergoing apoptosis or necrosis was quantified using a flow cytometer (Becton Dickinson, San Diego, CA, U.S.) equipped with the Cell Quest software version 3.5.1

## 4. Conclusions

In conclusion, in this study we have shown that mycalin A, a polybrominated C_15_ acetogenin of marine origin, is a strongly cytotoxic and pro-apoptotic substance on A375 tumor cells. The synthesis of some simplified analogues of this substance has been accomplished. It has been observed that the left-hand portion of mycalin A is essential for the biological activity since its perturbation, or its absence, strongly affects the antiproliferative activity, as the reduced cytotoxicity of mycalin B and the THP derivatives show. A simplified C4-lactone analogue of the mycalin A acetate has displayed a high cytotoxicity towards A375 malignant cells mediated by an apoptotic process, a result which appears very important considering that the tested A375 cells derive from a tumor with a negative prognosis. In addition, this substance has shown a reasonable selectivity with respect to healthy cells. A less pronounced activity on tumor cells is displayed by the 4-propyl-analogue of mycalin A acetate that did not show any selectivity. Studies are in progress to synthesize further simplified analogues of mycalin A and of its lactone derivative and to identify their biological target(s). Due to its high anti-proliferative and apoptotic activity, the acetate derivative of mycalin A (**4**), will also be the subject of further structural modifications with the aim of increasing its selectivity of action.

## Figures and Tables

**Table 1:** IC_50_ values of mycalin A (**1**) obtained on the responsive treated cells, after 48h incubation.

Cell Line	IC_50_ (µM)
A375	1.1 ± 0.29
HeLa	3.9 ± 1.88
HDF	0.32 ± 0.22

**Table 2:** Summary of the synthesized derivatives of mycalin A.

**Mycalin A (1)**	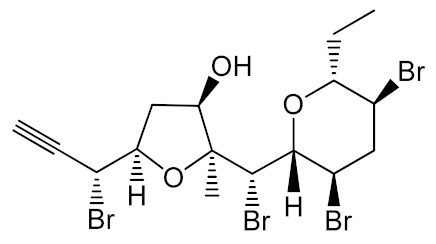	**Mycalin B (2)**	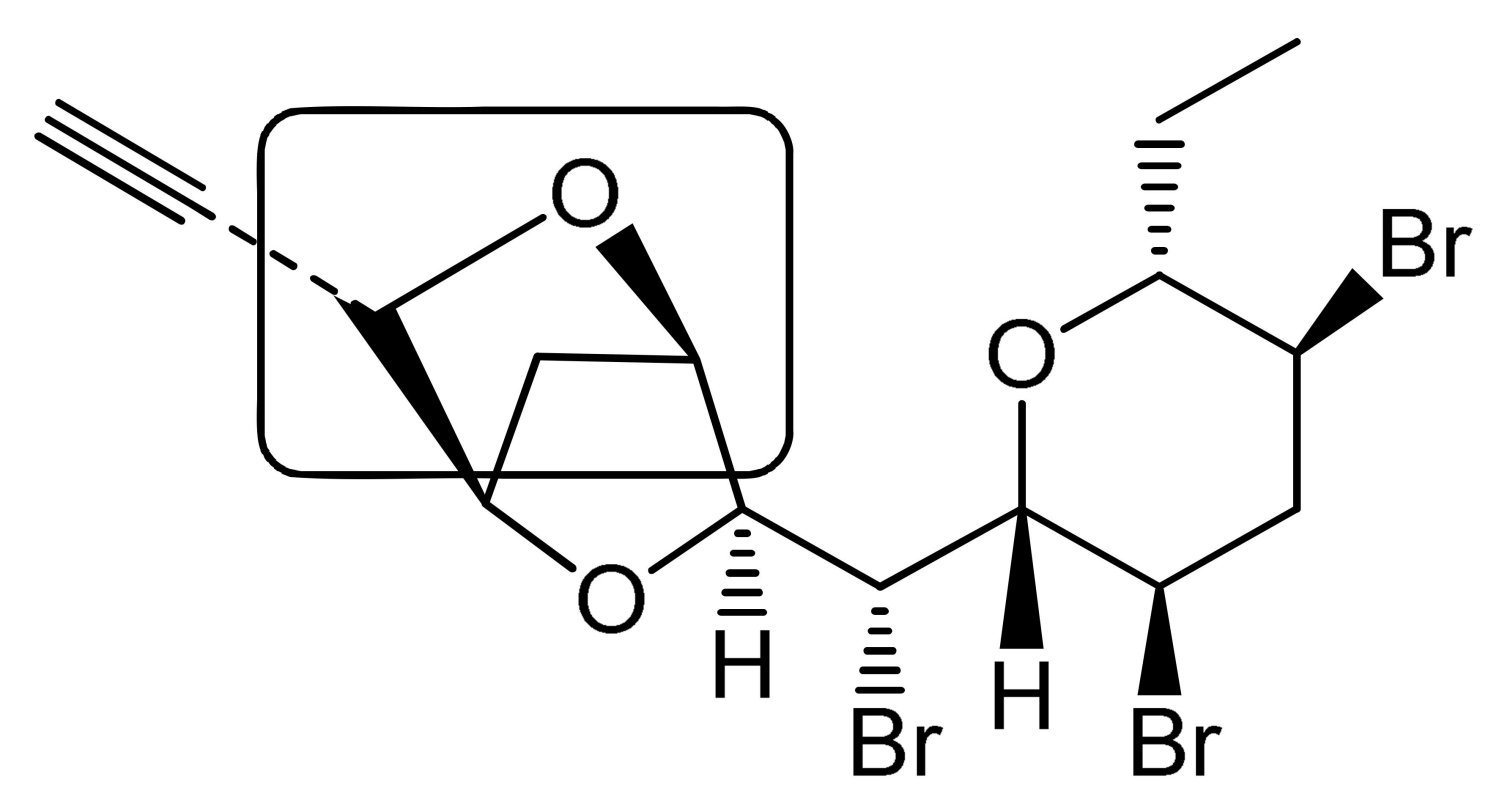
**Mycalin A acetate (4)**	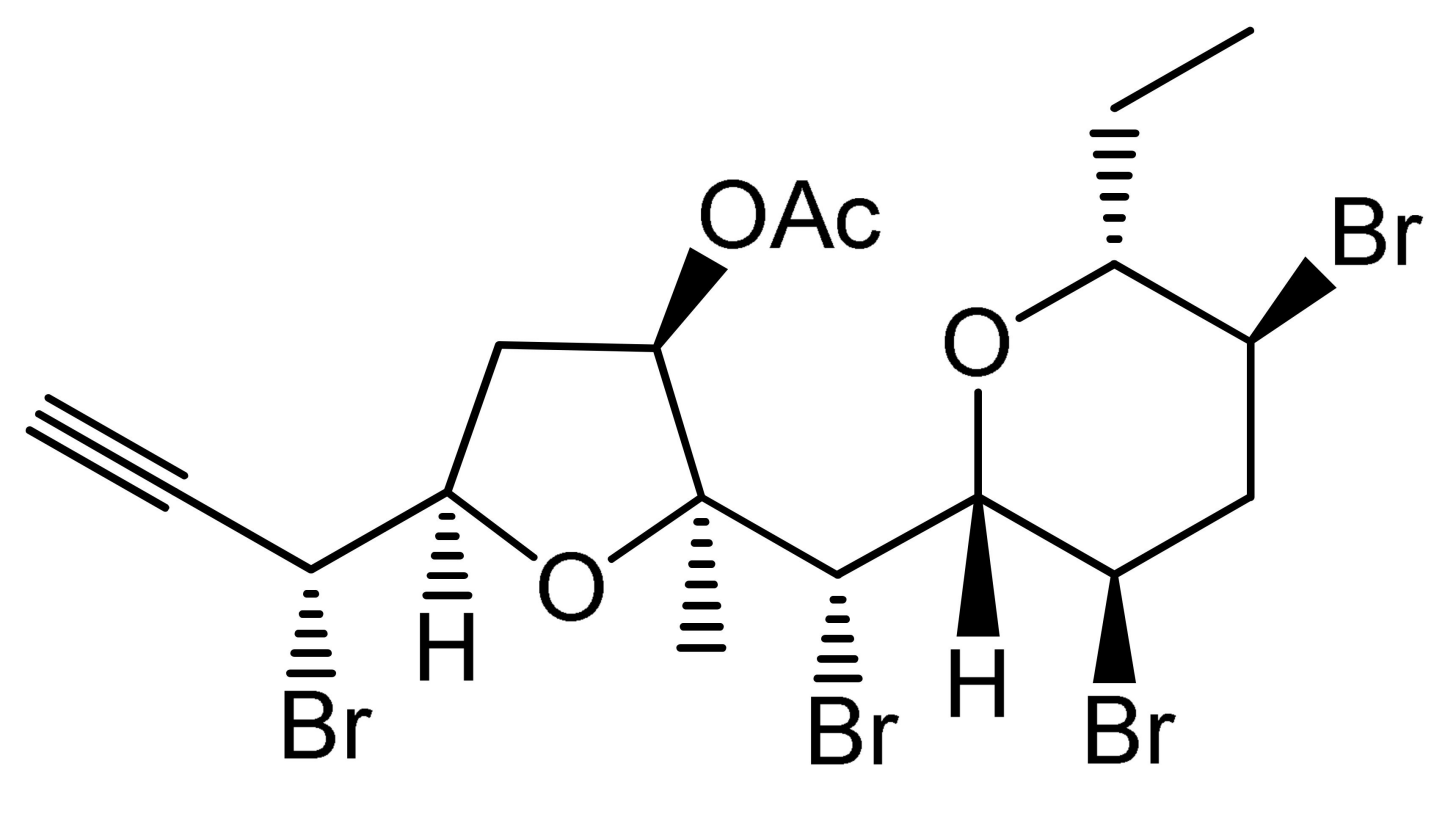	**5**	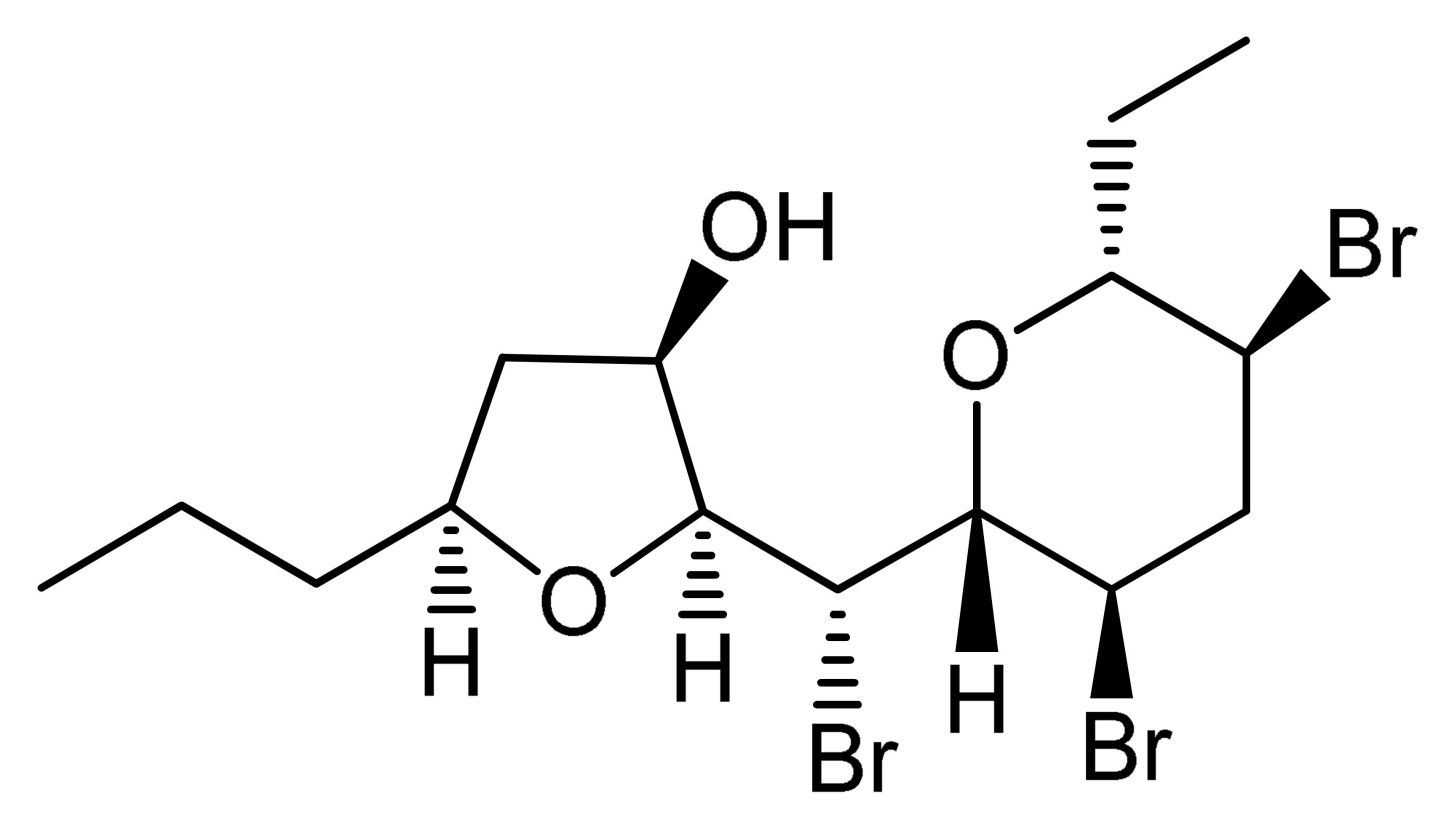
**6**	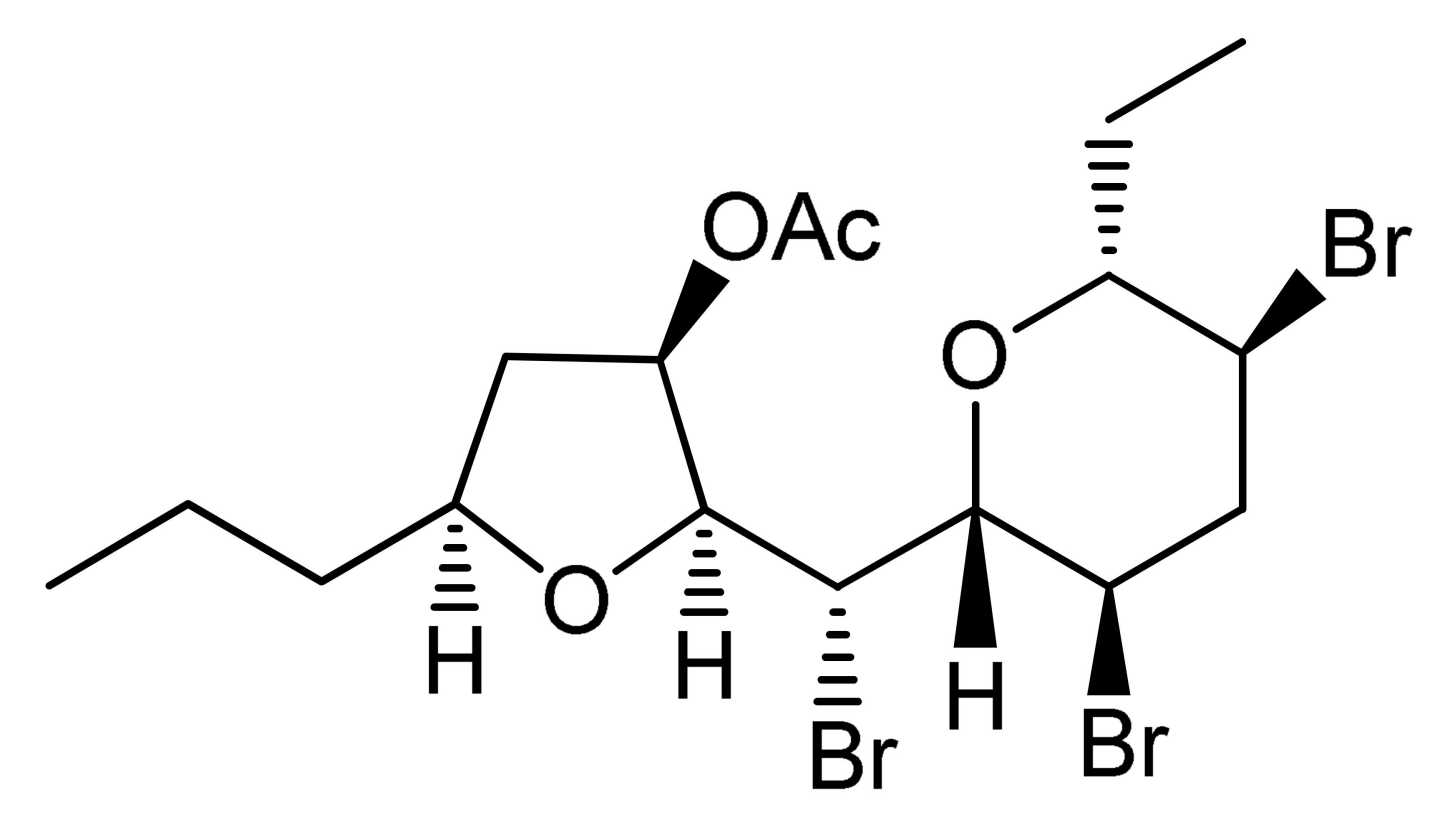	**7**	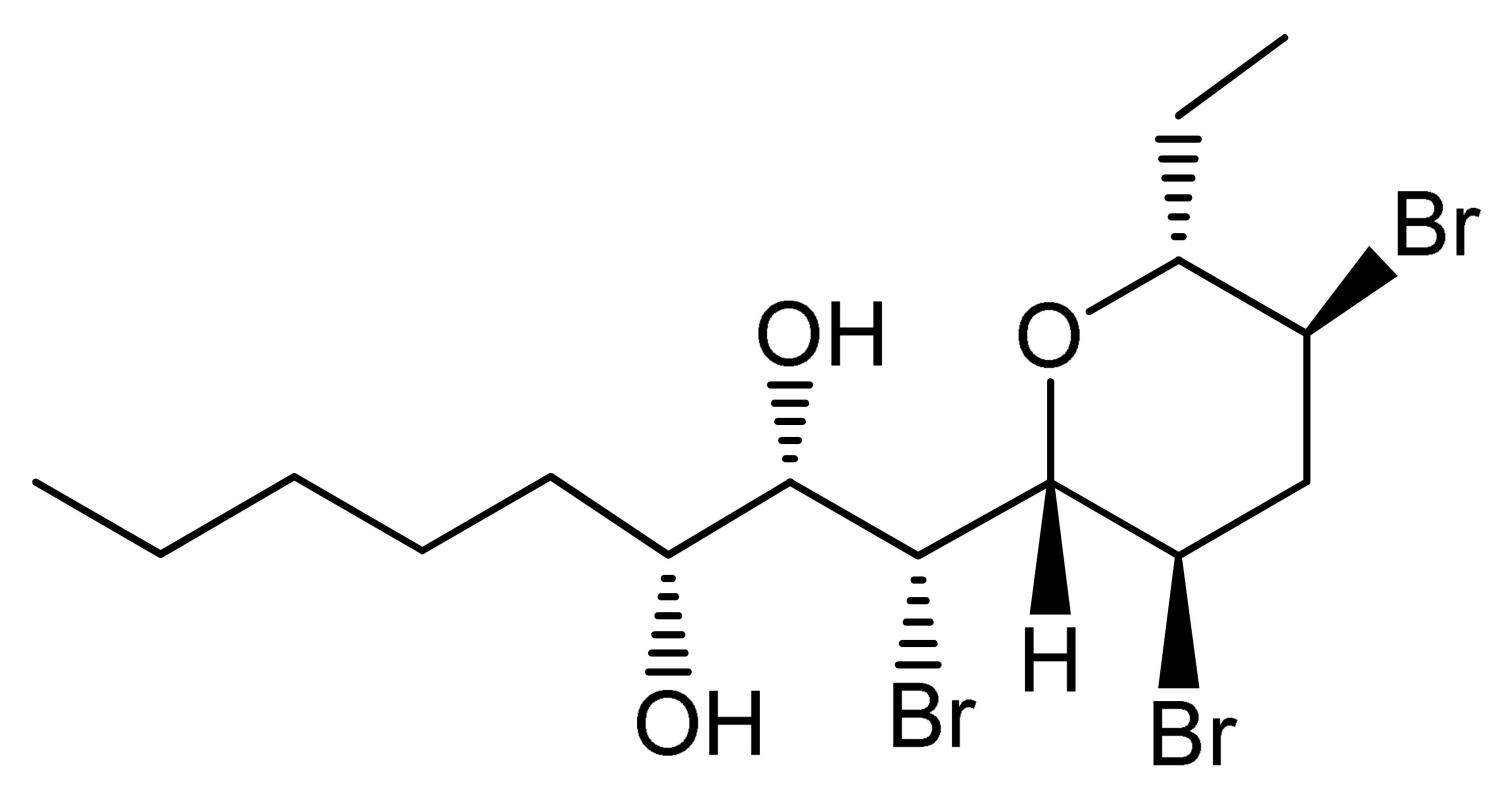
**8**	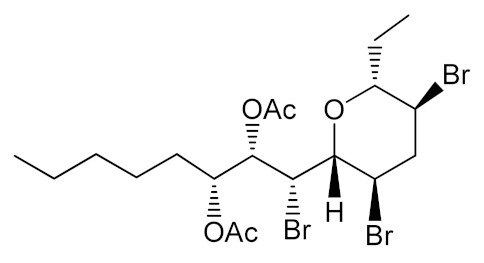	**9**	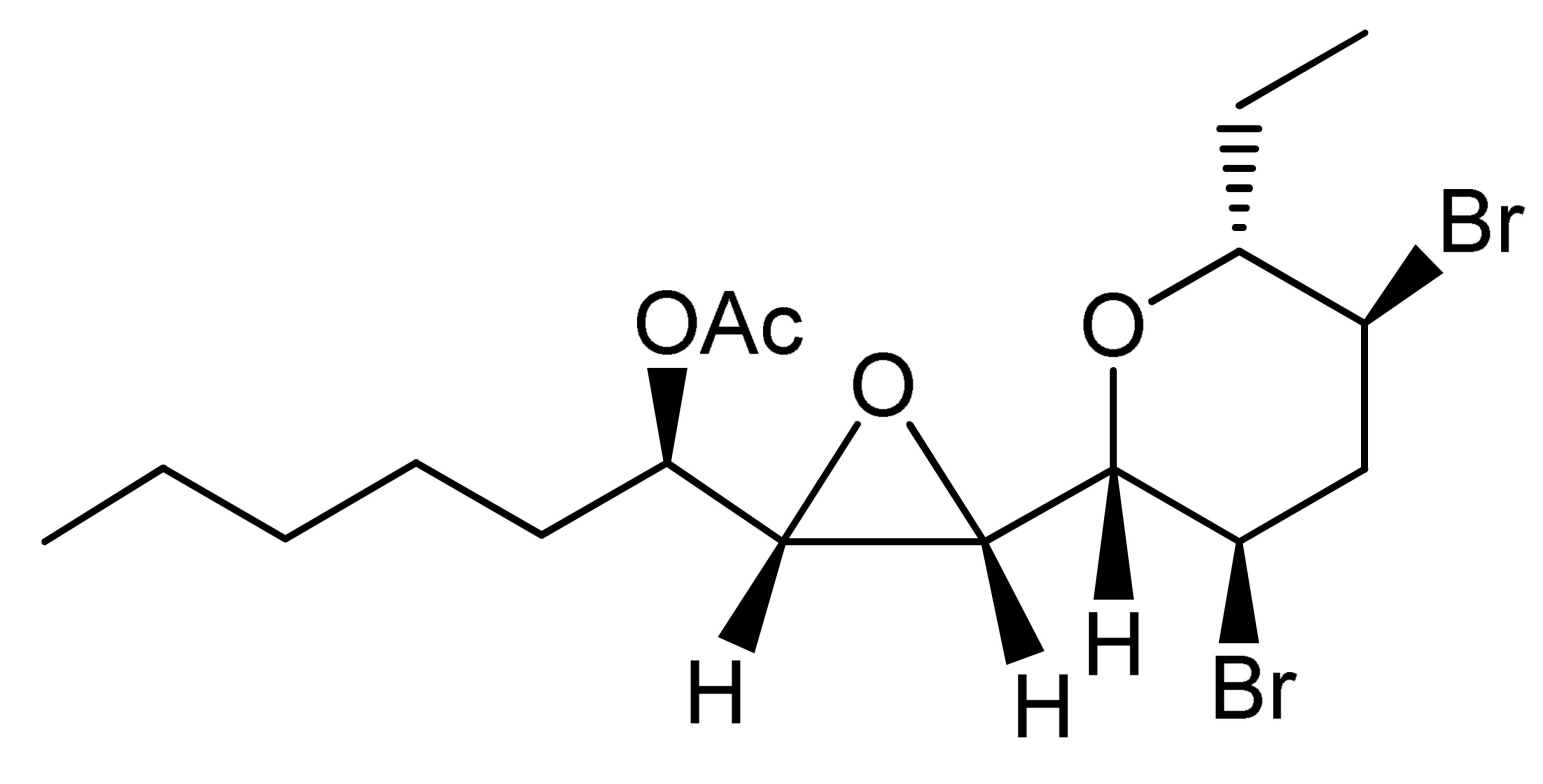
**10**	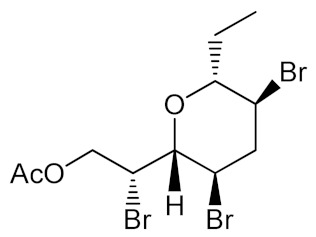	**11**	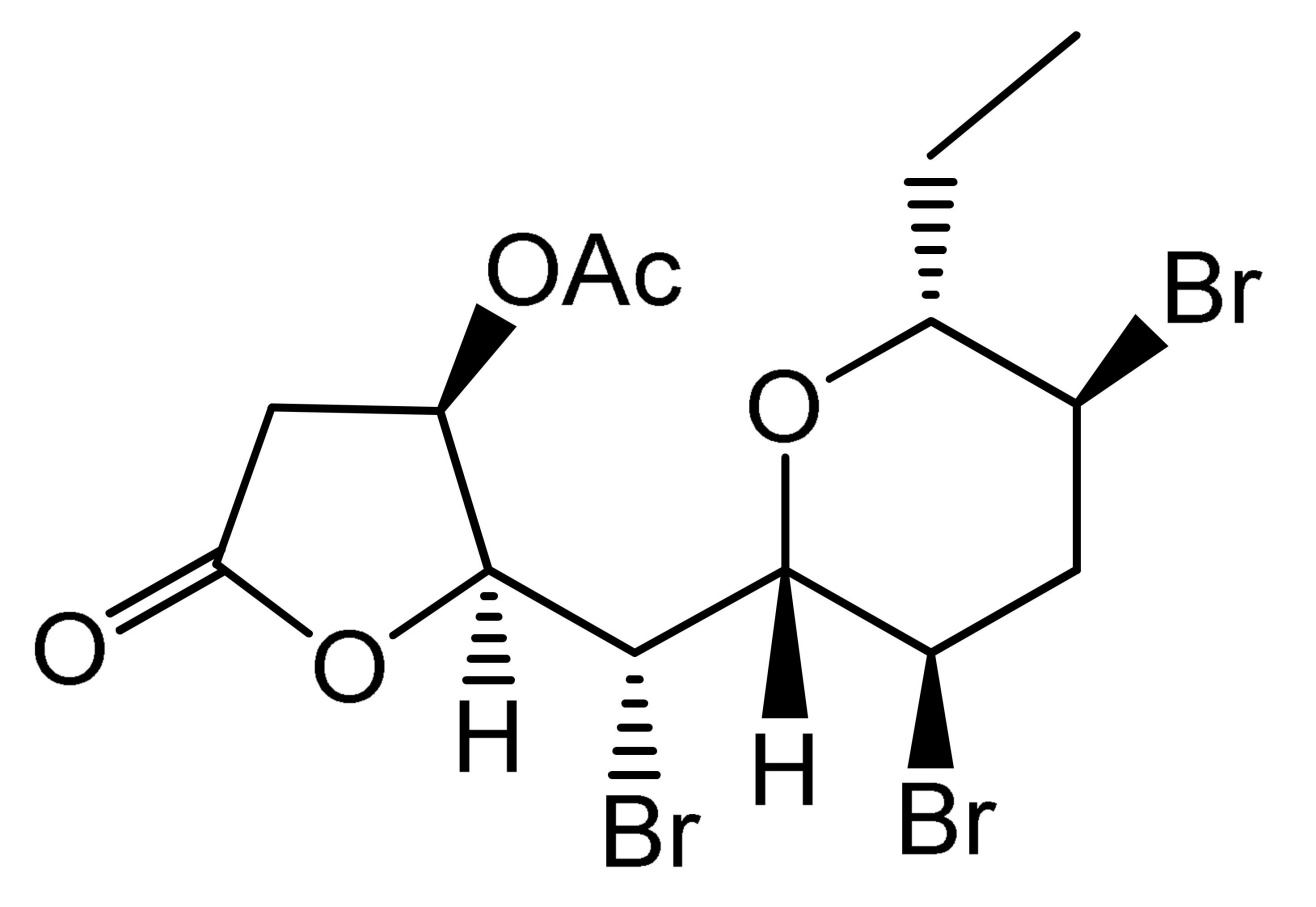

**Table 3:** IC_50_ values obtained from the dose–response curves of acetate **4**, 4-propyl-derivative **5** and lactone **11** on all the tested cell lines, after 48h incubation.

Cell Line	4	5	11
A375	0.62 ± 0.20	14.11 ± 3. 5	1.1 ± 0.22
HeLa	0.92 ± 0.23	18.5 ± 5.0	6.7 ± 2.3
HDF	0.11 ± 0.004	23.2 ± 7.8	11.2 ± 6.3

## Supplementary Materials

The following are available online at https://www.mdpi.com/1660-3397/18/8/402/s1, Figure S1: ^1^H NMR spectrum of compound **1** (CDCl_3_, 400 MHz), Figure S2: ^13^C NMR spectrum of compound **1** (CDCl_3_, 100 MHz), Figure S3: ^1^H NMR spectrum of compound **2** (CDCl_3_, 400 MHz), Figure S4: ^13^C NMR spectrum of compound **2** (CDCl_3_, 100 MHz), Figure S5: ^1^H NMR spectrum of compound **4** (CDCl_3_, 400 MHz), Figure S6: ^13^C NMR spectrum of compound **4** (CDCl_3_, 100 MHz), Figure S7: ^1^H NMR spectrum of compound **5** (CDCl_3_, 400 MHz), Figure S8: ^13^C NMR spectrum of compound **5** (CDCl_3_, 100 MHz), Figure S9: ^1^H NMR spectrum of compound **6** (CDCl_3_, 400 MHz)., Figure S10: ^13^C NMR spectrum of compound **6** (CDCl_3_, 100 MHz), Figure S11: 2D COSY spectrum of compound **6** (CDCl_3_, 700 MHz), Figure S12: 2D NOESY spectrum of compound **6** (CDCl_3_, 700 MHz), Figure S13: 2D HSQC spectrum of compound **6** (CDCl_3_, 700 MHz), Figure S14: 2D HMBC spectrum of compound **6** (CDCl_3_, 700 MHz), Figure S15: ^1^H NMR spectrum of compound **7** (CDCl_3_, 400 MHz), Figure S16: ^13^C NMR spectrum of compound **7** (CDCl_3_, 100 MHz), Figure S17: ^1^H NMR spectrum of compound **8** (CDCl_3_, 400 MHz), Figure S18: ^13^C NMR spectrum of compound **8** (CDCl_3_, 100 MHz), Figure S19: 2D COSY spectrum of compound **8** (CDCl_3_, 700 MHz), Figure S20: 2D NOESY spectrum of compound **8** (CDCl_3_, 700 MHz), Figure S21: ^1^H NMR spectrum of compound **9** (CDCl_3_, 400 MHz), Figure S22: ^13^C NMR spectrum of compound **9** (CDCl_3_, 100 MHz), Figure S23: 2D COSY spectrum of compound **9** (CDCl_3_, 700 MHz), Figure S24: 2D NOESY spectrum of compound **9** (CDCl_3_, 700 MHz), Figure S25: 2D HSQC spectrum of compound **9** (CDCl_3_, 700 MHz), Figure S26: 2D HMBC spectrum of compound **9** (CDCl_3_, 700 MHz), Figure S27: ^1^H NMR spectrum of compound **10** (CDCl_3_, 400 MHz), Figure S28: ^13^C NMR spectrum of compound **10** (CDCl_3_, 100 MHz), Figure S29: 2D COSY spectrum of compound **10** (CDCl_3_, 400 MHz), Figure S30: 2D NOESY spectrum of compound **10** (CDCl_3_, 400 MHz), Figure S31: 2D HSQC spectrum of compound **10** (CDCl_3_, 700 MHz), Figure S32: 2D HMBC spectrum of compound **10** (CDCl_3_, 700 MHz), Figure S33: ^1^H NMR spectrum of compound **11** (CDCl_3_, 400 MHz), Figure S34: ^13^C NMR spectrum of compound **11** (CDCl_3_, 100 MHz), Figure S35: 2D COSY spectrum of compound **11** (CDCl_3_, 700 MHz), Figure S36: 2D NOESY spectrum of compound **11** (CDCl_3_, 700 MHz), Figure S37: 2D HSQC spectrum of compound **11** (CDCl_3_, 700 MHz), Figure S38: 2D HMBC spectrum of compound **11** (CDCl_3_, 700 MHz), Table S1: ^1^H and ^13^C chemical shift data for compound **6** (700 MHz, CDCl_3_), Table S2: ^1^H and ^13^C chemical shift data for compound **8** (700 MHz, CDCl_3_), Table S3: ^1^H and ^13^C chemical shift data for compound **9** (700 MHz, CDCl_3_), Table S4: ^1^H and ^13^C chemical shift data for compound **10** (700 MHz, CDCl_3_), Table S5: ^1^H and ^13^C chemical shift data for compound **11** (700 MHz, CDCl_3_).

## Appendix A

† Since in the original manuscripts (see refs. 5 and 6) no common names were given to these compounds we propose here the common names mycalin A, B and C for compounds 1, 2 and 3, respectively.

‡ Comparison of the X-ray molecular structure and crystallographic data reported by Imre et al. for their compound 2 with those exhibited by mycalin A unambiguously shows that they are identical compounds. In particular, the molecular formula and the spatial group are the same, and the elemental cell parameters differ within 0.2%. However, their drawing incorrectly shows the relative stereochemistry of this compound and does not match the X-ray image they report. Similarly, the configuration of mycalin B (their compound 1) is incorrectly reported.

§ The configuration of mycalin A isolated from this alga is incorrectly reported. In particular, the configuration of the stereogenic centres C-3, C-9, C-10, C-12 and C-13 is inverted.
