# Supplementary material for: Antiproliferative Activity of Mycalin A and Its Analogues on Human Skin Melanoma and Human Cervical Cancer Cells
Source: Mar Drugs. 2020 Jul 29;18(8):402. doi: 10.3390/md18080402 (PMC7547386; doi:10.3390/md18080402)
Supplement: Supplementary file 1 [file marinedrugs-18-00402-s001.pdf]

marine drugs

# Supplementary Information

# Antiproliferative Activity of Mycalin A and Its Analogues on Human Skin Melanoma and Human Cervical Cancer Cells

**Domenica Capasso [1], Nicola Borbone [2], Monica Terracciano [2], Sonia Di Gaetano [3,*] and Vincenzo Piccialli [4,*]**

[1] CESTEV, University of Naples Federico II, 80145 Naples, Italy; domenica.capasso@unina.it
[2] Department of Pharmacy, University of Naples Federico II, 80131 Naples, Italy; nicola.borbone@unina.it (N.B.); monica.terracciano@unina.it (M.T.)
[3] Institute of Biostructures and Bioimaging, CNR, 80134 Naples, Italy
[4] Department of Chemical Sciences, University of Naples Federico II, 80126 Naples, Italy
[*] Correspondence: digaetan@unina.it (S.D); vinpicci@unina.it (V.P.)

## CONTENTS:

| |
|---|
| **Figure S1**: $^{1}$H NMR spectrum of compound **1** ($CDCl_3$, 400 MHz) |
| **Figure S2:** $^{13}$C NMR spectrum of compound **1** ($CDCl_3$, 100 MHz |
| **Figure S3:** $^{1}$H NMR spectrum of compound **2** ($CDCl_3$, 400 MHz) |
| **Figure S4:** $^{13}$C NMR spectrum of compound **2** ($CDCl_3$, 100 MHz |
| **Figure S5**: $^{1}$H NMR spectrum of compound **4** ($CDCl_3$, 400 MHz |
| **Figure S6:** $^{13}$C NMR spectrum of compound **4** ($CDCl_3$, 100 MHz) |
| **Figure S7:** $^{1}$H NMR spectrum of compound **5** ($CDCl_3$, 400 MHz) |
| **Figure S8:** $^{13}$C NMR spectrum of compound **5** ($CDCl_3$, 100 MHz) |
| **Figure S9:** $^{1}$H NMR spectrum of compound **6** ($CDCl_3$, 400 MHz). |
| **Figure S10:** $^{13}$C NMR spectrum of compound **6** ($CDCl_3$, 100 MHz) |
| **Figure S11:** 2D COSY spectrum of compound **6** ($CDCl_3$, 700 MHz) |
| **Figure S12:** 2D NOESY spectrum of compound **6** ($CDCl_3$, 700 MHz) |
| **Figure S13:** 2D HSQC spectrum of compound **6** ($CDCl_3$, 700 MHz) |

| |
|---|
| **Figure S14:** 2D HMBC spectrum of compound **6** ($CDCl_3$, 700 MHz) |
| **Figure S15:** $^1$H NMR spectrum of compound **7** ($CDCl_3$, 400 MHz) |
| **Figure S16:** $^{13}$C NMR spectrum of compound **7** ($CDCl_3$, 100 MHz) |
| **Figure S17:** $^1$H NMR spectrum of compound **8** ($CDCl_3$, 400 MHz) |
| **Figure S18:** $^{13}$C NMR spectrum of compound **8** ($CDCl_3$, 100 MHz) |
| **Figure S19:** 2D COSY spectrum of compound **8** ($CDCl_3$, 700 MHz) |
| **Figure S20:** 2D NOESY spectrum of compound **8** ($CDCl_3$, 700 MHz) |
| **Figure S21:** $^1$H NMR spectrum of compound **9** ($CDCl_3$, 400 MHz) |
| **Figure S22:** $^{13}$C NMR spectrum of compound **9** ($CDCl_3$, 100 MHz) |
| **Figure S23:** 2D COSY spectrum of compound **9** ($CDCl_3$, 700 MHz) |
| **Figure S24:** 2D NOESY spectrum of compound **9** ($CDCl_3$, 700 MHz) |
| **Figure S25:** 2D HSQC spectrum of compound **9** ($CDCl_3$, 700 MHz) |
| **Figure S26:** 2D HMBC spectrum of compound **9** ($CDCl_3$, 700 MHz) |
| **Figure S27:** $^1$H NMR spectrum of compound **10** ($CDCl_3$, 400 MHz). |
| **Figure S28:** $^{13}$C NMR spectrum of compound **10** ($CDCl_3$, 100 MHz) |
| **Figure S29:** 2D COSY spectrum of compound **10** ($CDCl_3$, 400 MHz) |
| **Figure S30:** 2D NOESY spectrum of compound **10** ($CDCl_3$, 400 MHz) |
| **Figure S31:** 2D HSQC spectrum of compound **10** ($CDCl_3$, 700 MHz) |
| **Figure S32:** 2D HMBC spectrum of compound **10** ($CDCl_3$, 700 MHz) |
| **Figure S33:** $^1$H NMR spectrum of compound **11** ($CDCl_3$, 400 MHz) |
| **Figure S34:** $^{13}$C NMR spectrum of compound **11** ($CDCl_3$, 100 MHz) |
| **Figure S35:** 2D COSY spectrum of compound **11** ($CDCl_3$, 700 MHz) |
| **Figure S36:** 2D NOESY spectrum of compound **11** ($CDCl_3$, 700 MHz) |
| **Figure S37:** 2D HSQC spectrum of compound **11** ($CDCl_3$, 700 MHz) |
| **Figure S38:** 2D HMBC spectrum of compound **11** ($CDCl_3$, 700 MHz) |
| **Table S1:** $^1$H and $^{13}$C chemical shift data for compound **6** (700 MHz, $CDCl_3$) |
| **Table S2:** $^1$H and $^{13}$C chemical shift data for compound **8** (700 MHz, $CDCl_3$) |

| |
|---|
| **Table S3:** $^{1}H$ and $^{13}C$ chemical shift data for compound **9** (700 MHz, $CDCl_3$) |
| **Table S4:** $^{1}H$ and $^{13}C$ chemical shift data for compound **10** (700 MHz, $CDCl_3$) |
| **Table S5:** $^{1}H$ and $^{13}C$ chemical shift data for compound **11** (700 MHz, $CDCl_3$) |

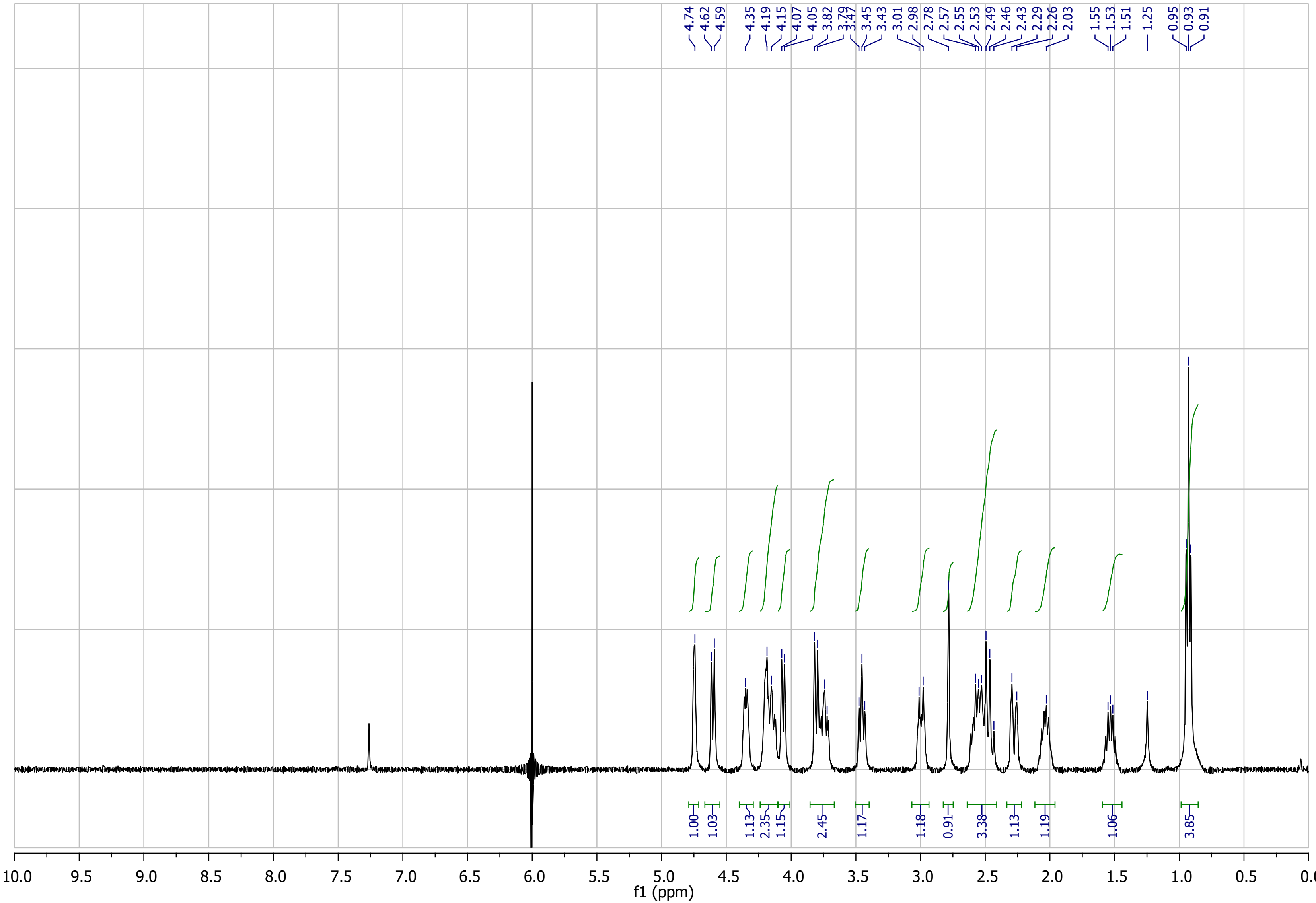


**Figure S1**: $^{1}$H NMR spectrum of compound **1** ($CDCl_3$, 400 MHz).

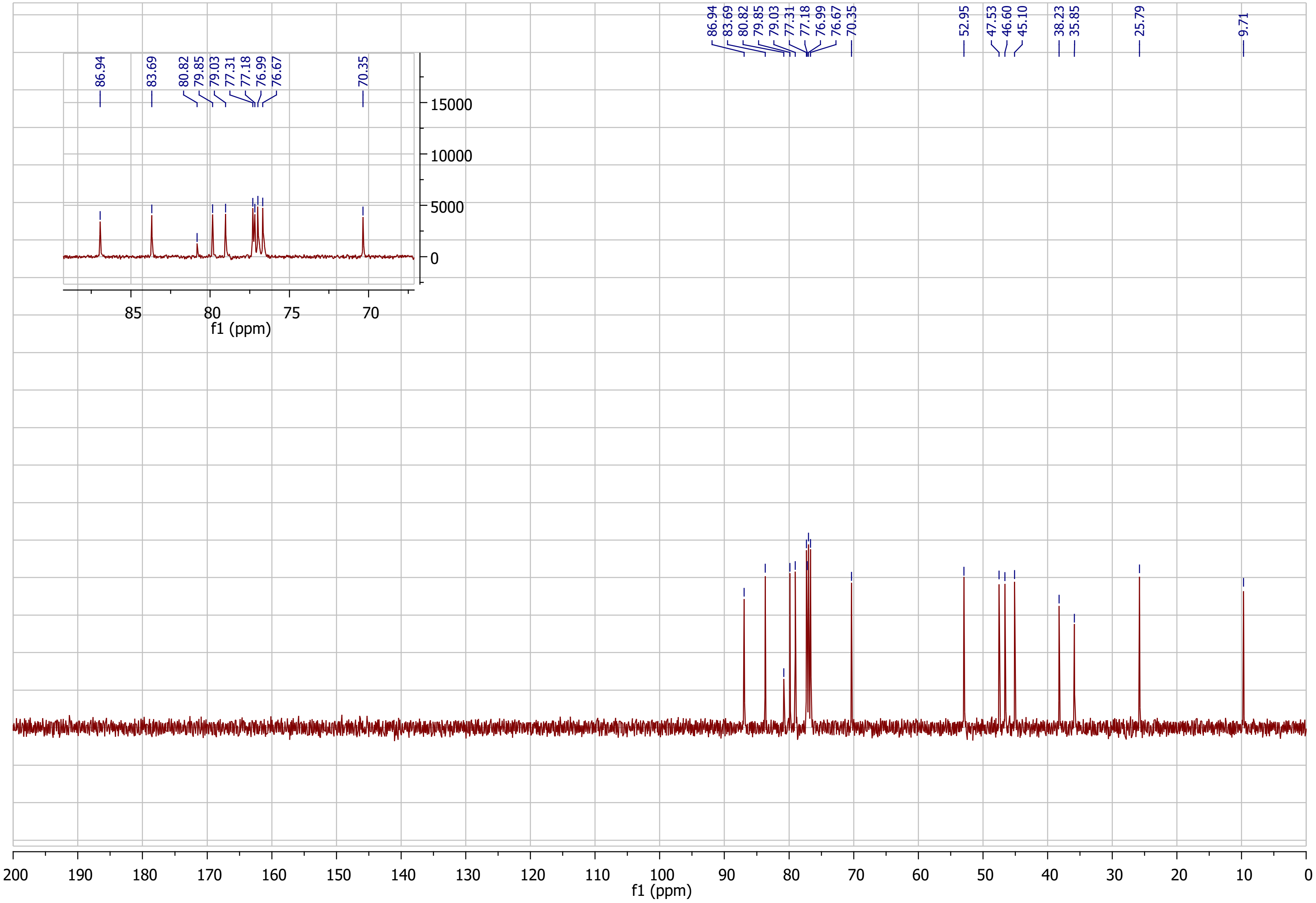


**Figure S2**: $^{13}C$ NMR spectrum of compound **1** ($CDCl_3$, 100 MHz).

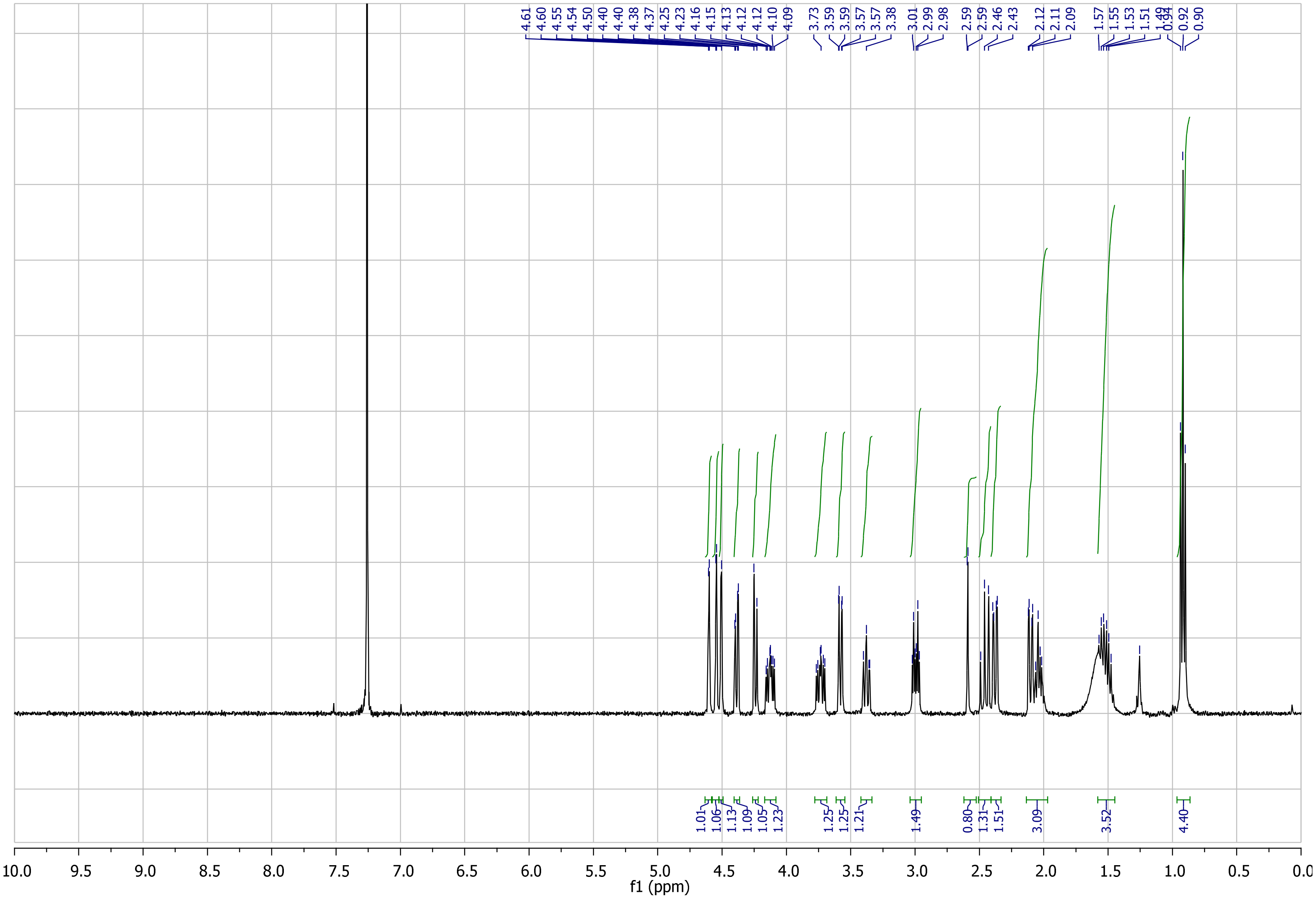

**Figure S3**: $^{1}$H NMR spectrum of compound **2** ($CDCl_3$, 400 MHz).

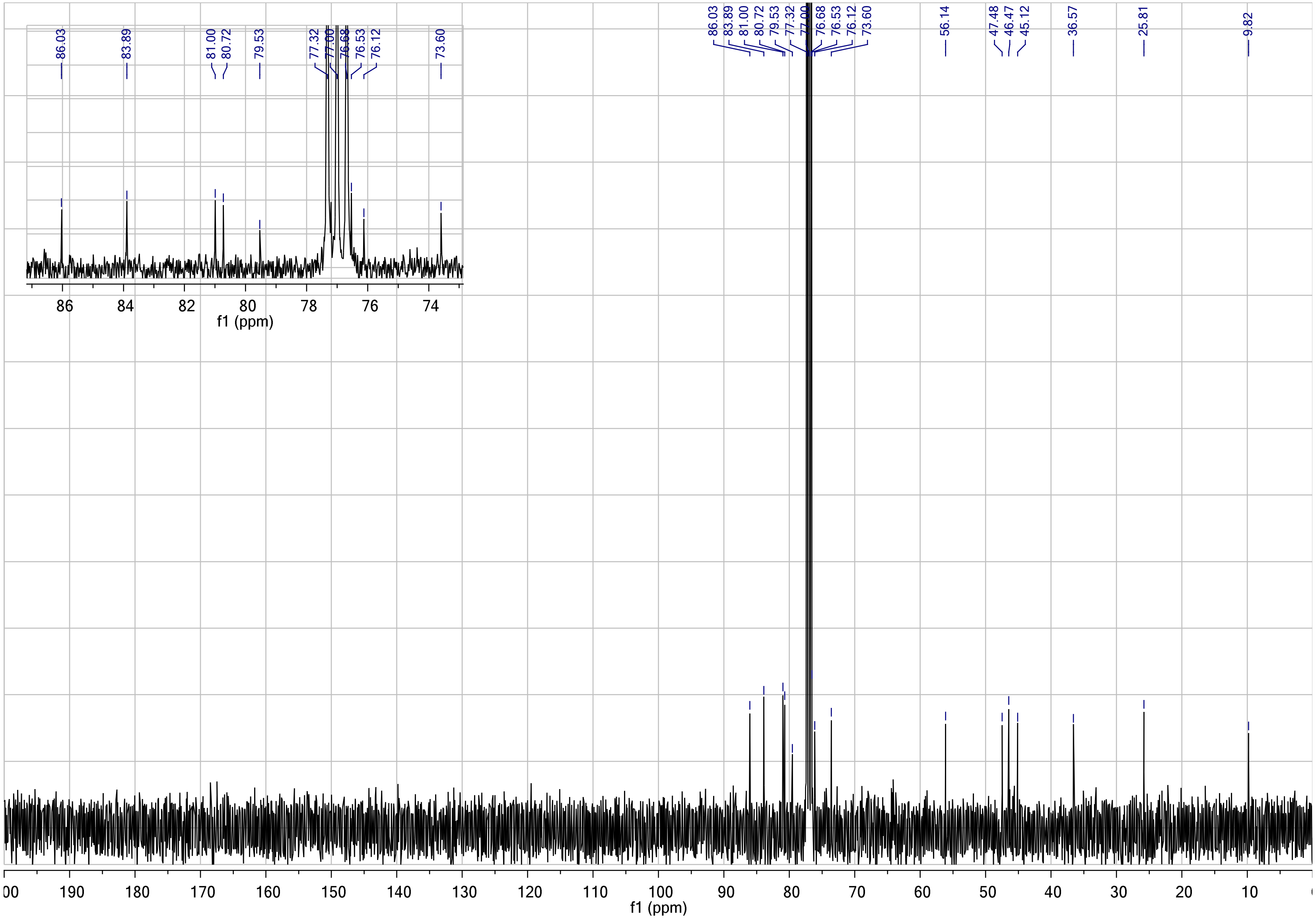


**Figure S4:** $^{13}$C NMR spectrum of compound **2** ($CDCl_3$, 100 MHz).

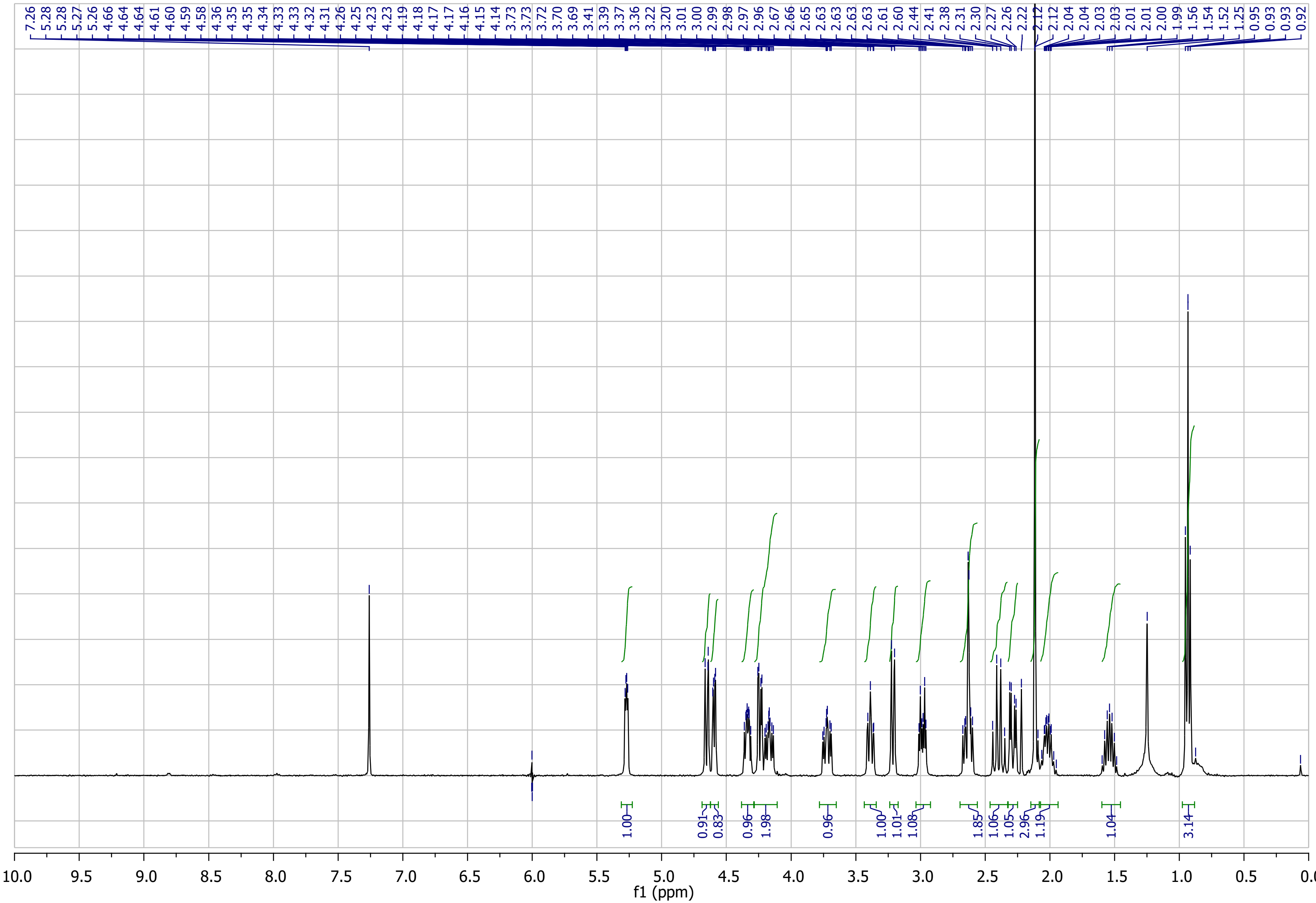

**Figure S5**: $^{1}$H NMR spectrum of compound **4** ($CDCl_3$, 400 MHz).

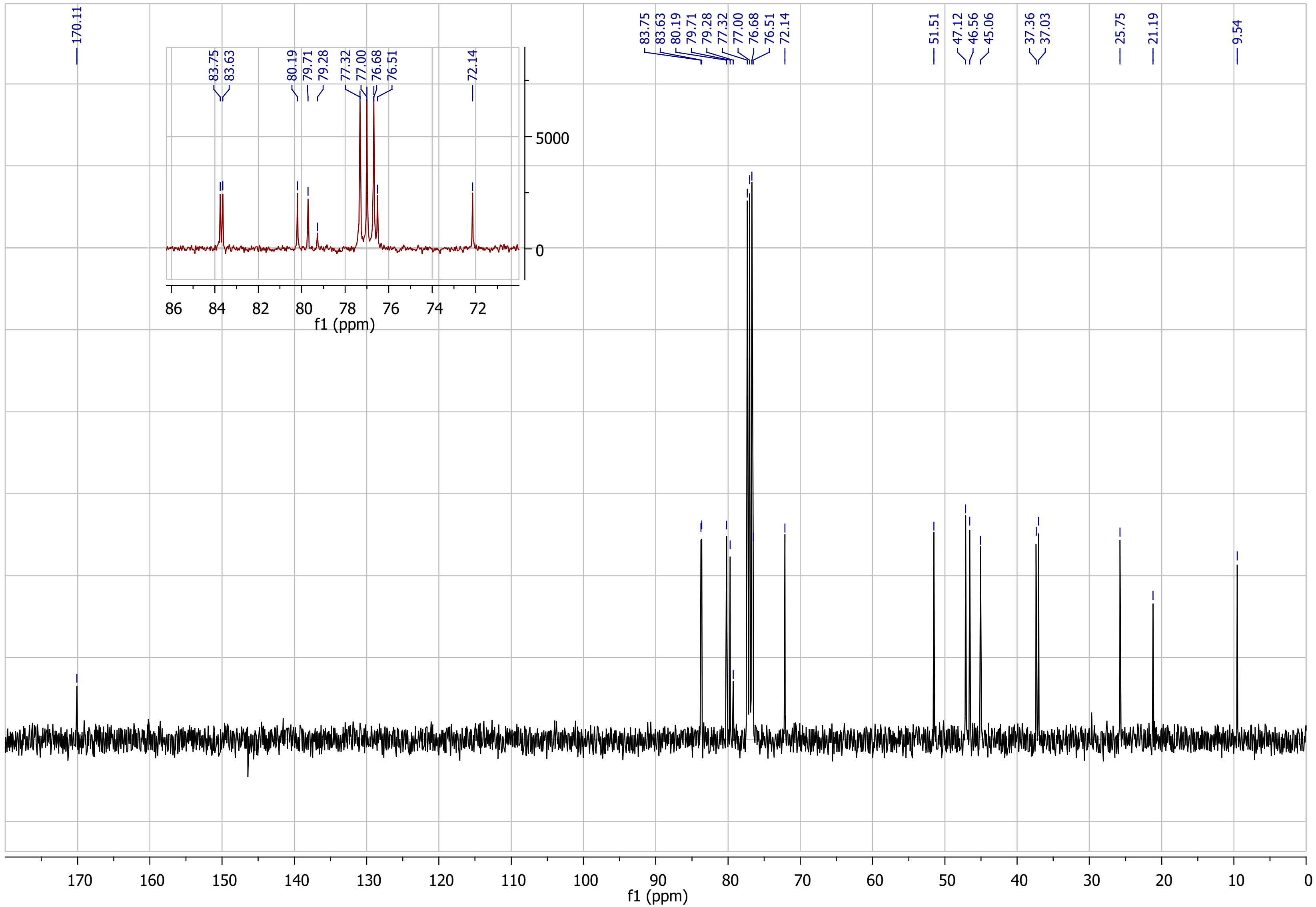


**Figure S6:** $^{13}C$ NMR spectrum of compound **4** ($CDCl_3$, 100 MHz).

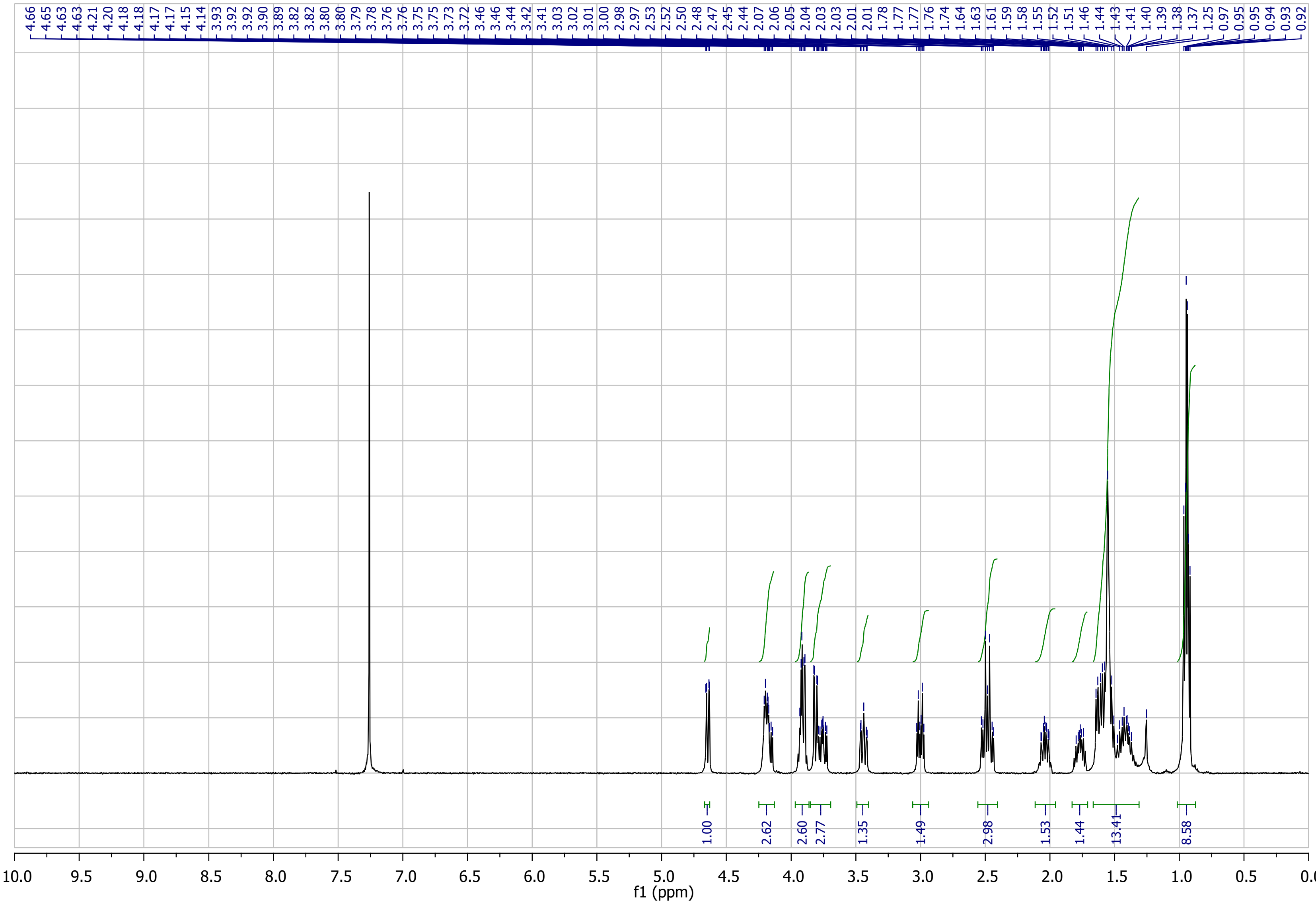

**Figure S7:** $^1$H NMR spectrum of compound **5** ($CDCl_3$, 400 MHz).

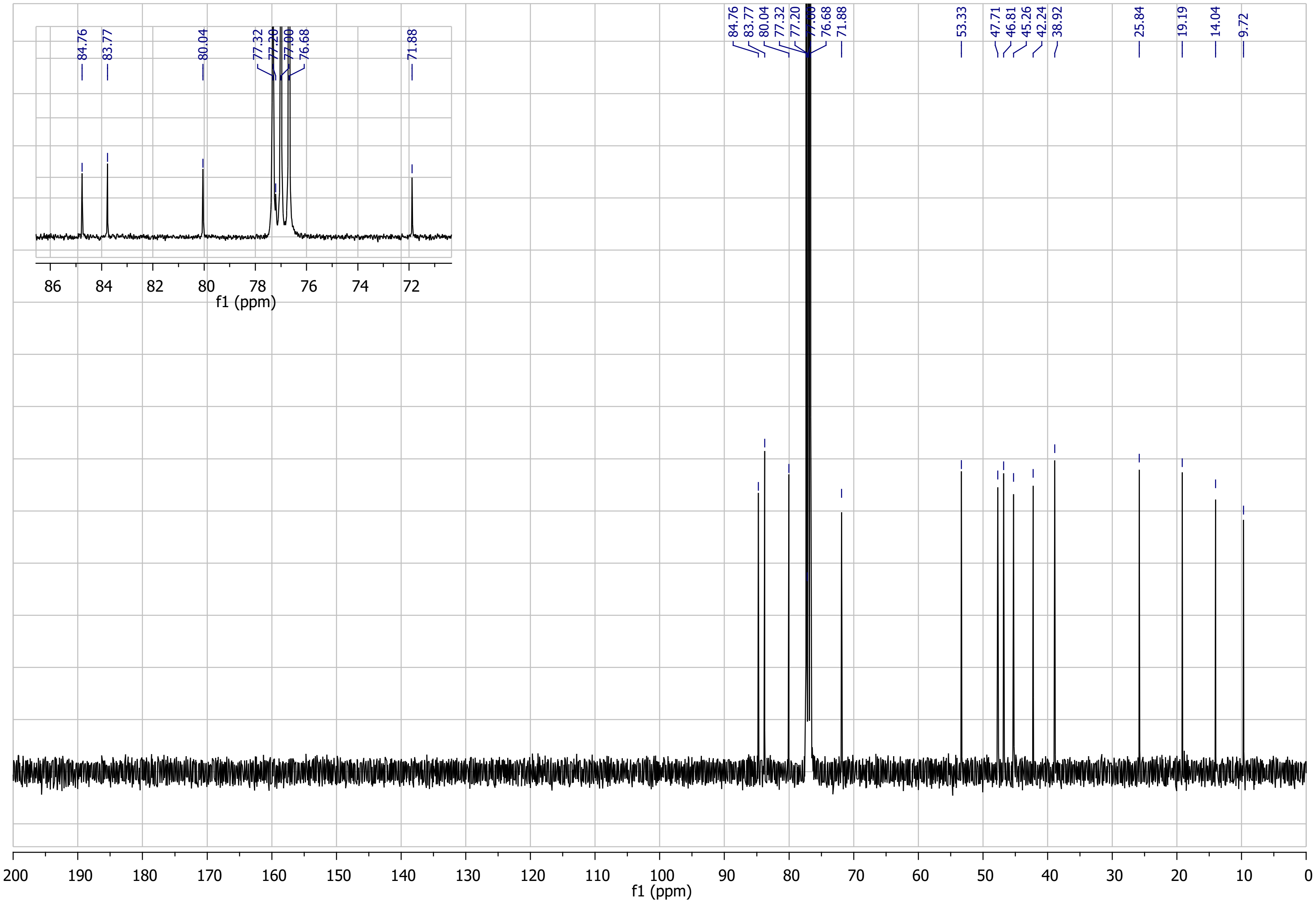


**Figure 8.** $^{13}$C NMR spectrum of compound **5** ($CDCl_3$, 100 MHz).

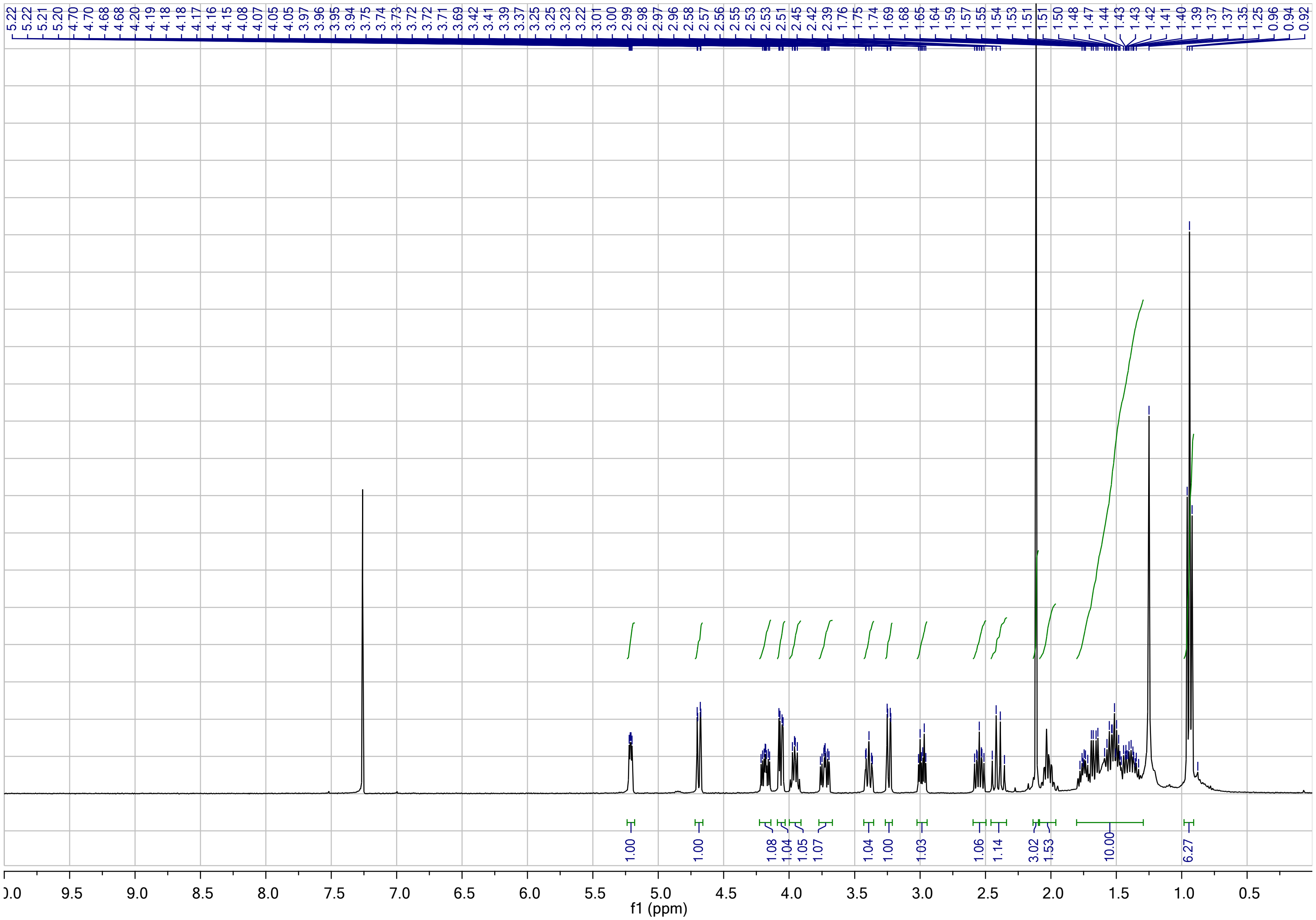


**Figure S9:** $^{1}H$ NMR spectrum of compound **6** ($CDCl_3$, 400 MHz).

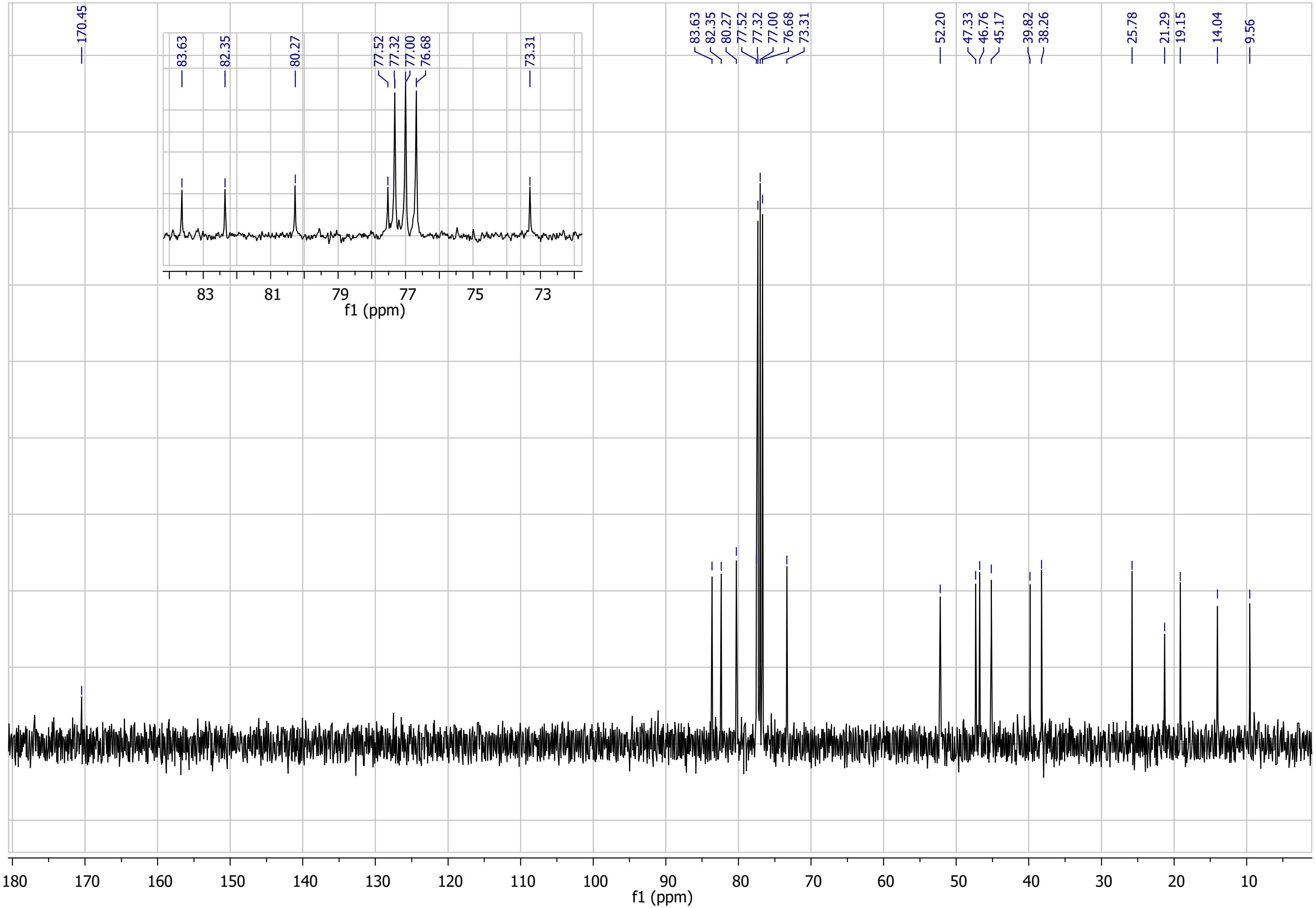


**Figure S10:** $^{13}$C NMR spectrum of compound **6** ($CDCl_3$, 100 MHz).

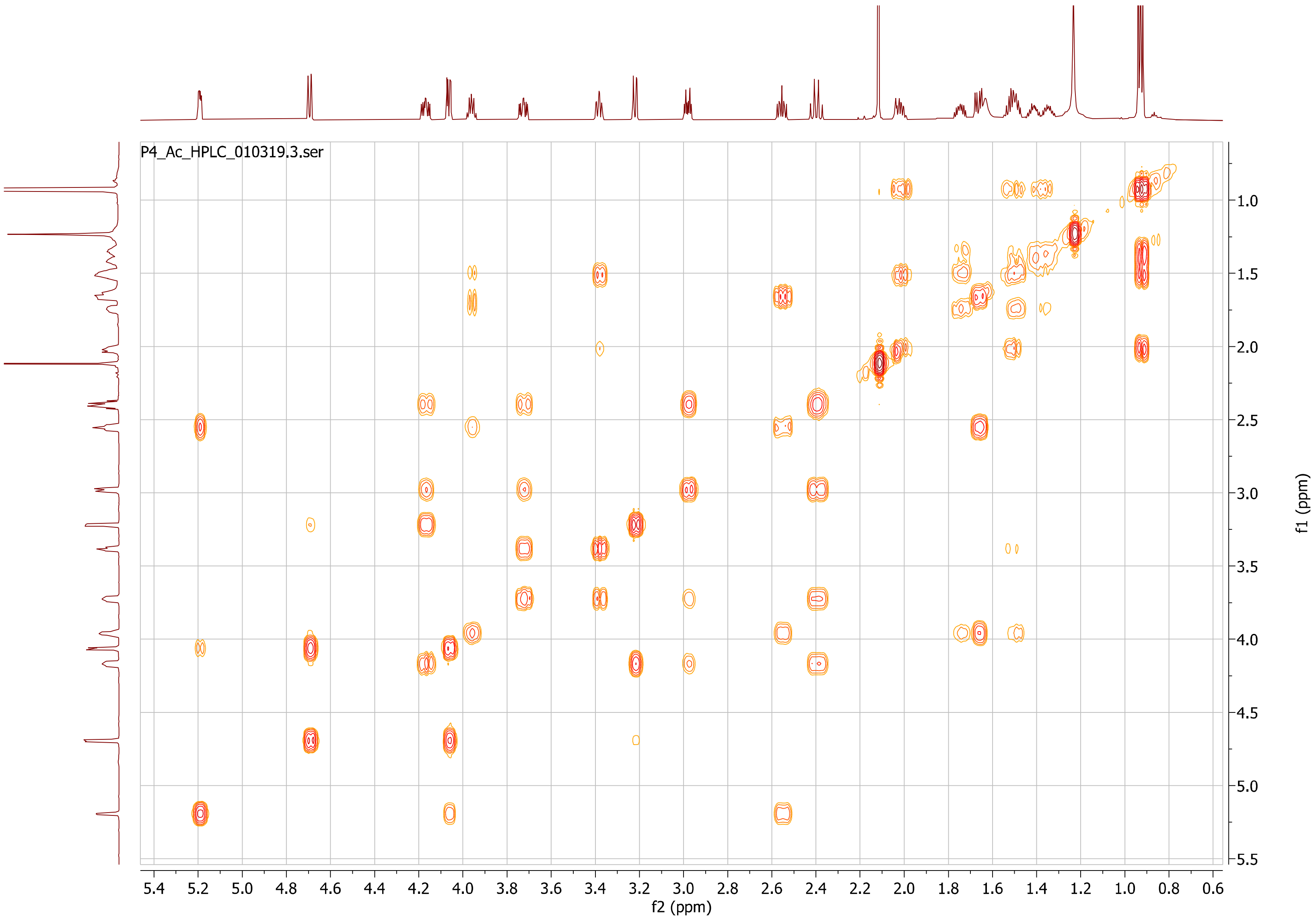


**Figure S11:** 2D COSY spectrum of compound **6** ($CDCl_3$, 700 MHz).

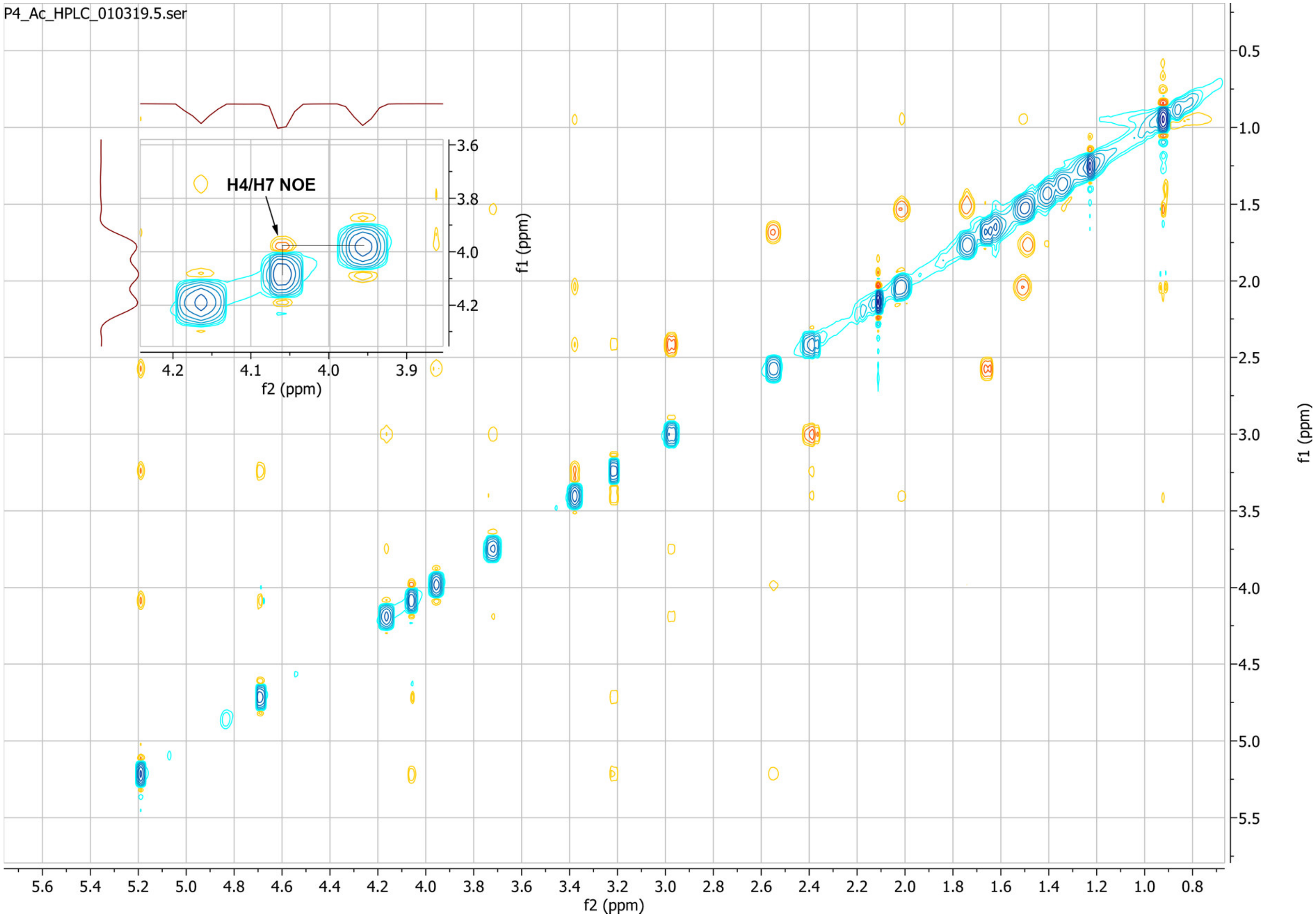


**Figure S12:** 2D NOESY spectrum of compound **6**. The NOE correlation between H4 and H7 protons is highlighted in the inset (600 ms mixing time, $CDCl_3$, 700 MHz).

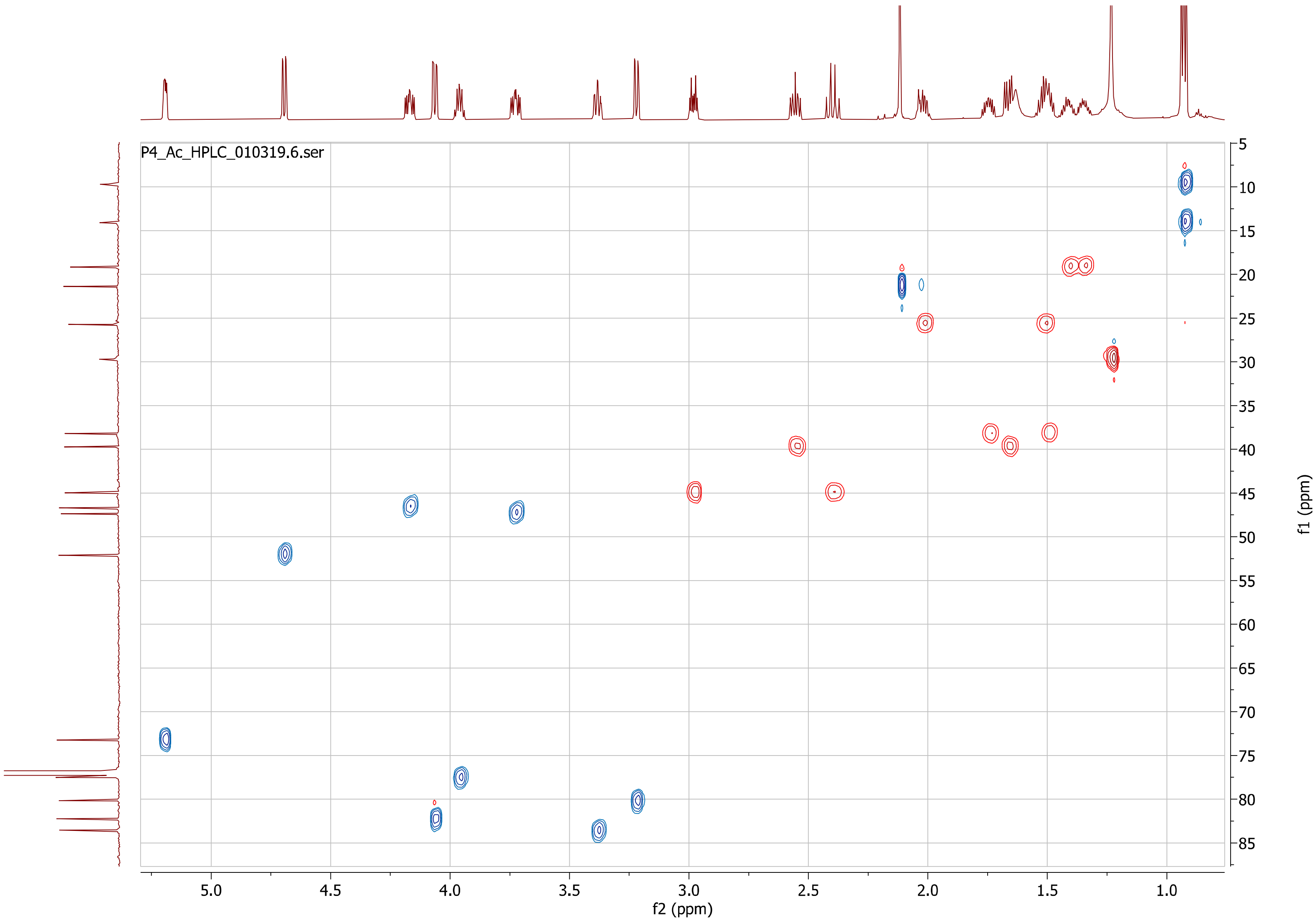


**Figure S13:** 2D HSQC spectrum of compound **6** ($CDCl_3$, 700 MHz).

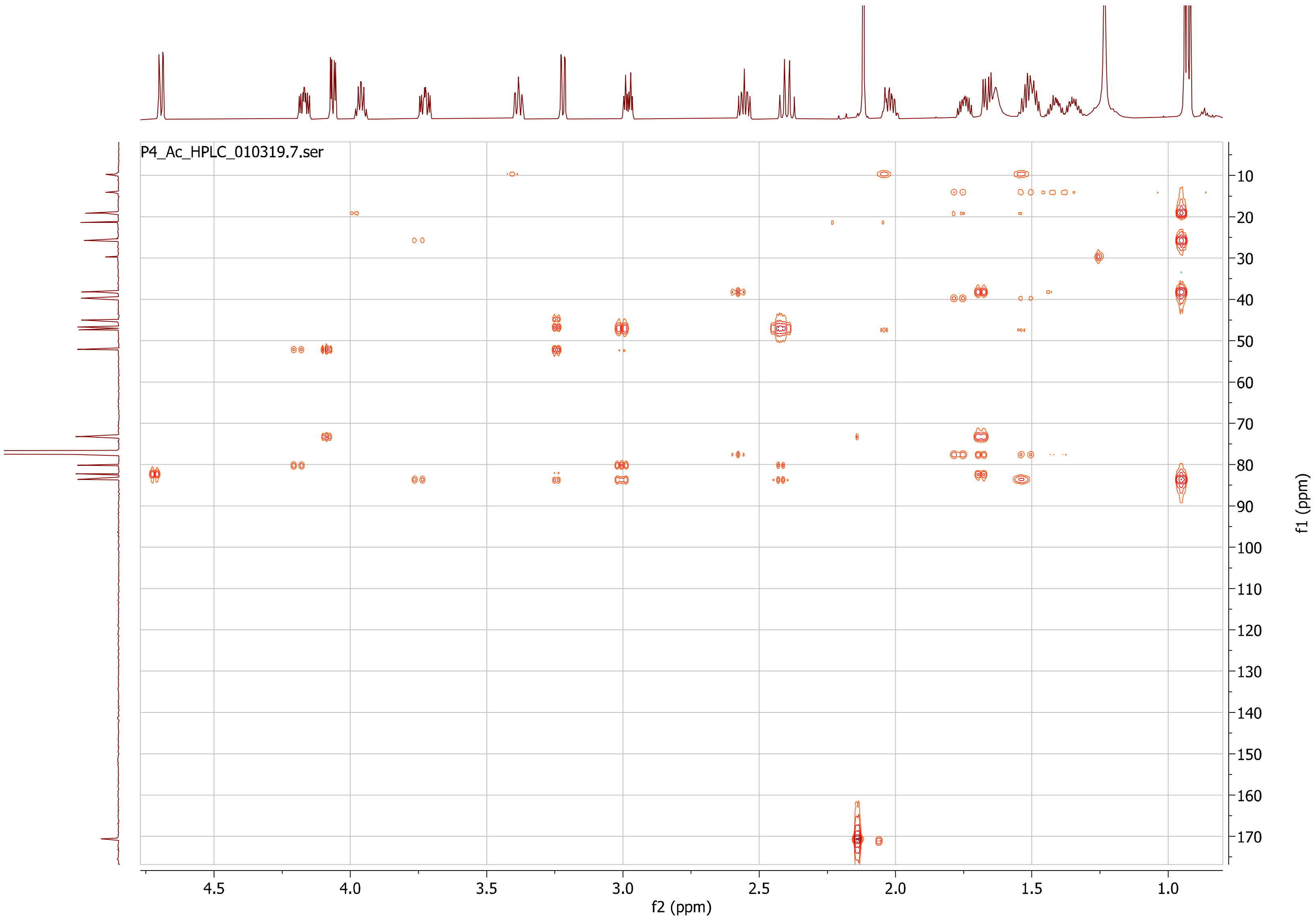


**Figure S14:** 2D HMBC spectrum of compound **6** ($CDCl_3$, 700 MHz).

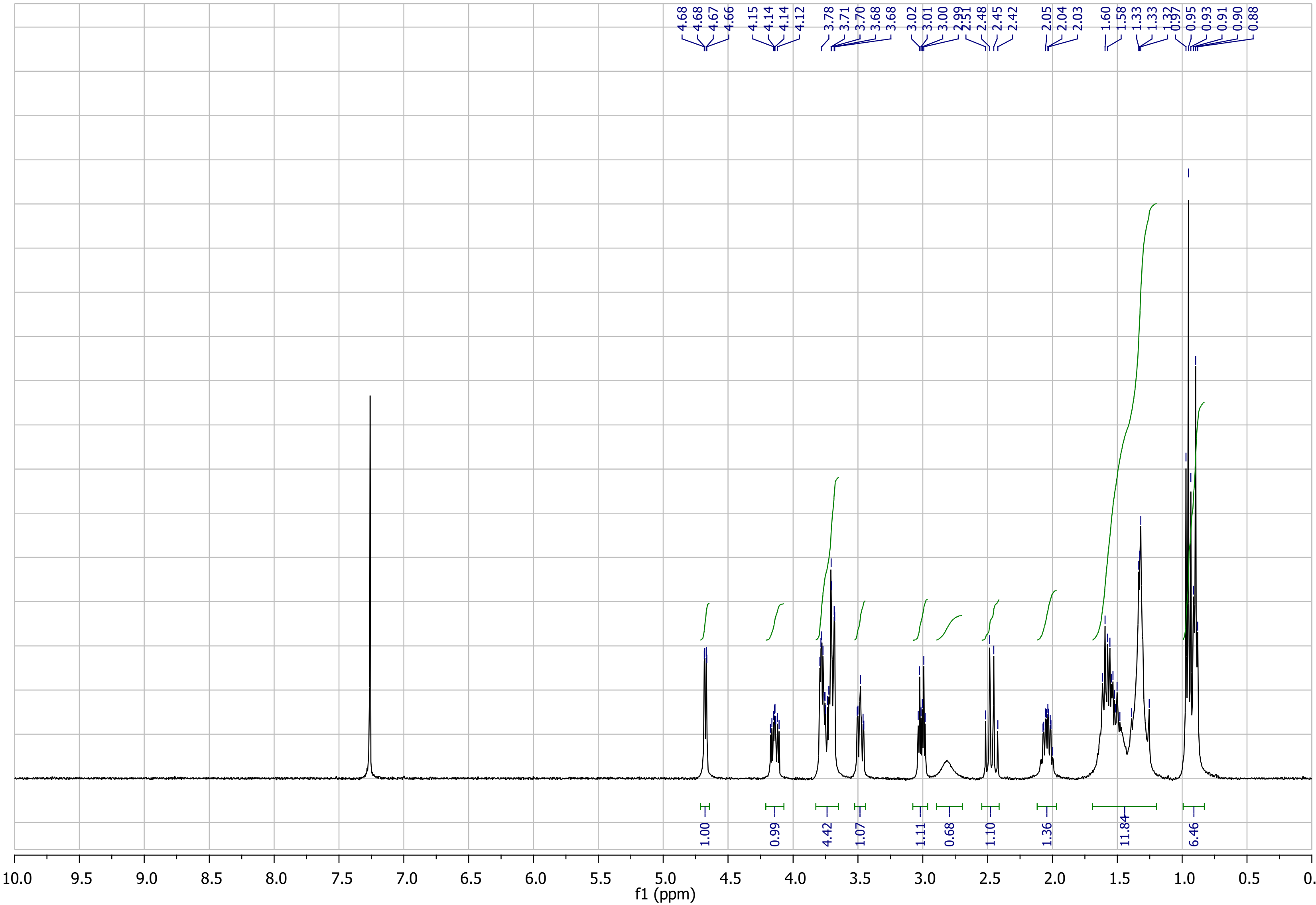


**Figure S15:** $^{1}$H NMR spectrum of compound **7** ($CDCl_3$, 400 MHz).

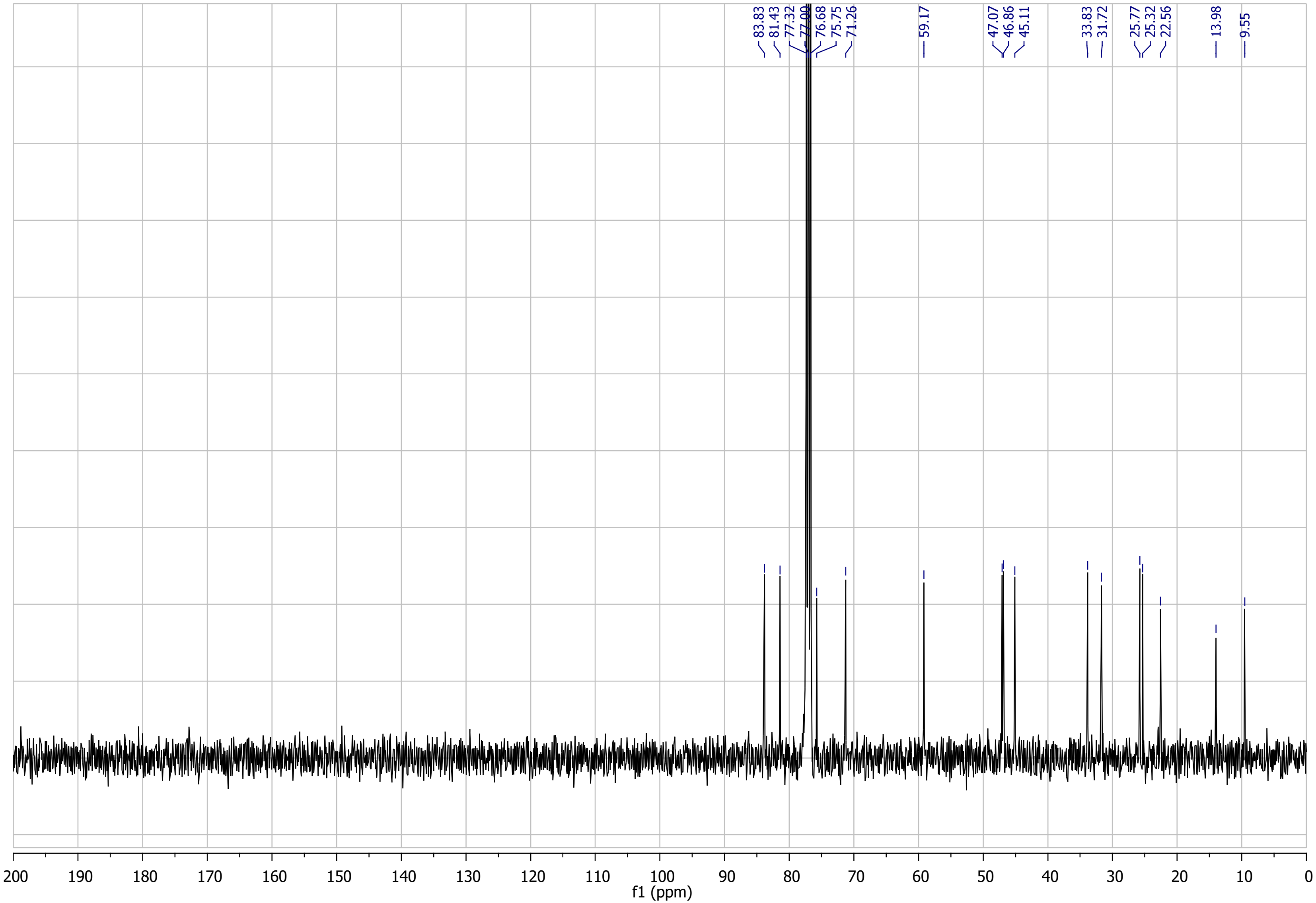


**Figure S16:** $^{13}C$ NMR spectrum of compound **7** ($CDCl_3$, 100 MHz).

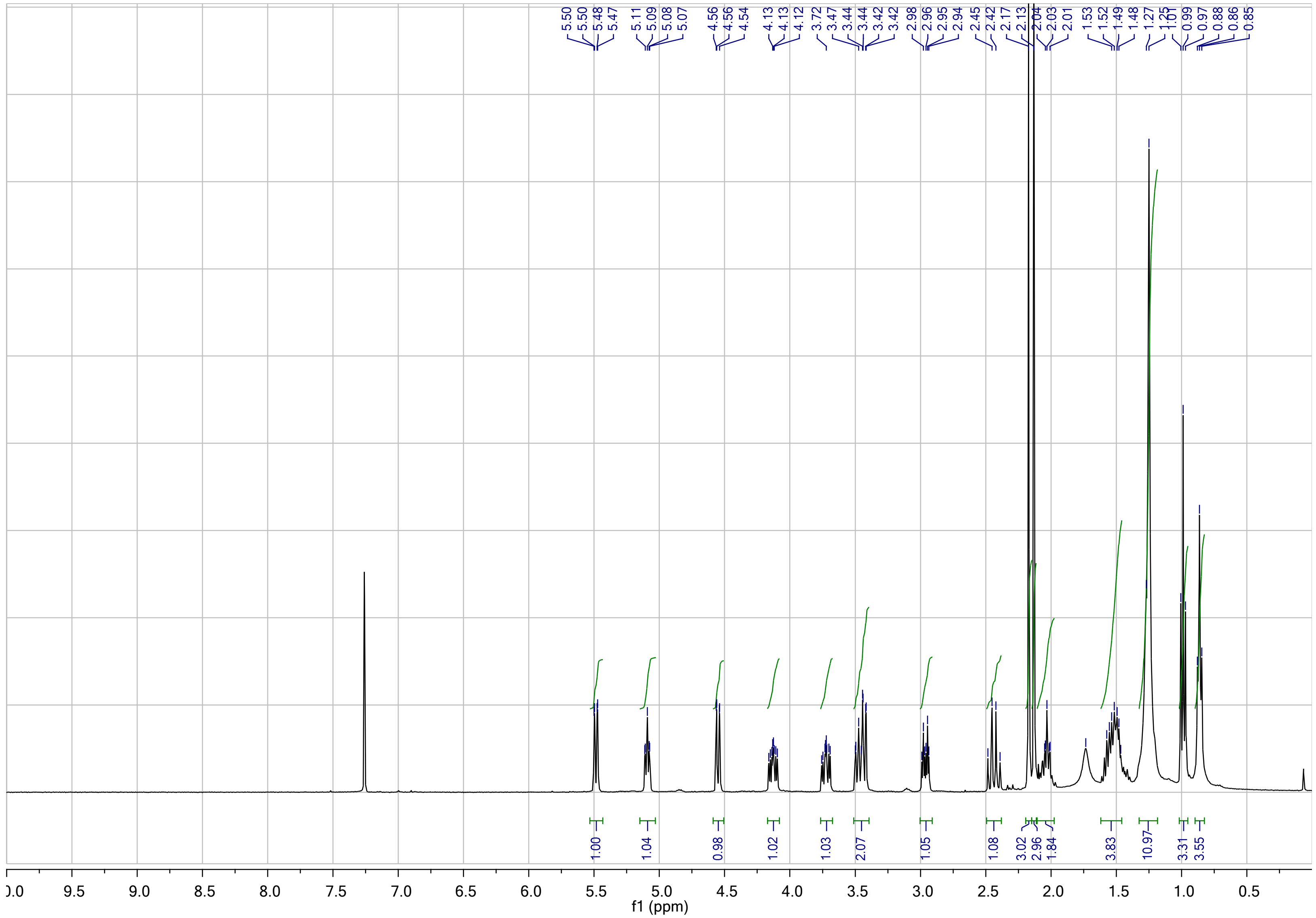


**Figure S17:** $^1$H NMR spectrum of compound **8** ($CDCl_3$, 400 MHz).

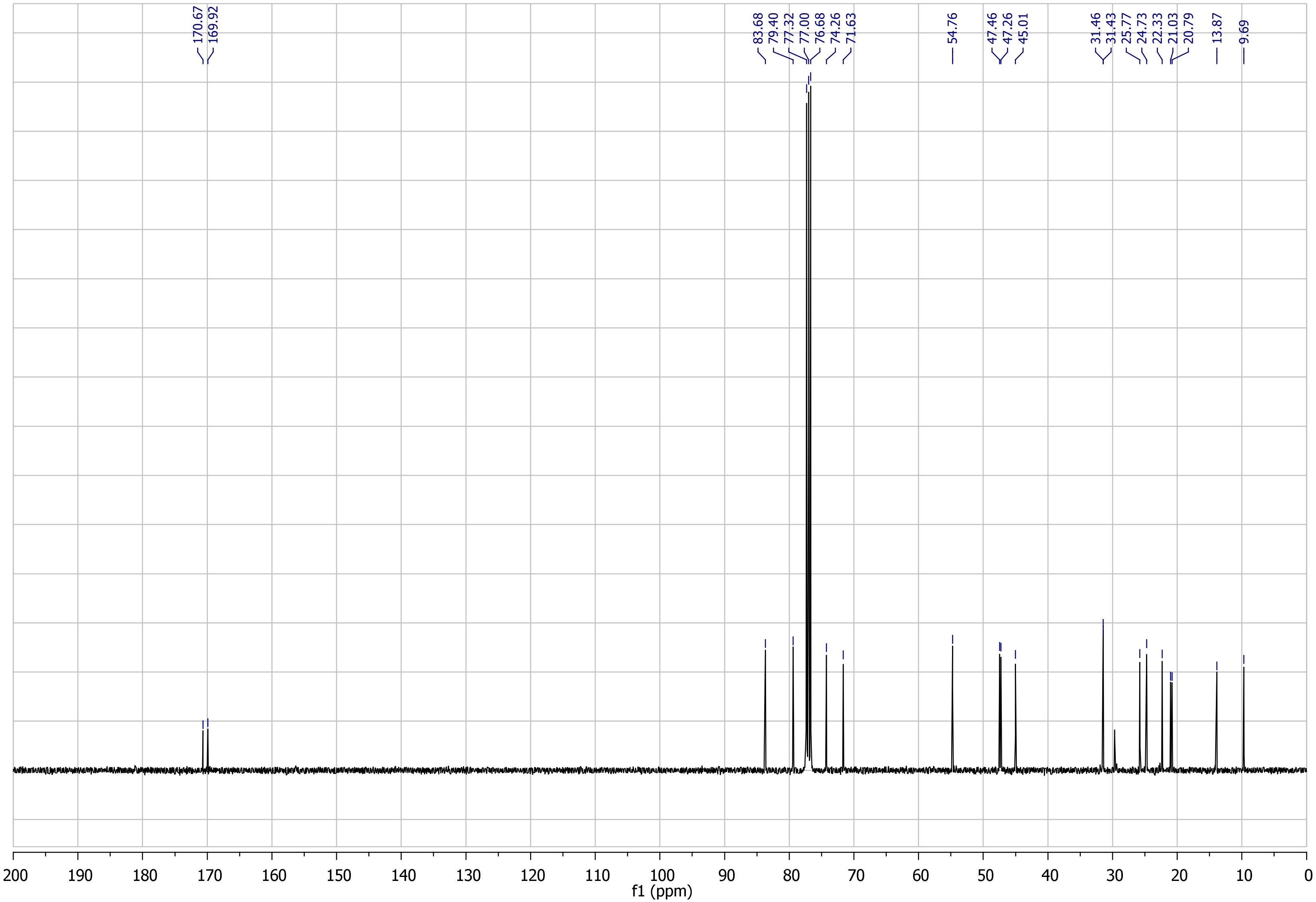


**Figure S18:** $^{13}C$ NMR spectrum of compound **8** ($CDCl_3$, 100 MHz).

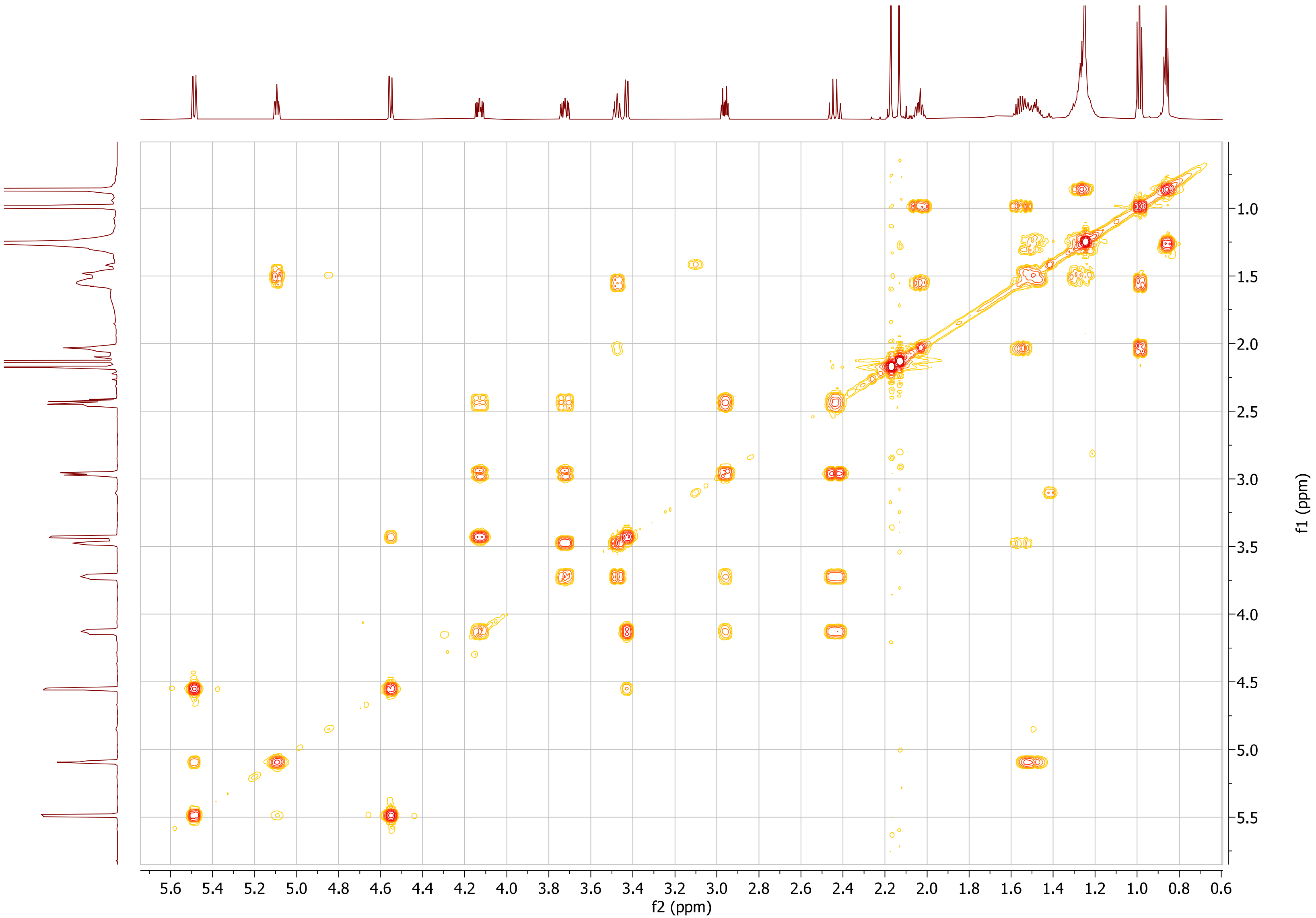


**Figure S19:** 2D COSY spectrum of compound **8** ($CDCl_3$, 700 MHz).

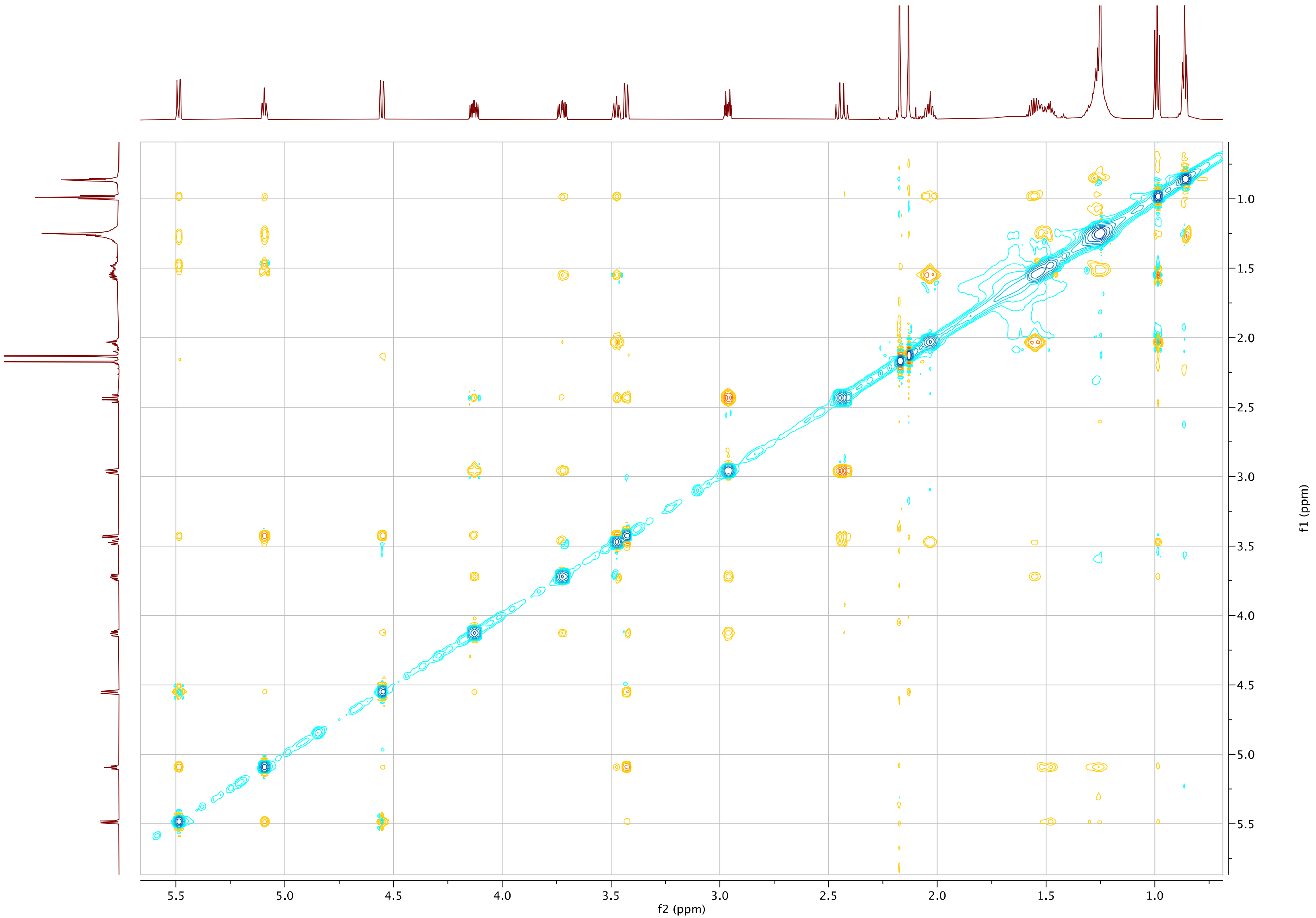


**Figure S20:** 2D NOESY spectrum of compound **8** (600 ms mixing time, $CDCl_3$, 700 MHz).

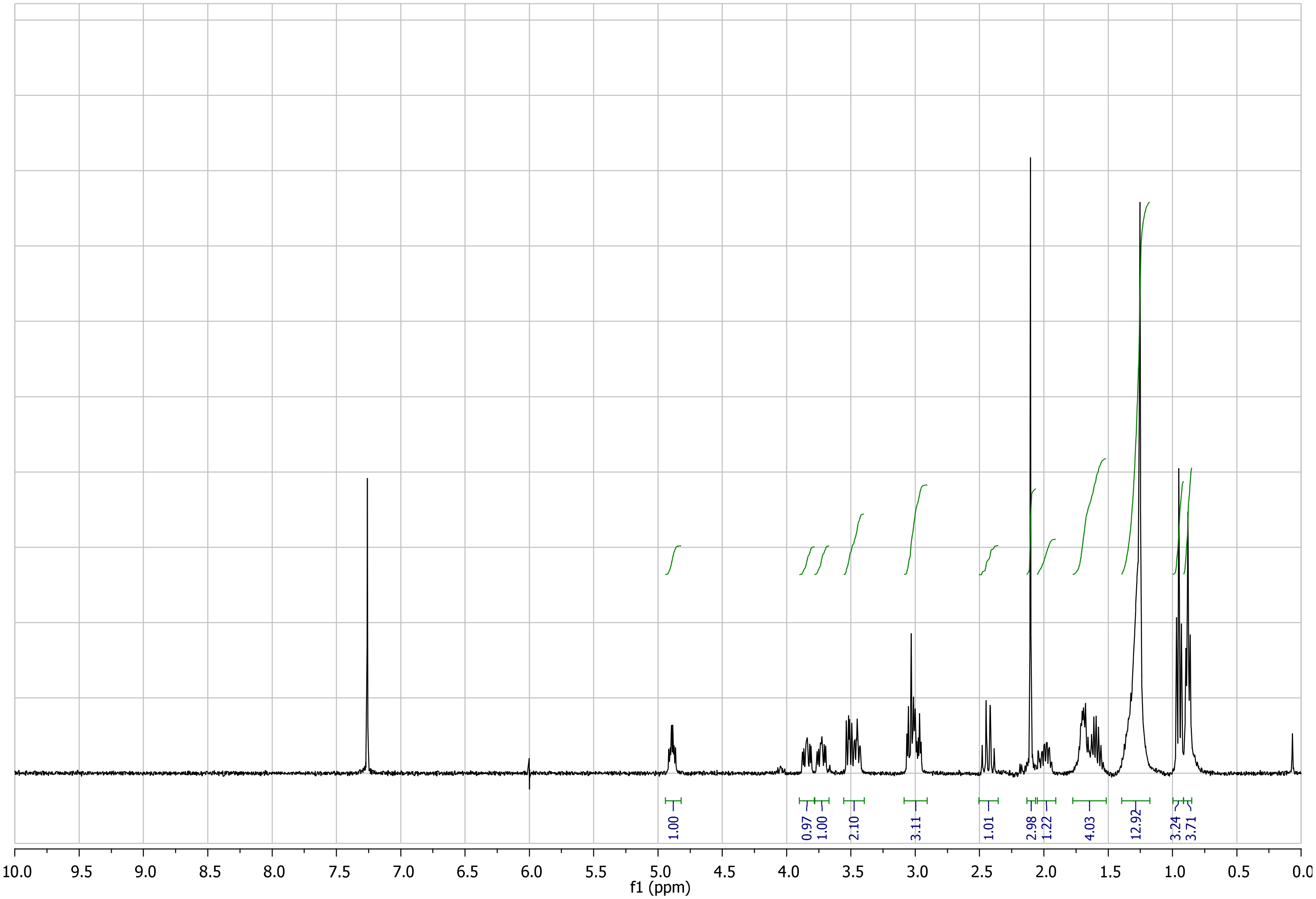


**Figure S21:** $^{1}$H NMR spectrum of compound **9** ($CDCl_3$, 400 MHz).

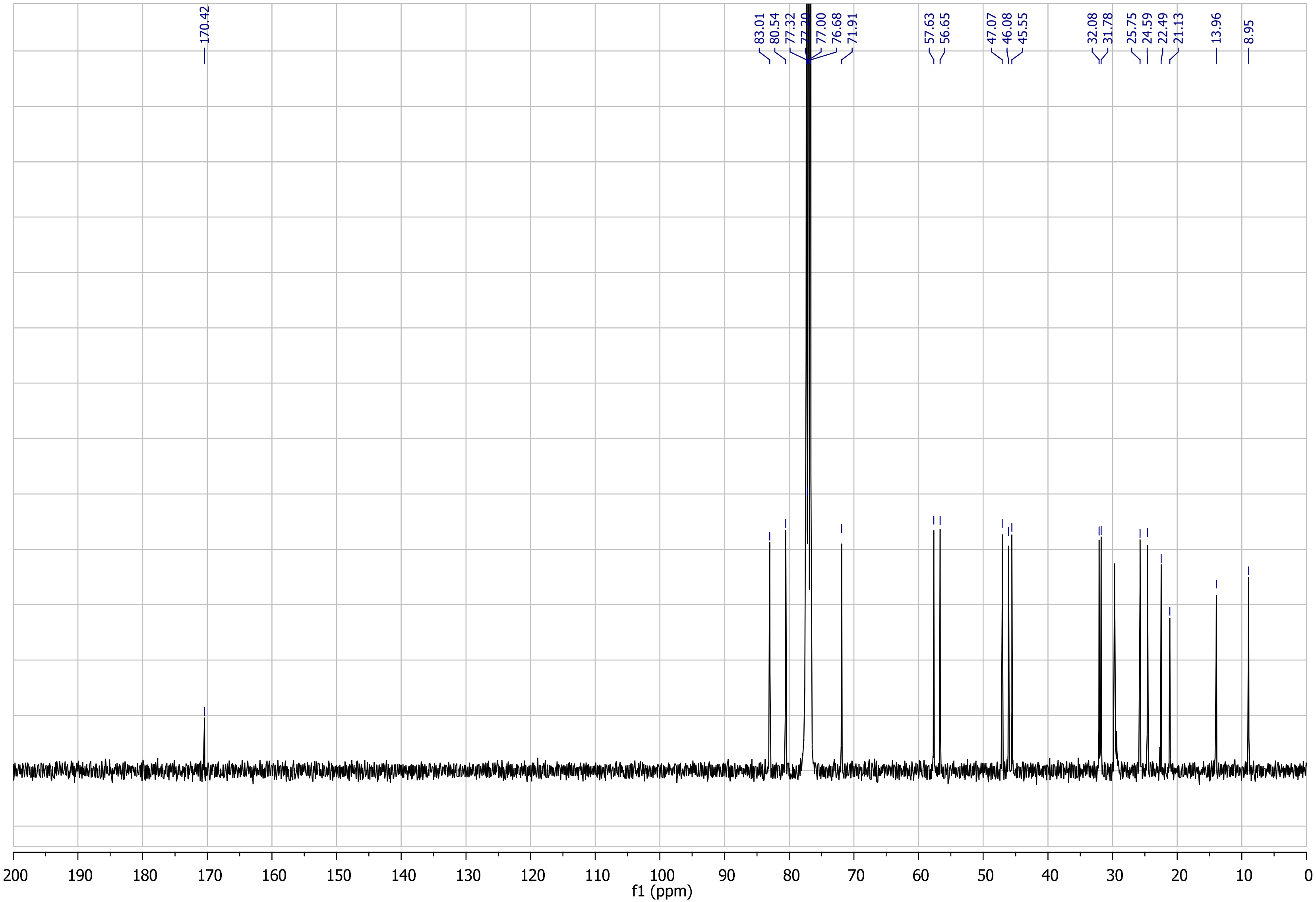


**Figure S22:** $^{13}$C NMR spectrum of compound **9** ($CDCl_3$, 100 MHz).

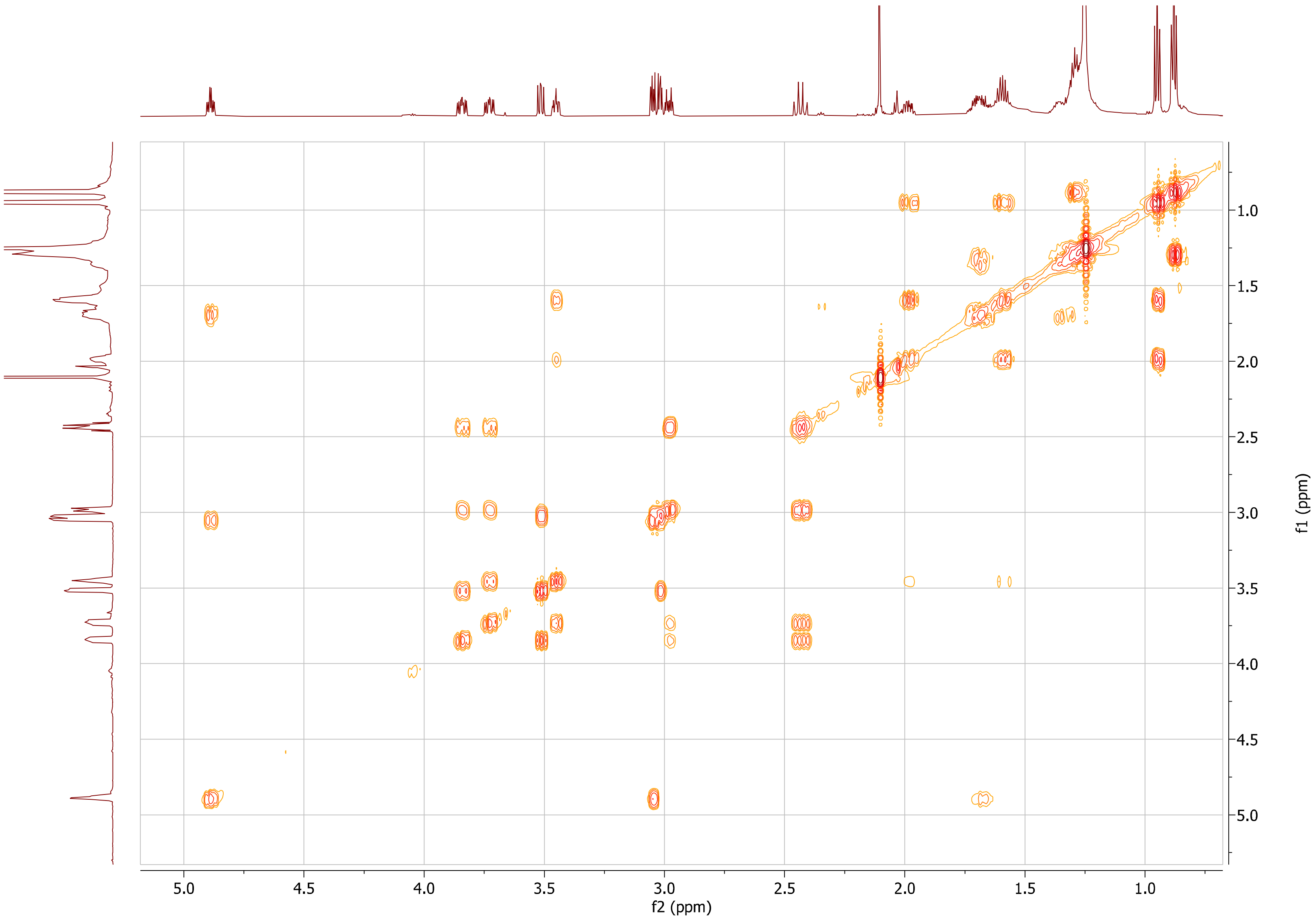


**Figure S23:** 2D COSY spectrum of compound **9** ($CDCl_3$, 700 MHz).

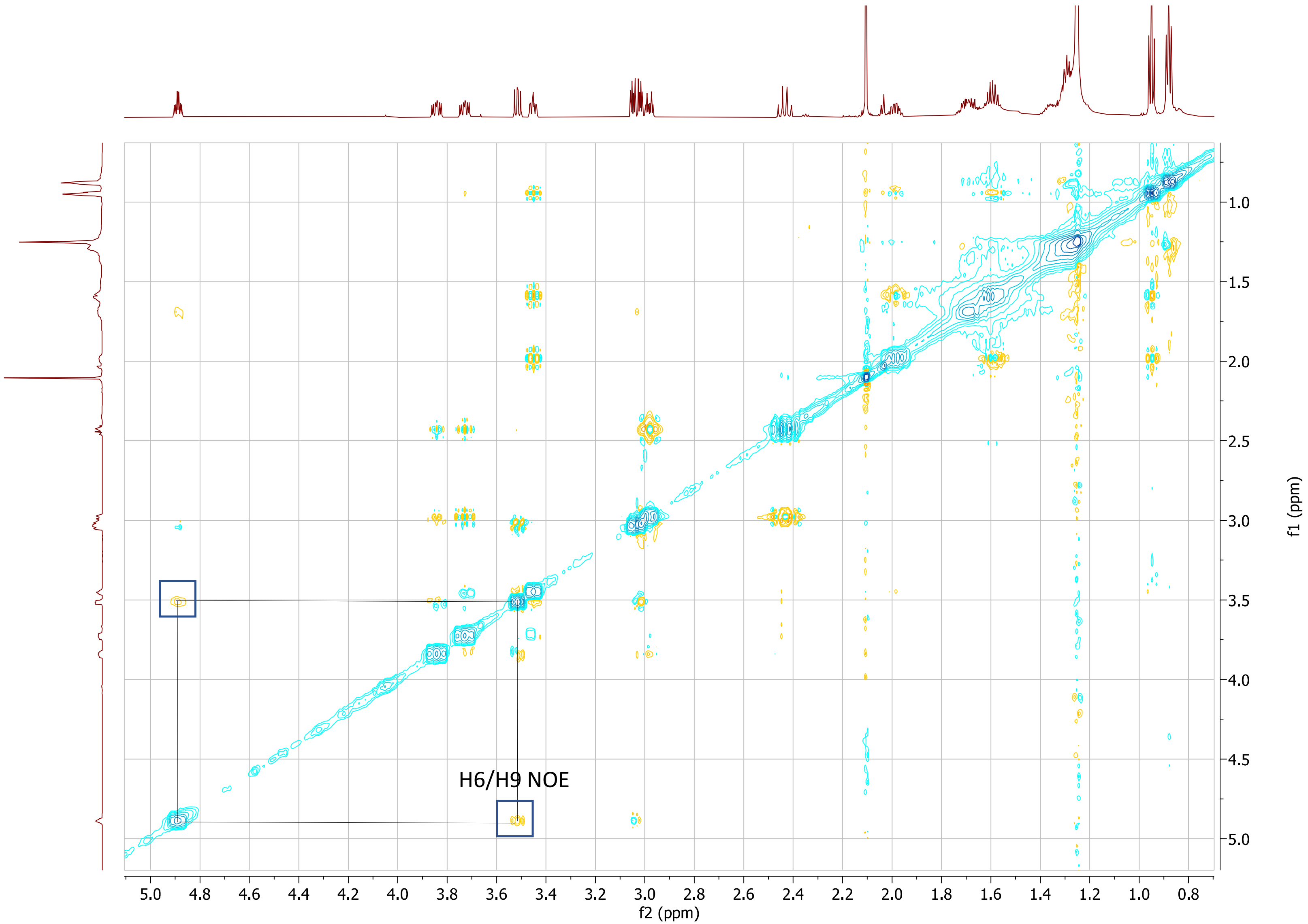


**Figure S24:** 2D NOESY spectrum of compound **9**. The NOE correlation between H6 and H9 protons is highlighted in the spectrum (600 ms mixing time, $CDCl_3$, 700 MHz).

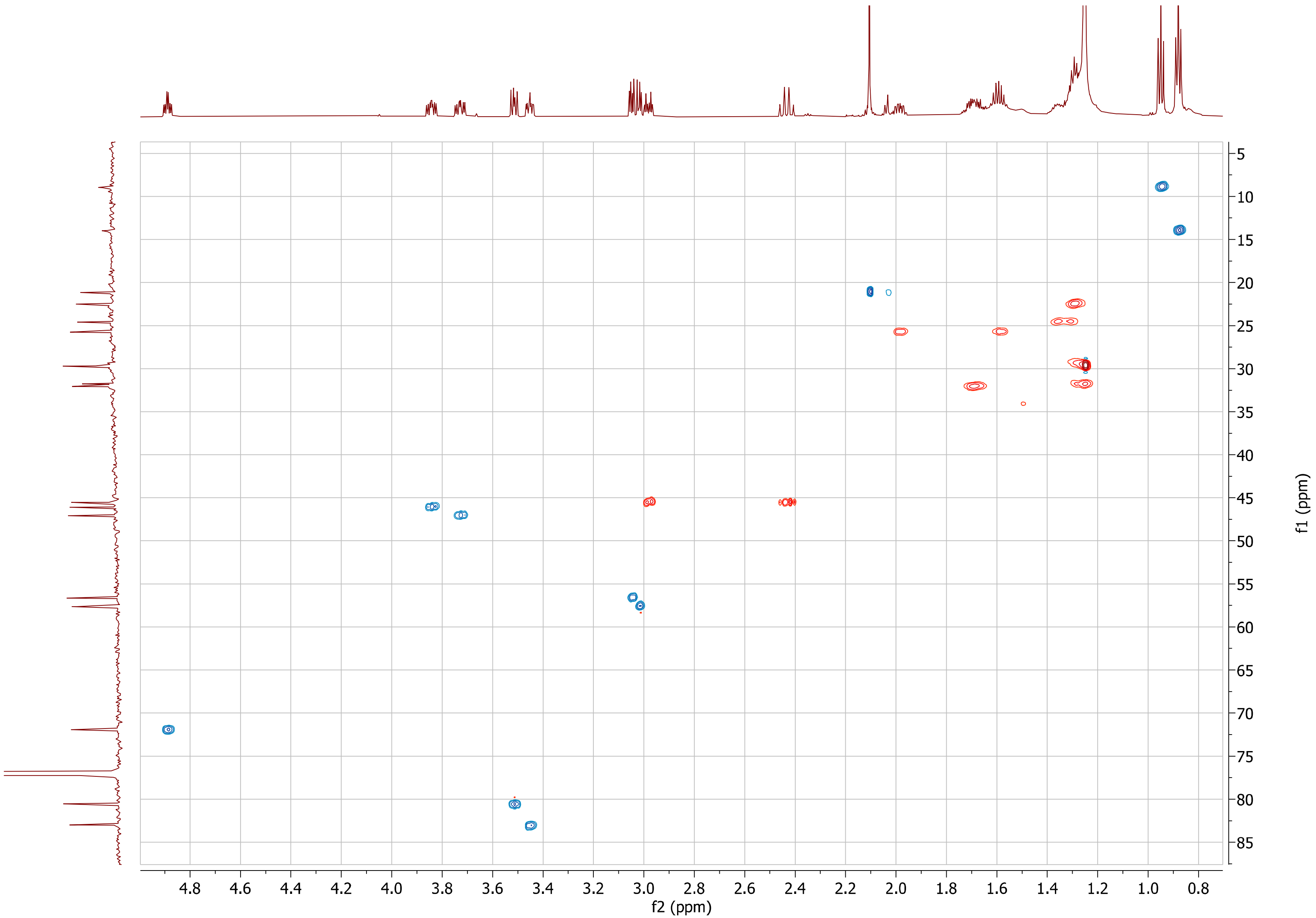


**Figure S25:** 2D HSQC spectrum of compound **9** ($CDCl_3$, 700 MHz).

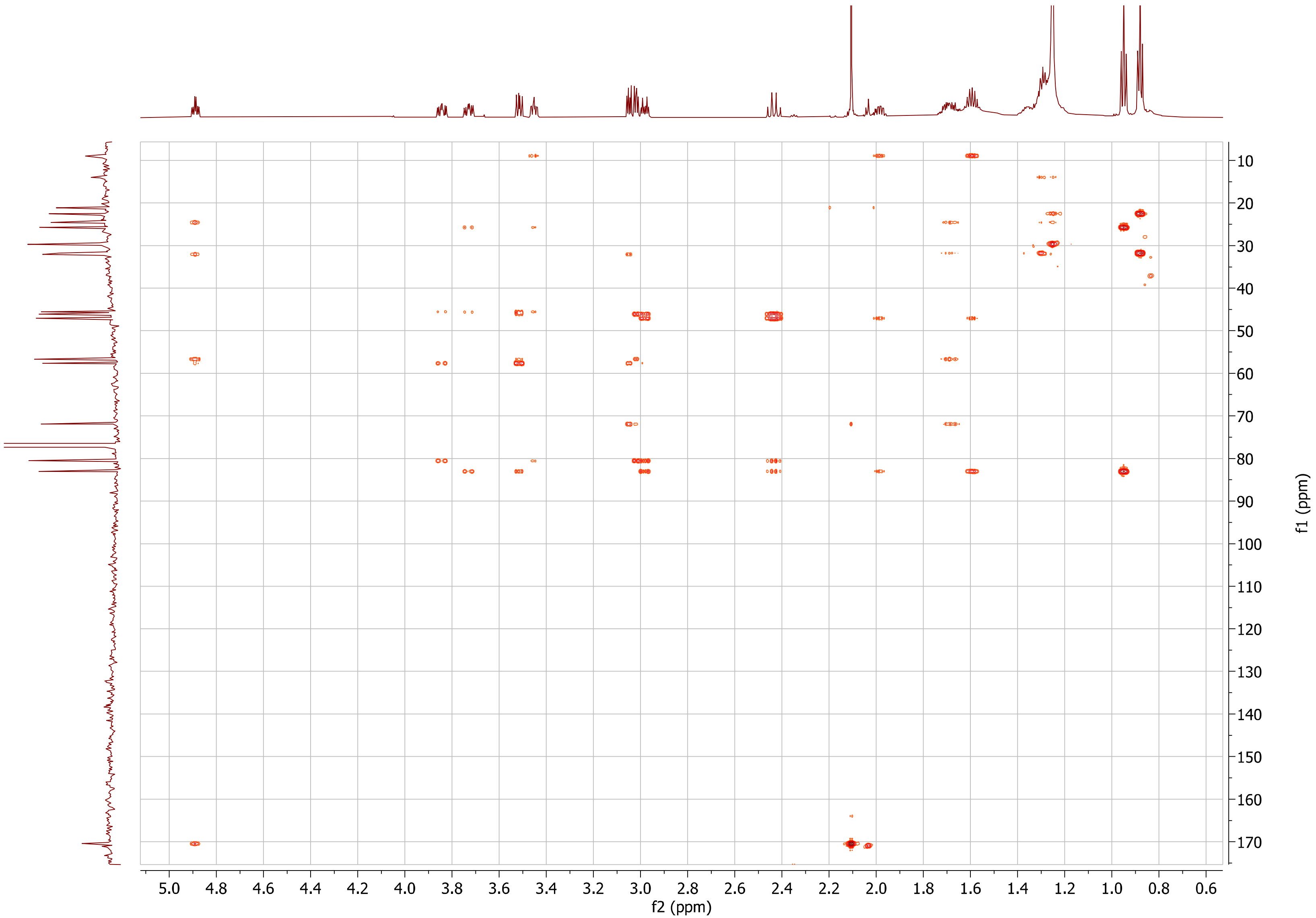


**Figure S26:** 2D HMBC spectrum of compound **9** ($CDCl_3$, 700 MHz).

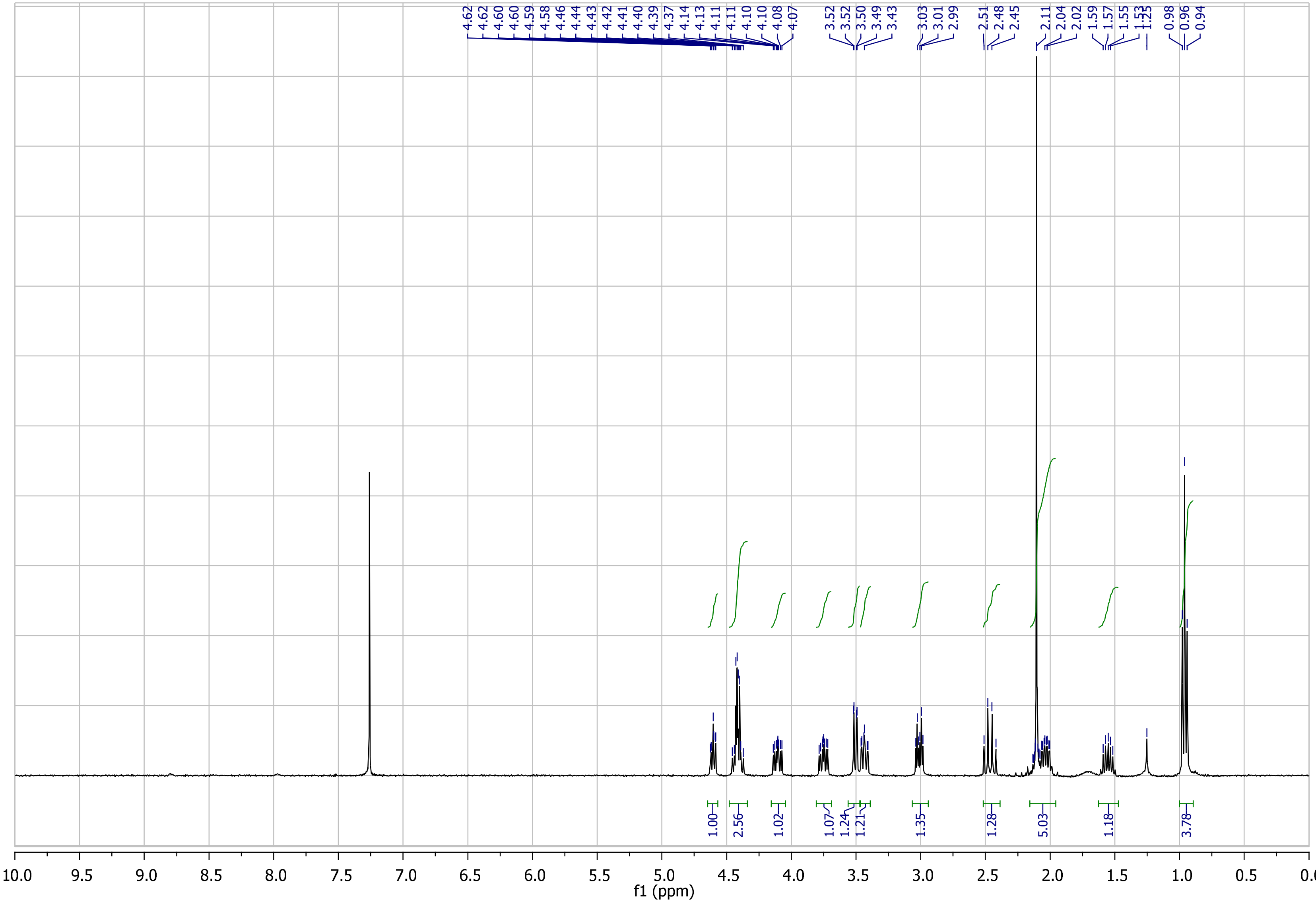


**Figure S27:** $^{1}$H NMR spectrum of compound **10** ($CDCl_3$, 400 MHz).

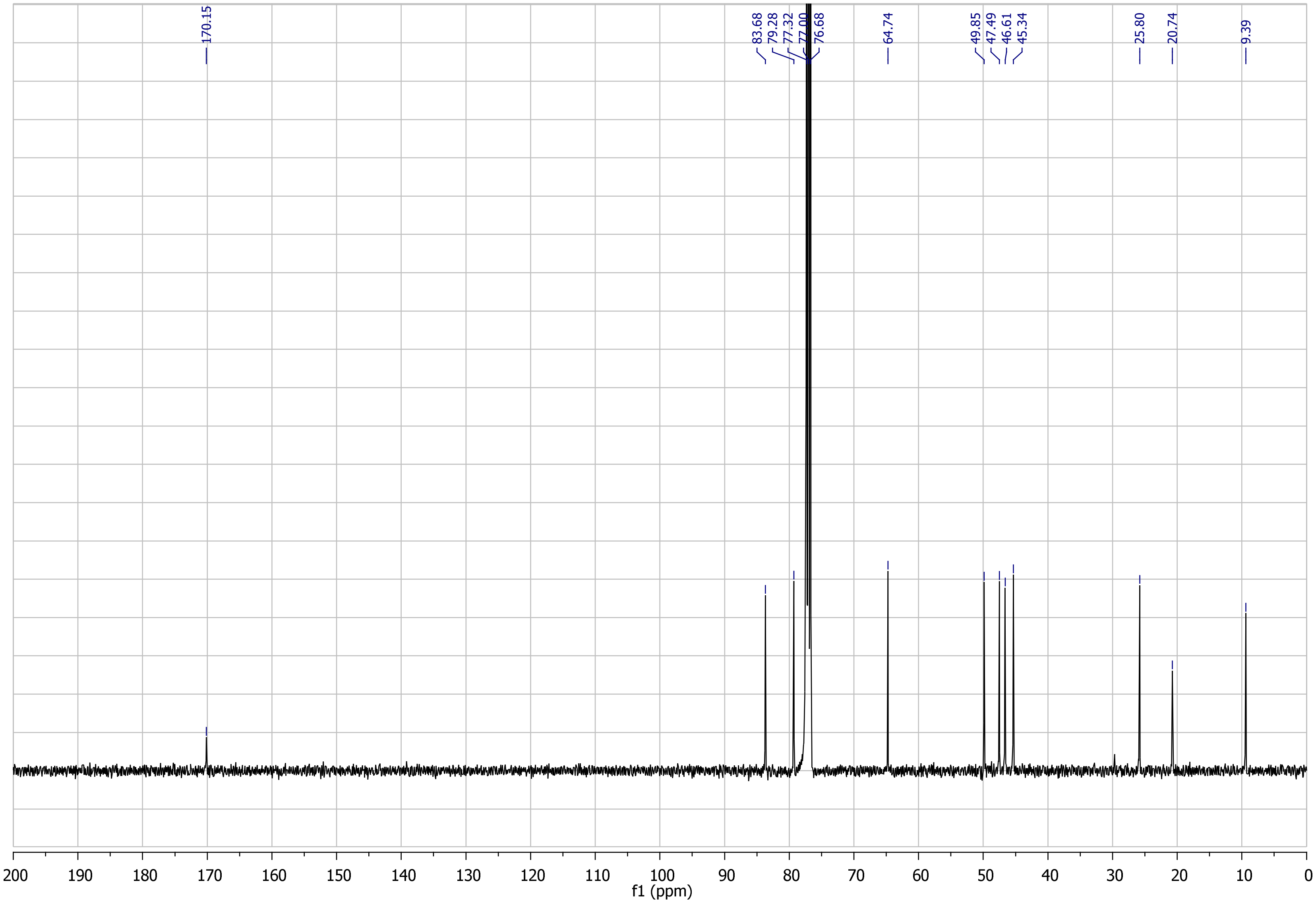


**Figure S28:** $^{13}$C NMR spectrum of compound **10** ($CDCl_3$, 100 MHz).

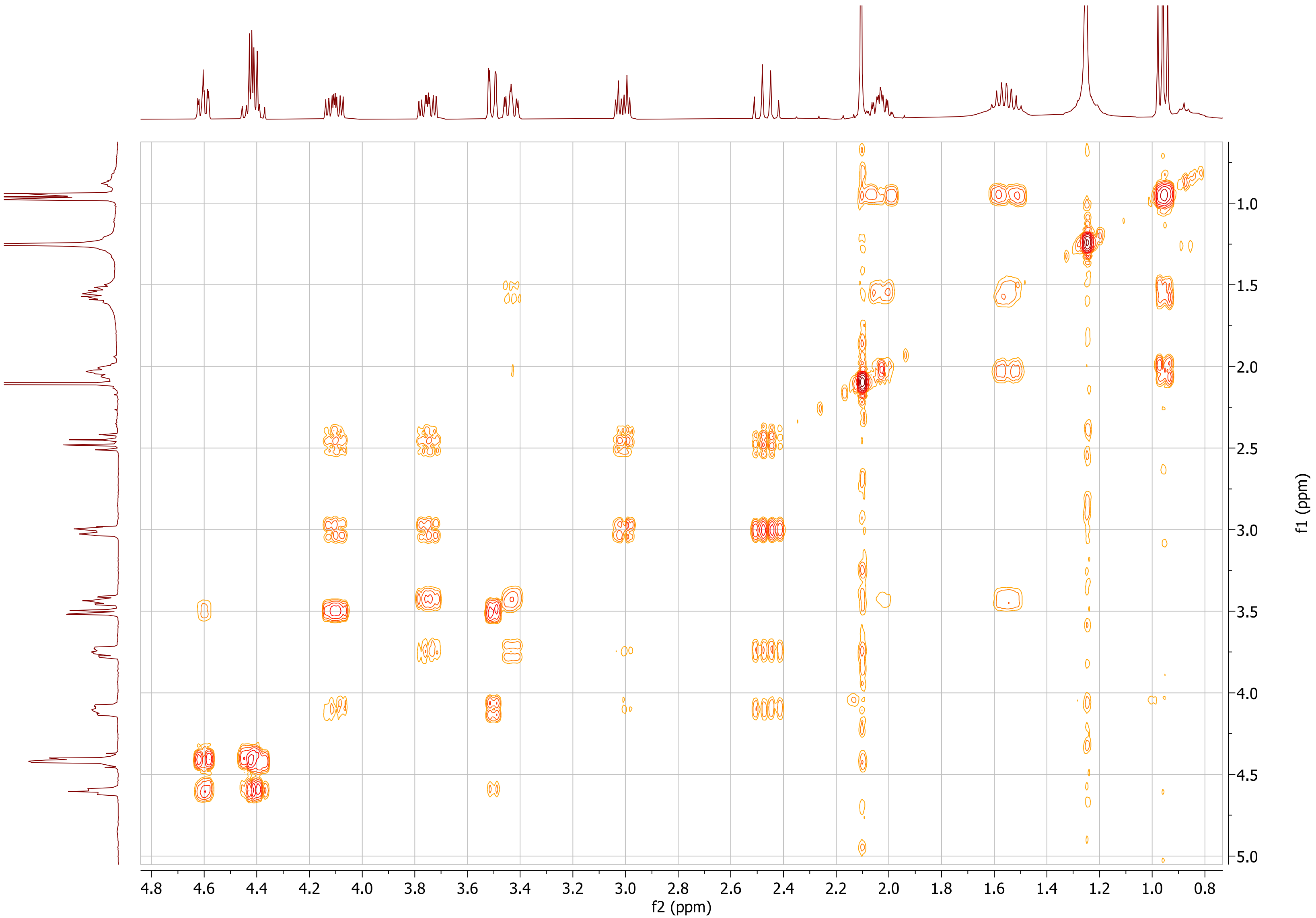


**Figure S29:** 2D COSY spectrum of compound **10** ($CDCl_3$, 400 MHz).

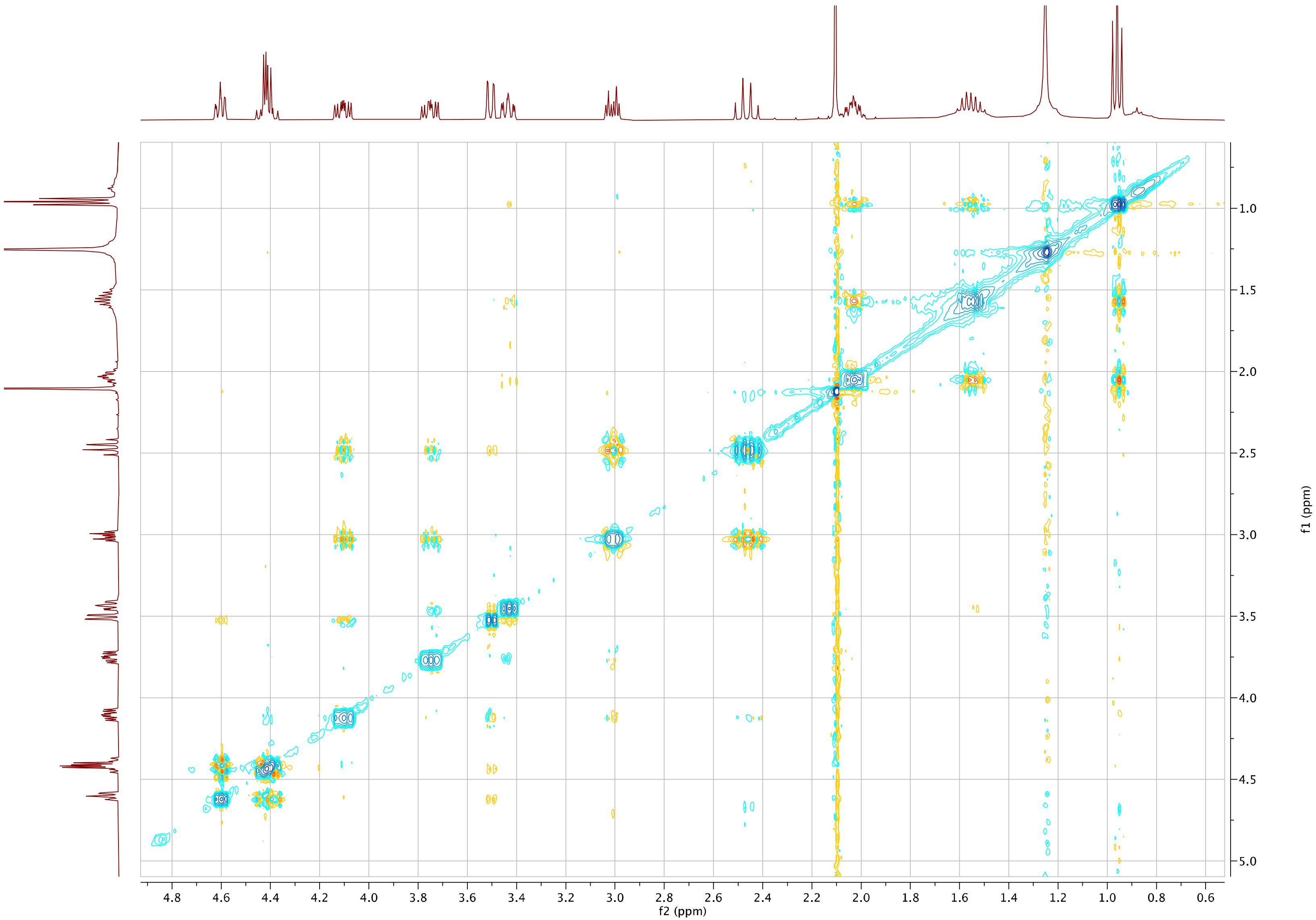


**Figure S30:** 2D NOESY spectrum of compound **10** (600 ms mixing time, $CDCl_3$, 400 MHz).

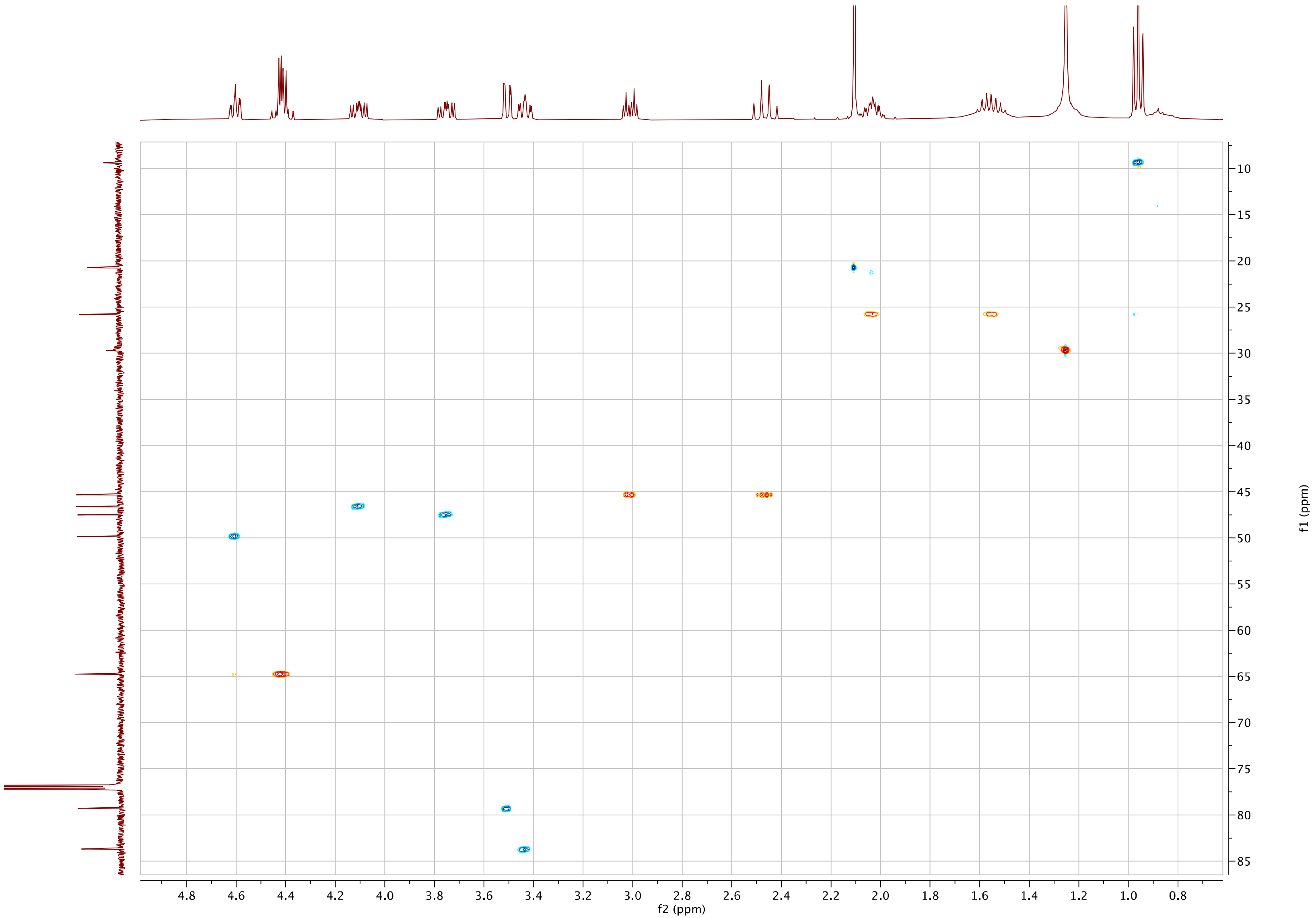


**Figure S31:** 2D HSQC spectrum of compound **10** ($CDCl_3$, 700 MHz).

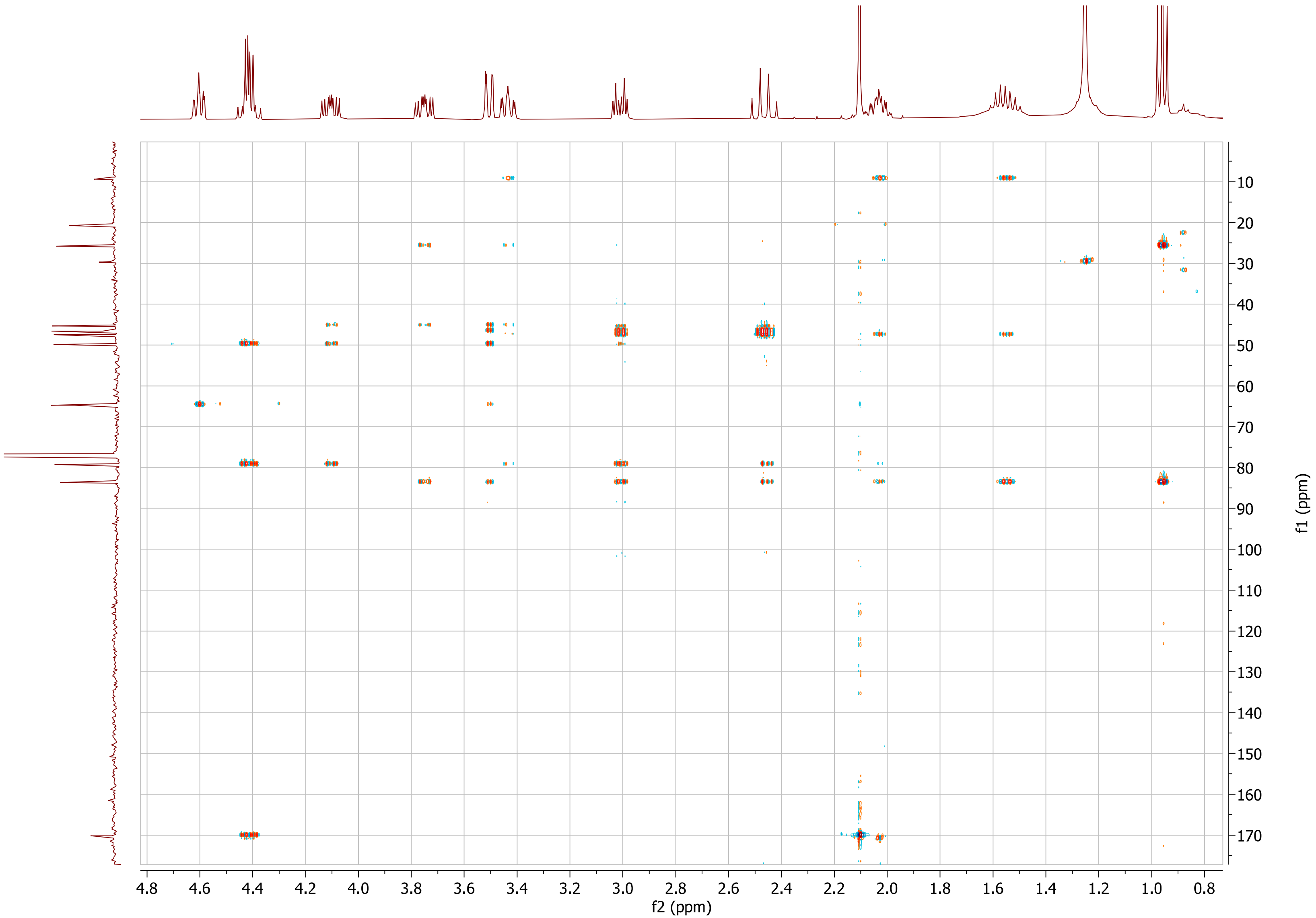


**Figure S32:** 2D HMBC spectrum of compound **10** ($CDCl_3$, 700 MHz).

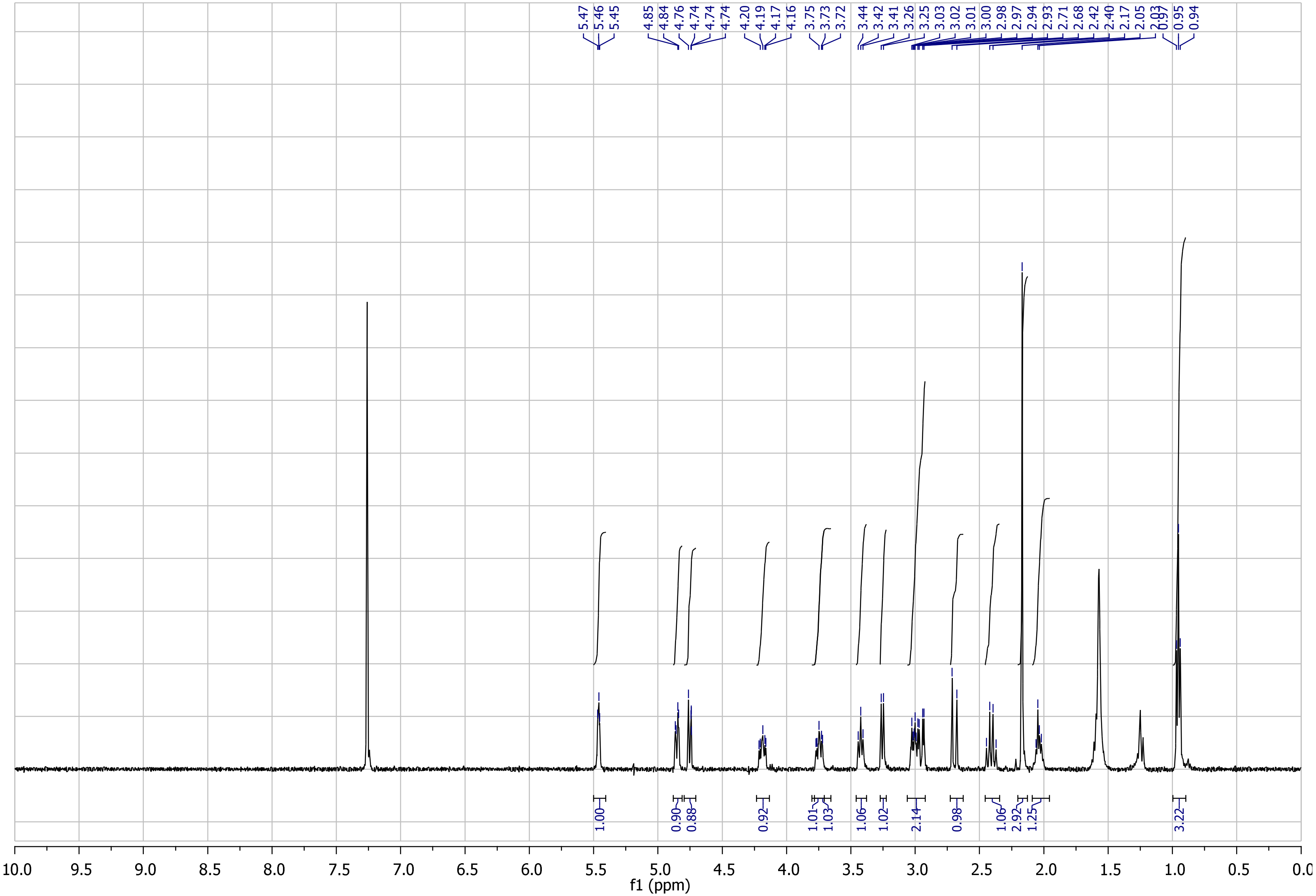

**Figure S33:** $^{1}$H NMR spectrum of compound **11** ($CDCl_3$, 400 MHz).

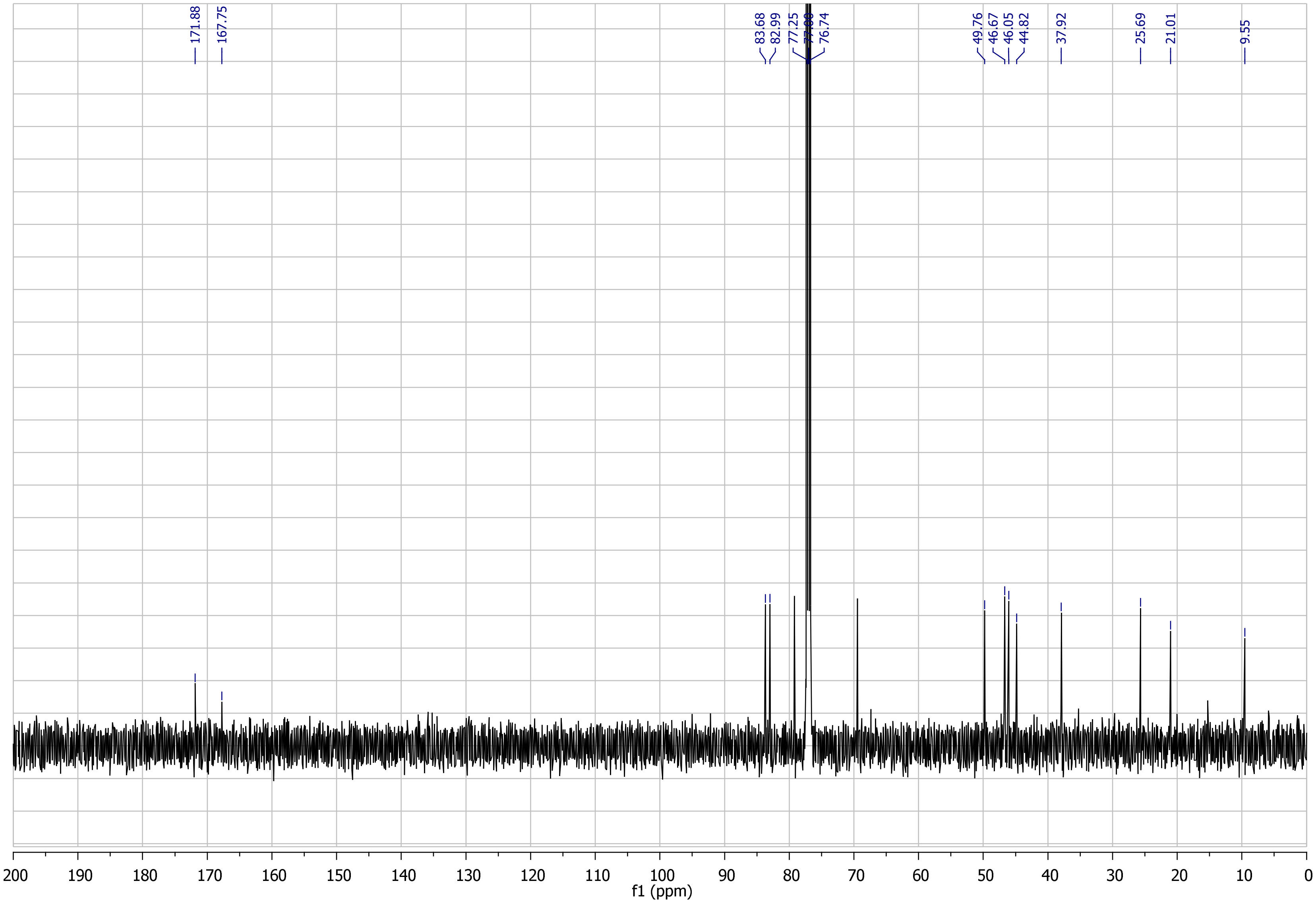


**Figure S34**: $^{13}C$ NMR spectrum of compound **11** ($CDCl_3$, 100 MHz).

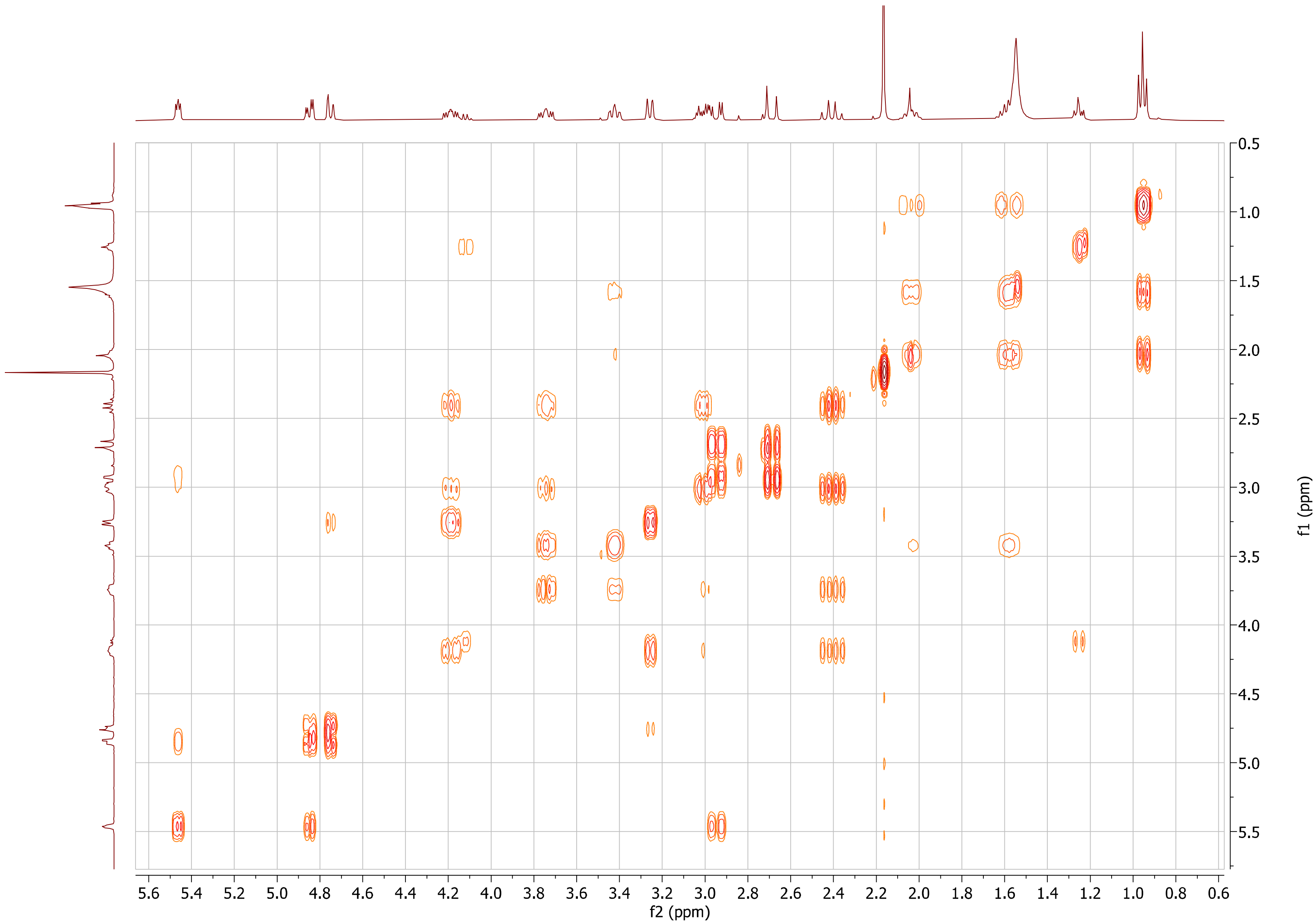


**Figure S35:** 2D COSY spectrum of compound **11** ($CDCl_3$, 700 MHz).

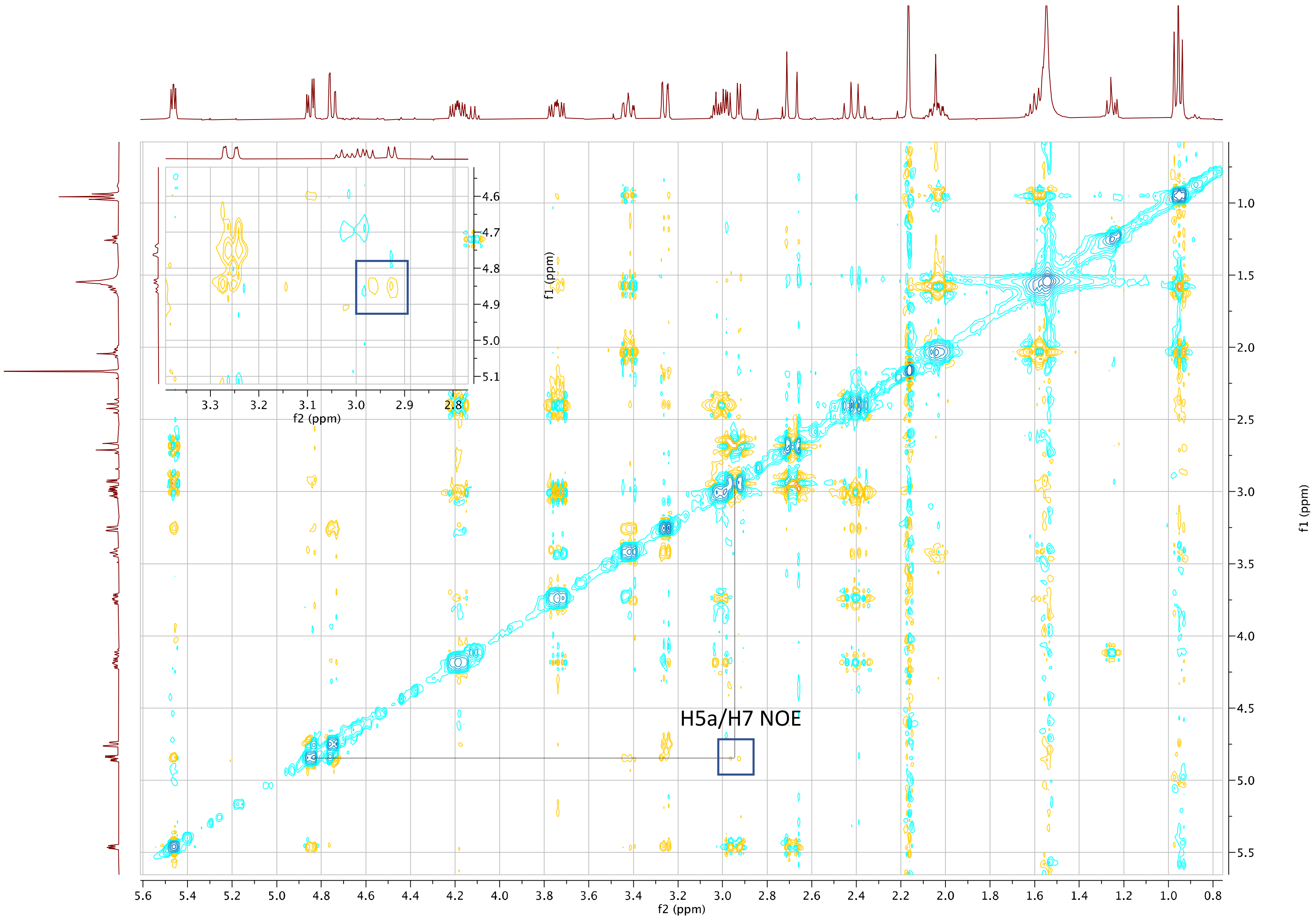


**Figure S36**: 2D NOESY spectrum of compound **11**. The NOE correlation between H5a and H7 protons is highlighted in the spectrum and in the inset (mixing time 600 ms, $CDCl_3$, 700 MHz).

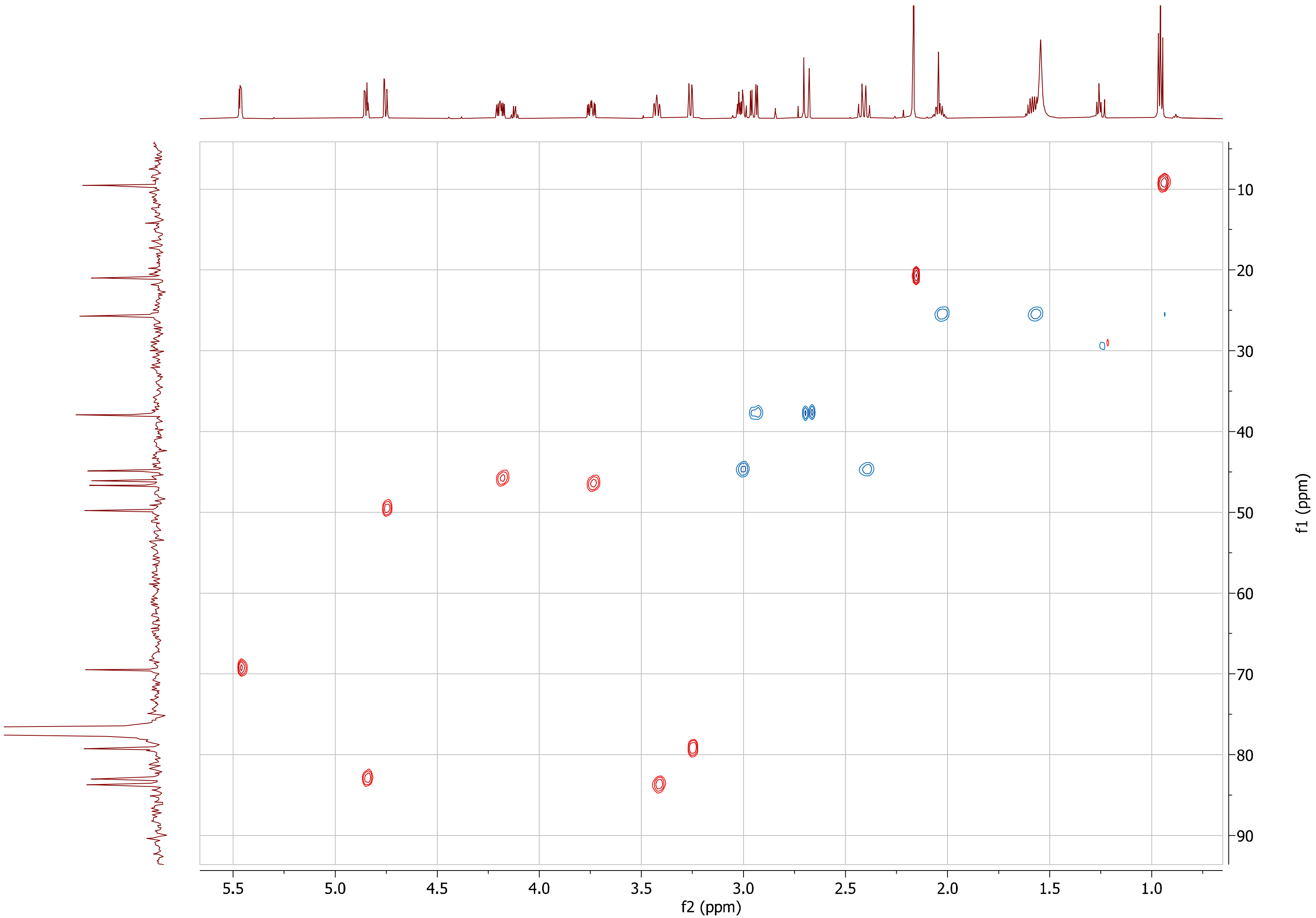


**Figure S37:** 2D HSQC spectrum of compound **11** ($CDCl_3$, 700 MHz).

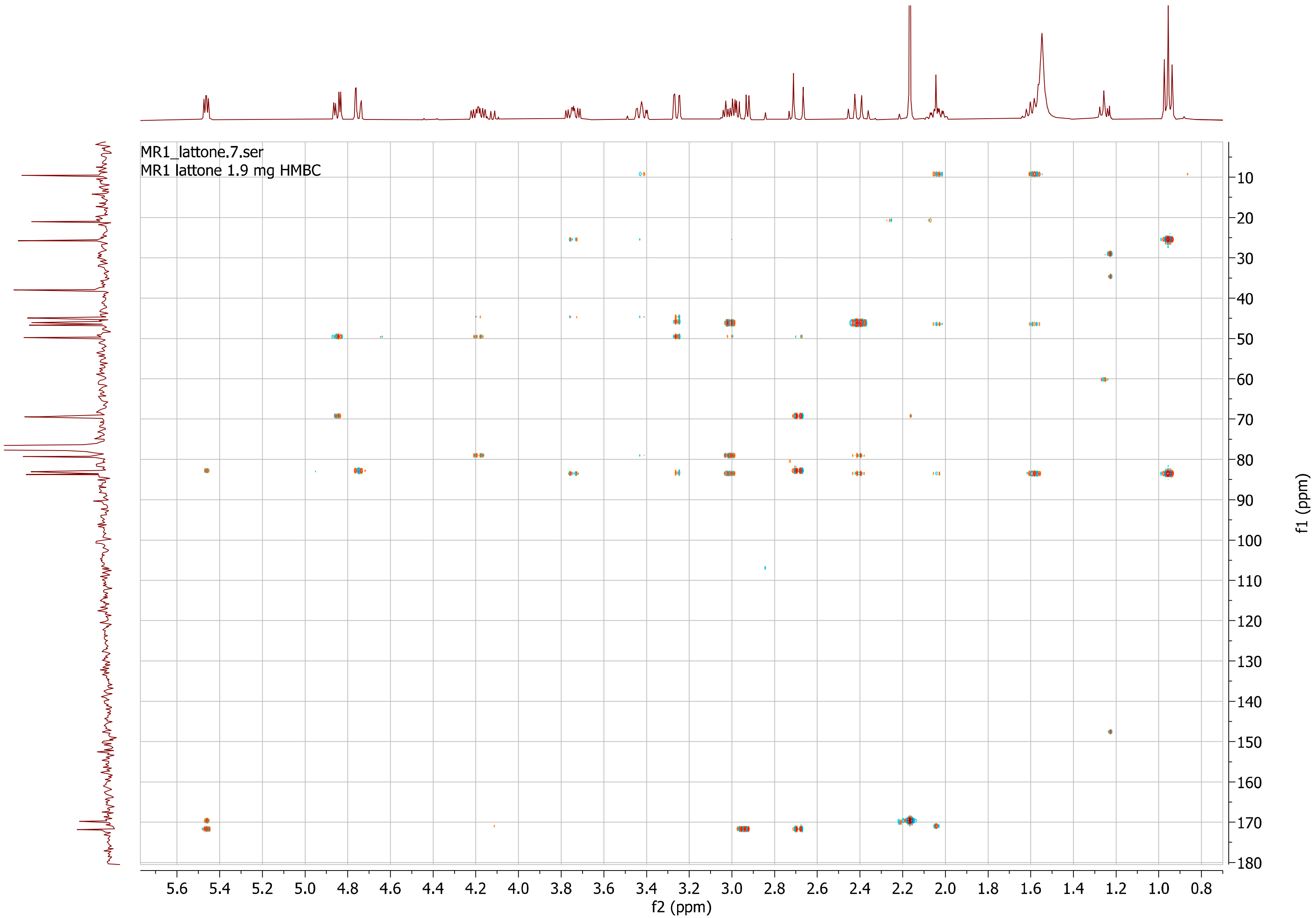


**Figure S38**: 2D HMBC spectrum of compound **11** ($CDCl_3$, 700 MHz).

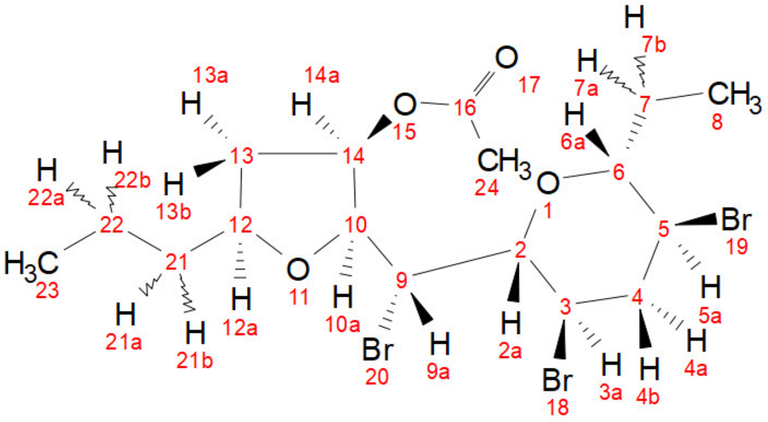

**6**

**Table S2**. $^{1}$H and $^{13}$C chemical shift data for compound **6** (700 MHz, $CDCl_3$)[a]

| position | $\delta_H$[b] | $\delta_C$ | mult[c] |
|---|---|---|---|
| 2 | 3.22 dd (9.6, 1.6) | 80.2 | CH |
| 3 | 4.17 ddd (12.1, 9.6, 4.6) | 46.7 | CH |
| 4 | b) 2.40 ddd (13.0, 12.1, 12.0)<br>a) 2.98 ddd (13.0, 4.3, 4.3) | 45.0 | $CH_2$ |
| 5 | 3.73 ddd (12.0, 10.0, 4.3) | 47.4 | CH |
| 6 | 3.38 ddd (10.0, 8.8, 2.4) | 83.6 | CH |
| 7 | 1.50 m, 2.02 m | 25.7 | $CH_2$ |
| 8 | 0.93 t (7.4) | 9.7 | $CH_3$ |
| 9 | 4.69 dd (9.7, 1.6) | 52.1 | CH |
| 10 | 4.06 dd (9.7, 3.1) | 82.3 | CH |
| 12 | 3.96 dq (8.4, 6.4) | 77.5 | CH |
| 13 | a) 1.66 ddd (14.5, 6.4, 1.1)<br>b) 2.55 ddd (14.5, 8.4, 6.3) | 39.7 | $CH_2$ |
| 14 | 5.19 ddd (6.3, 3.1, 1.1) | 73.2 | CH |
| 16 | | 170.6 | C=O |
| 21 | 1.50 m, 1.75 m | 38.2 | $CH_2$ |
| 22 | 1.35 m, 1.41 m | 19.2 | $CH_2$ |
| 23 | 0.93 t (7.4) | 14.1 | $CH_3$ |

| | | | |
|---|---|---|---|
| 24 | 2.12 s | 21.4 | $CH_3$ |

[a] The assignments were based on the COSY, TOCSY, HMQC, NOESY and HMBC experiments. [b] Coupling constants in Hz are given in parenthesis. [c] Multiplicities were assigned from the HSQC spectrum.

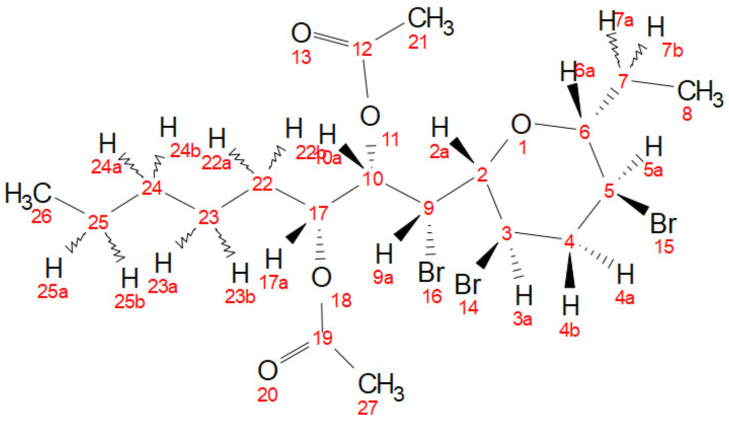

**8**

**Table S2**. $^1$H and $^{13}$C chemical shift data for compound **8** (700 MHz, $CDCl_3$)[a]

| position | $\delta_H$[b] | $\delta_C$ | mult[c] |
|---|---|---|---|
| 2 | 3.43 dd (9.5, 1.9) | 79.4 | CH |
| 3 | 4.13 ddd (12.1, 9.5, 4.5) | 47.3 | CH |
| 4 | b) 2.44 ddd (12.9, 12.1, 12.0)<br>a) 2.96 ddd (12.9, 4.5, 4.3) | 45.0 | $CH_2$ |
| 5 | 3.72 ddd (12.0, 10.0, 4.3) | 54.8 | CH |
| 6 | 3.47 ddd (10.0, 8.6, 2.4) | 83.7 | CH |
| 7 | 1.55 m, 2.04 m | 25.8 | $CH_2$ |
| 8 | 0.99 t (7.4) | 9.7 | $CH_3$ |
| 9 | 4.55 dd (9.4, 1.9) | 47.5 | CH |
| 10 | 5.49 dd (9.4, 2.0) | 74.3 | CH |
| 12 | – | 170.0 | C=O |
| 17 | 5.09 ddd (7.7, 6.0, 2.0) | 71.6 | CH |
| 19 | – | 170.7 | C=O |
| 21 | 2.13 s | 20.8 | $CH_3$ |
| 22 | 1.25 m, 1.51 m | 31.5 | $CH_2$ |

| | | | |
|---|---|---|---|
| 23 | 1.26 m | 24.7 | $CH_2$ |
| 24 | 1.26 m | 31.5 | $CH_2$ |
| 25 | 1.26 m | 22.4 | $CH_2$ |
| 26 | 0.86 t (7.0) | 13.9 | $CH_3$ |
| 27 | 2.17 s | 21.1 | $CH_3$ |

[a] The assignments were based on the COSY, TOCSY, HMQC, NOESY and HMBC experiments. [b] Coupling constants in Hz are given in parenthesis. [c] Multiplicities were assigned from the HSQC spectrum.

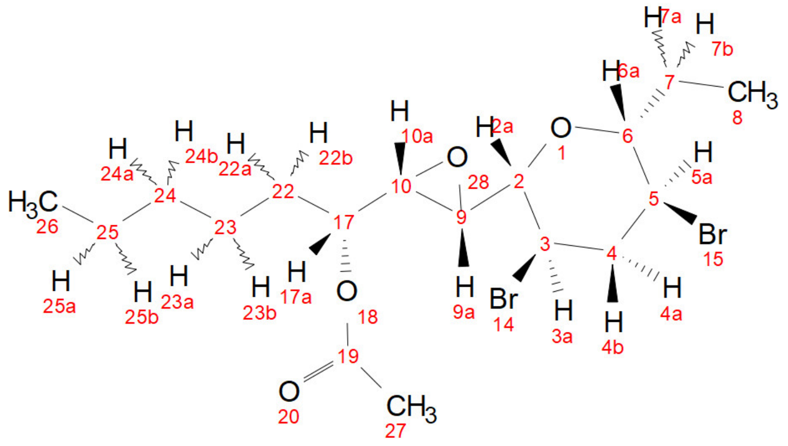


**9**

**Table S3.** $^1H$ and $^{13}C$ chemical shift data for compound **9** (700 MHz, $CDCl_3$)[a]

| position | $\delta_H$[b] | $\delta_C$ | mult[c] |
|---|---|---|---|
| 2 | 3.51 dd (10.0, 7.1) | 80.5 | CH |
| 3 | 3.84 ddd (12.2, 10.0, 4.5) | 46.1 | CH |
| 4 | b) 2.43 ddd (12.9, 12.2, 12.0)<br>a) 2.98 ddd (12.9, 4.5, 4.3) | 45.6 | $CH_2$ |
| 5 | 3.73 ddd (12.0, 10.0, 4.3) | 47.1 | CH |
| 6 | 3.45 ddd (10.0, 7.4, 2.7) | 83.0 | CH |
| 7 | 1.59 ddq (14.9, 7.4, 7.4)<br>1.98 ddq (14.9, 7.4, 2.7) | 25.8 | $CH_2$ |
| 8 | 0.95 t (7.4) | 9.0 | $CH_3$ |
| 9 | 3.02 dd (7.1, 4.1) | 57.6 | CH |
| 10 | 3.05 dd (8.7, 4.1) | 56.7 | CH |

| | | | | |
|---|---|---|---|---|
| 17 | 4.89 ddd (8.7, 8.7, 3.9) | 71.9 | CH | |
| 19 | – | 170.5 | C=O | |
| 22 | 1.69 m | 32.1 | $CH_2$ | |
| 23 | 1.31 m, 1.36 m | 24.6 | $CH_2$ | |
| 24 | 1.25 m | 31.8 | $CH_2$ | |
| 25 | 1.29 m | 22.5 | $CH_2$ | |
| 26 | 0.87 t (7.0) | 14.0 | $CH_3$ | |
| 27 | 2.10 s | 21.1 | $CH_3$ | |

[a] The assignments were based on the COSY, TOCSY, HMQC, NOESY and HMBC experiments. [b] Coupling constants in Hz are given in parenthesis. [c] Multiplicities were assigned from the HSQC spectrum.

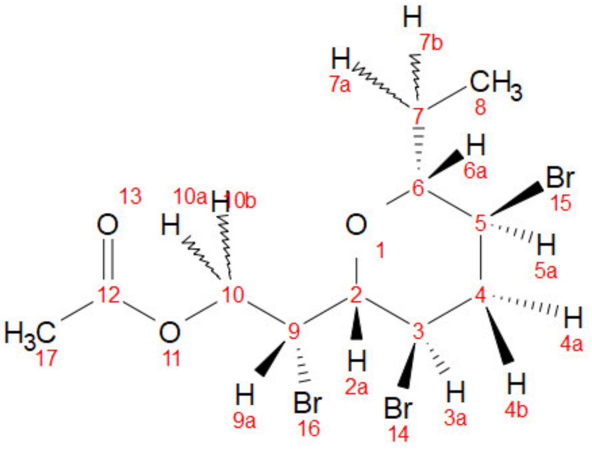

**10**

**Table S4**. $^{1}$H and $^{13}$C chemical shift data for compound **10** (700 MHz, $CDCl_3$)[a]

| position | $\delta_H$[b] | $\delta_C$ | mult[c] |
|---|---|---|---|
| 2 | 3.51 dd (9.7, 1.8) | 79.3 | CH |
| 3 | 4.10 ddd (12.1, 9.7, 4.5) | 46.6 | CH |
| 4 | b) 2.46 ddd (12.9, 12.1, 12.0)<br>a) 3.01 ddd (12.1, 4.5, 4.3) | 45.3 | $CH_2$ |
| 5 | 3.75 ddd (12.0, 10.0, 4.3) | 47.5 | CH |
| 6 | 3.43 ddd (10.0, 8.5, 2.5) | 83.7 | CH |
| 7 | 1.55 ddq (14.5, 8.5, 7.4)<br>2.03 ddq (14.5, 7.4, 2.5) | 25.8 | $CH_2$ |
| 8 | 0.96 t (7.4) | 9.4 | $CH_3$ |
| 9 | 4.60 ddd (8.3, 6.7, 1.8) | 49.8 | CH |
| 10 | 4.40 dd (11.3, 8.3)<br>4.43 dd (11.3, 6.7) | 64.8 | CH |
| 12 | – | 170.2 | CH |
| 17 | 2.11 s | 20.7 | C=O |

[a] The assignments were based on the COSY, TOCSY, HMQC, NOESY and HMBC experiments. [b] Coupling constants in Hz are given in parenthesis. [c] Multiplicities were assigned from the HSQC spectrum.

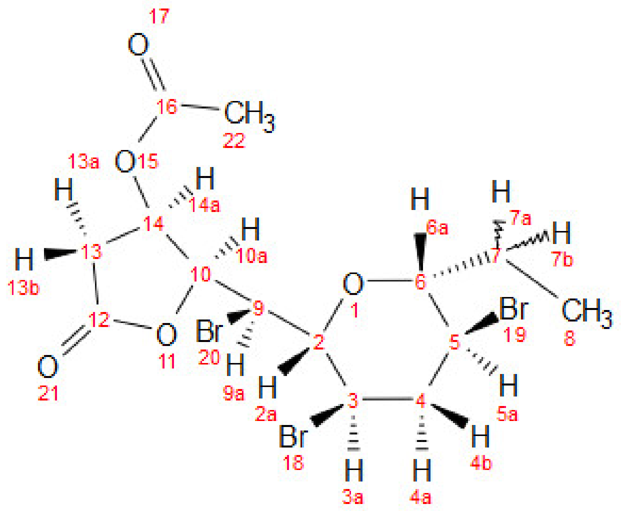


**11**

**Table S5**. $^{1}$H and $^{13}$C chemical shift data for compound **11** (700 MHz, $CDCl_3$)[a]

| position | $\delta_H$[b] | $\delta_C$ | mult[c] |
|---|---|---|---|
| 2 | 3.26 dd (9.5, 1.7) | 79.3 | CH |
| 3 | 4.19 ddd (12.1, 9.5, 4.6) | 46.1 | CH |
| 4 | b) 2.41 ddd (12.9, 12.1, 12.0)<br>a) 3.01 ddd (12.9, 4.6, 4.3) | 44.9 | $CH_2$ |
| 5 | 3.75 ddd (12.0, 10.5, 4.3) | 46.7 | CH |
| 6 | 3.42 ddd (10.5, 8.4, 2.5) | 83.7 | CH |
| 7 | 1.59 m, 2.04 m | 25.7 | $CH_2$ |
| 8 | 0.96 t (7.4) | 9.5 | $CH_3$ |
| 9 | 4.75 dd (9.8, 1.7) | 49.8 | CH |
| 10 | 4.85 dd (9.8, 3.3) | 83.0 | CH |
| 12 | – | 171.9 | C=O |
| 13 | b) 2.69 d (18.1)<br>a) 2.95 dd (18.1, 5.1) | 37.9 | $CH_2$ |
| 14 | 5.46 dd (5.1, 3.3) | 69.5 | $CH_2$ |
| 16 | – | 169.8 | C=O |
| 22 | 2.17 s | 21.0 | $CH_3$ |

[a] The assignments were based on the COSY, TOCSY, HMQC, NOESY and HMBC experiments. [b] Coupling constants in Hz are given in parenthesis. [c] Multiplicities were assigned from the HSQC spectrum.

© 2020 by the authors. Licensee MDPI, Basel, Switzerland. This article is an open access article distributed under the terms and conditions of the Creative Commons Attribution (CC BY) license (http://creativecommons.org/licenses/by/4.0/).
